# Meta-analysis of the prevalence of anxiety disorders in mainland China from 2000 to 2015

**DOI:** 10.1038/srep28033

**Published:** 2016-06-16

**Authors:** Xiaojing Guo, Zhen Meng, Guifeng Huang, Jingyuan Fan, Wenwen Zhou, Weijun Ling, Juan Jiang, Jianxiong Long, Li Su

**Affiliations:** 1 School of Public Health of Guangxi Medical University, Nanning, Guangxi, China; 2 Pre-Clinical Faculty of Guangxi Medical University, Nanning, Guangxi, China

## Abstract

Although anxiety disorders (ADs) have been recognized as one of the most prevalent mental disorders in mainland China, the prevalence of ADs has not been reported until now. The lack of a consolidated and comparable review on the prevalence of ADs in mainland China necessitated this meta-analysis to measure the prevalence. To identify the relevant studies on ADs for the analysis, we searched published studies in electronic databases up to July 2015. The pooled prevalence in the overall population and the prevalences by gender and location were estimated. A total of 21 studies were included in the analysis. The pooled current/lifetime prevalences of ADs, generalized AD, non-specific AD, panic disorder, social phobia, agoraphobia, specific phobia, post-traumatic stress disorder, and obsessive-compulsive disorder were 24.47‰/41.12‰, 5.17‰/4.66‰, 8.30‰/6.89‰, 1.08‰/3.44‰, 0.70‰/4.11‰, 0.19‰/2.15‰, 0.63‰/19.61‰, 0.49‰/1.83‰, and 0.90‰/3.17‰, respectively. Subgroup analyses indicated that compared with males, females had a consistently significantly higher prevalence of ADs. However, no difference was observed between those in urban and rural areas. The pooled prevalence of ADs was relatively lower than those of some other countries. A higher prevalence of ADs in women than in men was commonly observed, whereas the prevalences in urban and rural areas were nearly the same.

The 21st century is the age of anxiety^1^,^2^. Anxiety disorders (ADs, equivalent to ‘any AD’), as severe mental disorders with a high prevalence and inheritance, are characterized by feelings of anxiety (worries about the future) and fear (worries about the present) that can simultaneously cause physical symptoms such as increased blood pressure, quickened respiration and tightness of the chest^3^. The *Diagnostic and Statistical Manual of Mental Disorders*, version IV (DSM-IV), divides ADs into subtypes, including generalized anxiety disorder (GAD), non-specific AD (NSAD), panic disorder with or without agoraphobia, social phobia, specific phobia, post-traumatic stress disorder (PTSD), and obsessive-compulsive disorder (OCD)^3^. ADs impair patients’ social function, thereby affecting their quality of life and causing numerous societal burdens. For example, Japan’s burden due to ADs was estimated to be more than $20.5 billion in 2008 4. ADs are becoming nearly ubiquitous and concerning, causing severe social health problems associated with fear, nervousness, apprehension and panic and leading to disruption of the individual’s cardiovascular and respiratory systems^5^. Furthermore, a worldwide survey of the World Health Organization (WHO) showed that ADs are associated with numerous risk factors, such as educational level, average income, stressful life events, and multiple pains^6^,^7^,^8^. It is estimated that the global current prevalence of ADs is 7.3%, ranging from 0.9% to 28.3%, based on 87 studies in 44 countries^9^. The prevalence of ADs greatly varies throughout the world. Previous studies have indicated that ADs are the most prevalent psychiatric diseases in Europe (13.6%)^10^ and the United States (18.1%)^11^. However, a survey in Japan reported a lower prevalence of ADs, in which the lifetime and 12-month prevalences were 8.1% and 4.9%^12^, respectively. Similarly, the lifetime and 12-month prevalences of ADs were found to be 8.7% and 6.8%, respectively, in a Korea population^13^. Accordingly, more attention should be paid to ADs.

China, considered a developing country, has the largest population and highest degree of multinationality in the world. With its rapid societal and economic development, people’s quality of life has greatly improved, and consequently they pay more attention to their health and can afford medical services^14^. Two nationwide investigations on mental disorders were conducted in 1982 and 1993 in China^15^,^16^, but they did not address ADs. Since then, studies on ADs have been performed in several provinces of China. However, the results have been inconsistent. In Phillips’s study, the current prevalence of ADs in Shandong province was found to be 30.77‰, whereas in Zhejiang, it was 21.86‰^17^. In another study, conducted in Guangxi Zhuang Autonomous Region, both the current and lifetime prevalences of ADs were 1.26‰^18^ in 2007. Liu *et al*. conducted a study in Beijing in which the current and lifetime prevalences of ADs were found to be 31.59‰ and 59.54‰, respectively^19^. However, no epidemiological surveys on ADs at a national scale have been conducted in mainland China since 1993.

To the best of our knowledge, no previous systematic reviews on ADs in mainland China have been conducted. Moreover, it was not until 2000 that Chinese research provided a clear definition of anxiety disorders^20^. Thus, we performed the first meta-analysis of ADs in mainland China (excluding Hong Kong, Taiwan, and Macao) from 2000 to 2015, with a particular interest in estimating the pooled prevalence of ADs, investigating whether significant differences existed in gender (males/females) and location (urban/rural) and observing the differences by time and geographical distribution.

## Results

### Search results

**Figure 1:**
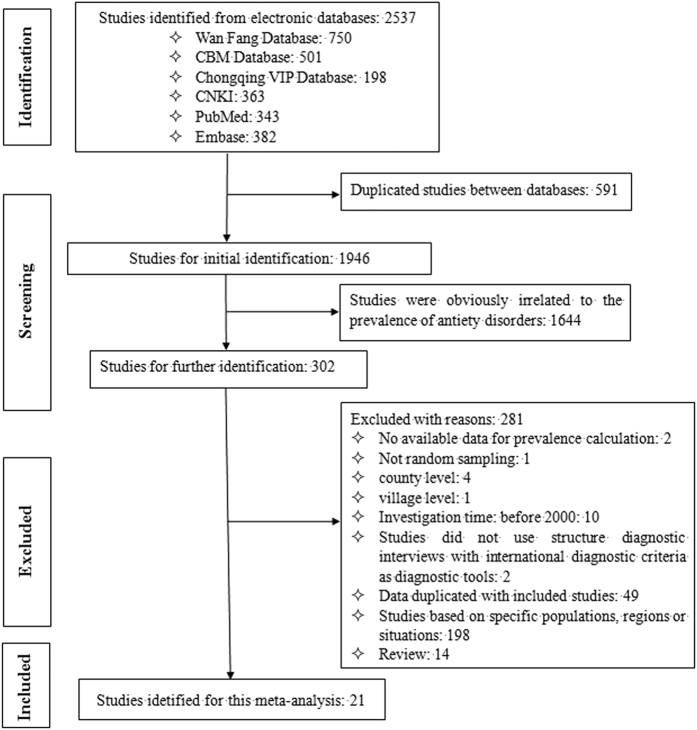
Procedure of the selection process.

A total of 2537 studies were initially retrieved using the search format described in the Materials and Methods section. However, 591 studies were excluded because of duplication between databases. Then, 1946 studies were selected for initial identification. Of these, 1644 studies were excluded because they focused on the treatment of mental disorders, the disability rate of mental disorders or the management of patients with mental disorders or others, which were clearly not related to the prevalence of anxiety disorders. The remaining 302 studies were further studied by carefully reading the full text. After the full text review, 281 studies were excluded for the following reasons: i) they did not provide data for prevalence calculation (n = 2); ii) they did not perform random sampling (n = 1); iii) they were conducted at the county (n = 4) or village level (n = 1); iv) they were conducted before 2000 (n = 10); v) for diagnostic tools, they did not use structured diagnostic interviews with international diagnostic criteria, such as the Composite International Diagnostic Interview (CIDI), the Structured Clinical Interview for the DSM-IV (SCID) or the Anxiety Disorder Interview Schedule (ADIS) (n = 2); vi) the data duplicated those of other included studies (n = 49); vii) they were based on specific populations, regions or situations (n = 198) or viii) they were reviews (n = 14). Ultimately, 21 studies^17^,^18^,^19^,^21^,^22^,^23^,^24^,^25^,^26^,^27^,^28^,^29^,^30^,^31^,^32^,^33^,^34^,^35^,^36^,^37^,^38^ were selected for this meta-analysis. Figure 1 illustrates the detailed search process.

### Study characteristics and assessment of study quality

As mentioned above, 21 studies were included in this meta-analysis. The years that these studies were conducted ranged from 2001 to 2012, and they covered 11 provinces (Fujian, Gansu, Guangdong, Hebei, Henan, Liaoning, Qinghai, Shandong, Yunnan, Zhejiang, and Shannxi), 2 municipalities (Beijing and Shanghai), and 3 autonomous regions (Ningxia, Guangxi and Tibet) in mainland China. Of the 21 studies, 11 were conducted at the provincial level and 10 were at the city level. With regard to the age of subjects, 8, 3, and 16 studies were based on individuals aged 15 years or above, 16 years or above, and 18 years or above, respectively. The CIDI was adopted in 11 studies, and the SCID was used in 10 studies. Table 1 and Supplementary Table 1 (Table S1) showed the characteristics of these studies.

All studies received an assessment score of at least 7. Specifically, studies obtained a score of 10 (n = 10), 9 (n = 5), 8 (n = 4) and 7 (n = 2). More details of the assessment of study quality are provided in Table S2.

### Selection of a fixed-effects or random-effects model

In this study, the current and lifetime prevalence of ADs and their subtypes in the overall population, by gender (males, females), and by location (urban, rural) were all estimated with a random-effects model. Both the fixed- and random-effects models were used in to identify gender or location differences; the results are presented in detail in Table 2.

### Prevalence of ADs and their subtypes

#### Anxiety disorders

##### Overall prevalence

**Figure 2:**
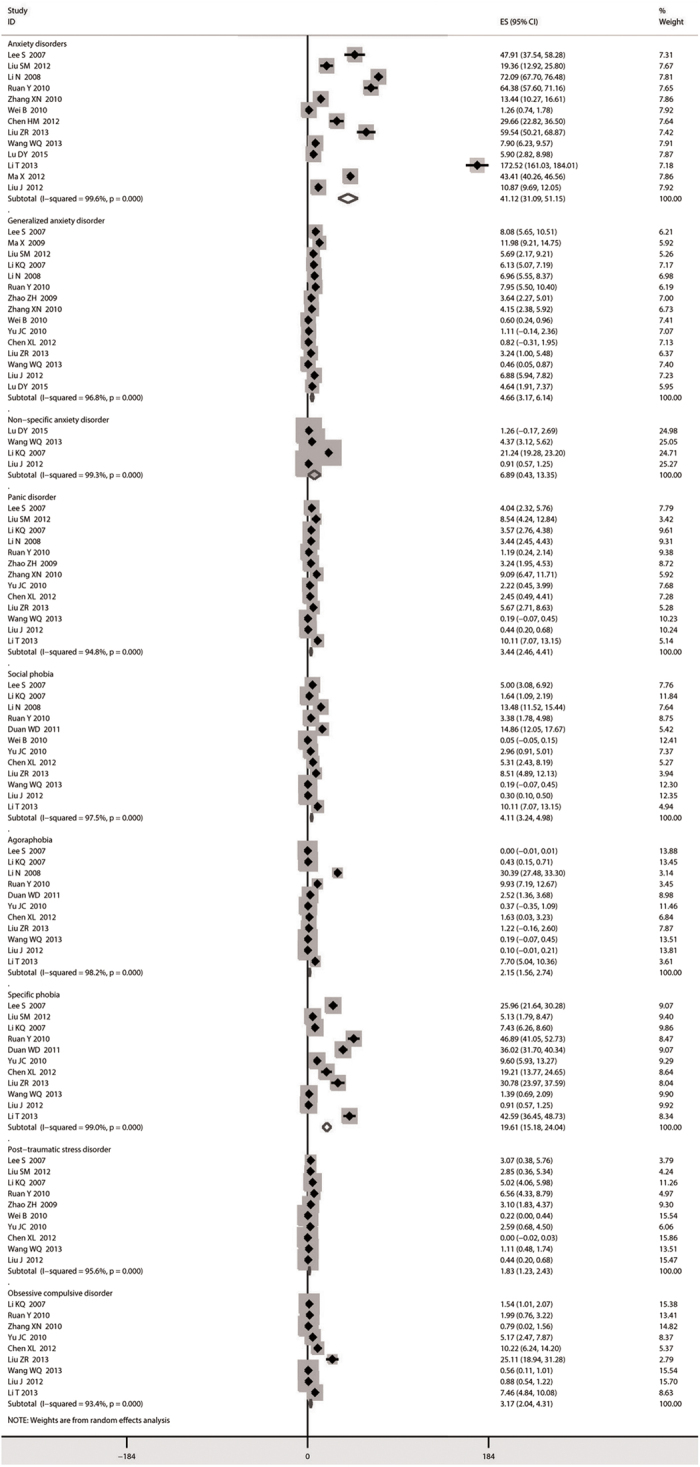
Forest plot for the lifetime prevalence rate of ADs and their subtypes.

**Figure 3:**
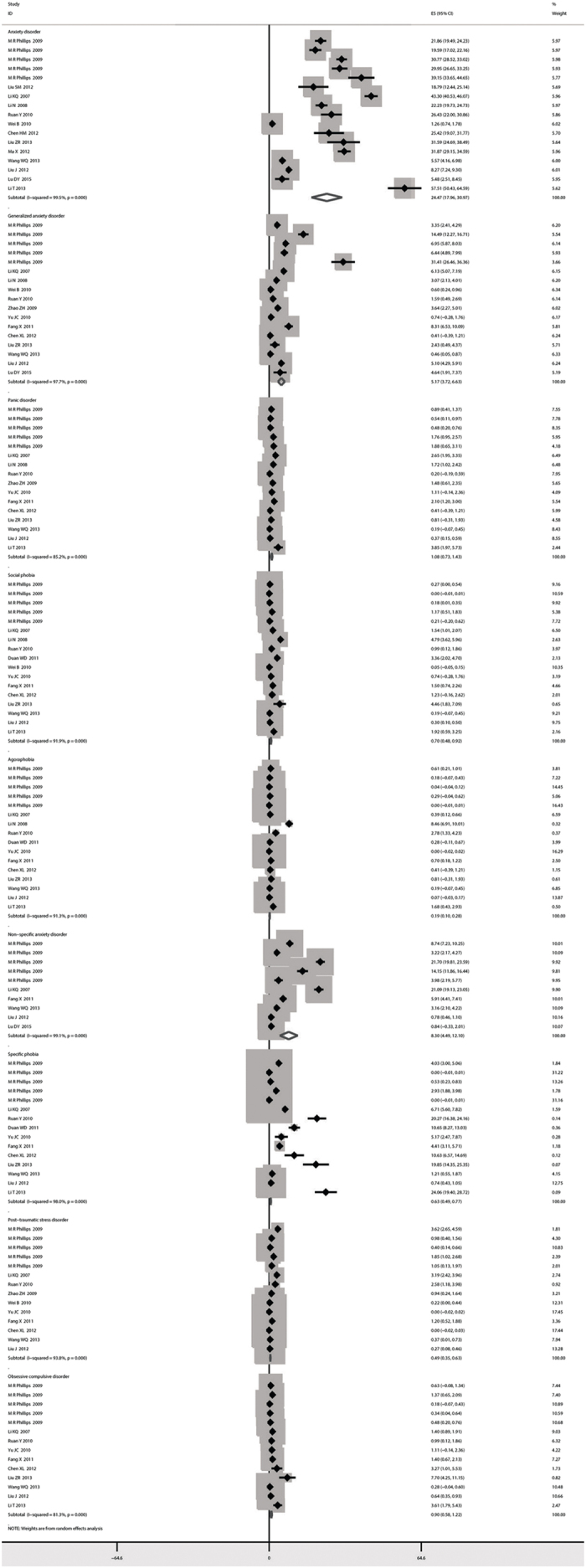
Forest plot of the current prevalence of ADs and their subtypes.

**Figure 4:**
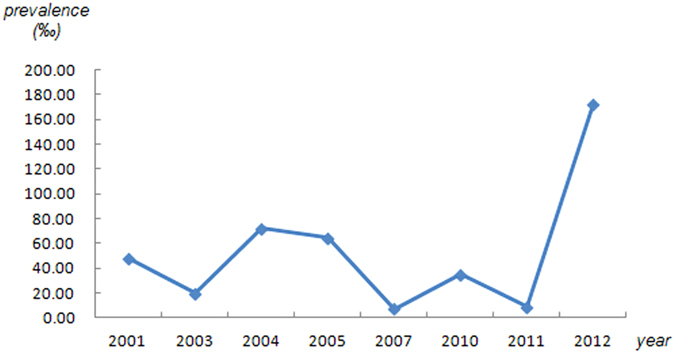
Temporal trends in the lifetime prevalence of ADs in mainland China.

**Figure 5:**
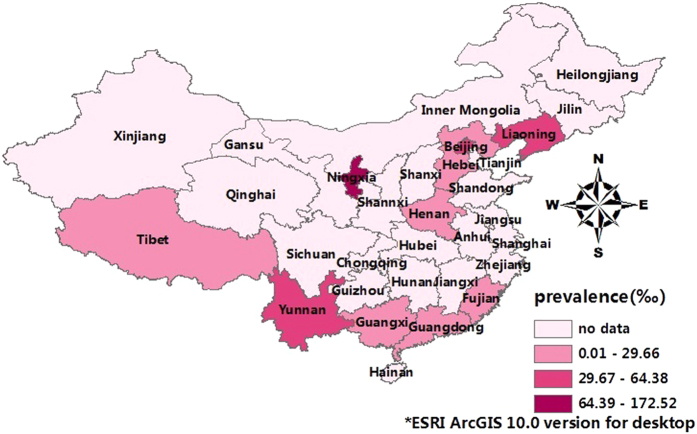
Regional distribution of pooled lifetime prevalences of ADs in different regions of mainland China (by ESRI ArcGIS 10.0 version for desktop, http://www.esri.com/software/arcgis/arcgis-for-desktop).

The lifetime prevalence of ADs was 41.12‰ (95% CI: 31.09–51.15) (Fig. 2), while the current prevalence was 24.47‰ (95% CI: 17.97–30.98) (Fig. 3). Figure 4 shows that no significant trend was detected in the temporal trend of the lifetime prevalence of ADs from 2001 to 2012. Varying prevalences of ADs were observed in different Chinese provinces, among which Ningxia had the highest current and lifetime prevalences and Guangxi had the lowest. The color-coded map in Fig. 5 illustrates the different degrees of the lifetime prevalence of ADs. This map was divided into four sections according to prevalence, from highest to lowest. However, the majority of the map zones indicated that these corresponding regions lacked available epidemiological data on ADs. Thus, no distribution difference could be detected in the color-coded map of mainland China.

##### Prevalence by gender

The current and lifetime prevalences of ADs were 15.37‰ (95% CI: 8.31–22.43) and 28.46‰ (95% CI: 8.63–48.29), respectively, for males and 25.74‰ (95% CI: 11.87–39.61) and 53.69‰ (95% CI: 16.74 – 90.65), respectively, for females (Table 2). Compared with females, males had a lower risk of developing Ads, with ORs of 0.57 (95% CI: 0.44–0.75) for current prevalence and 0.56 (95% CI: 0.43–0.73) for lifetime prevalence (Table 2).

##### Prevalence by location

Urban (current: 16.99‰, 95% CI: 3.40–30.58; lifetime: 37.97‰, 95% CI: 10.97–64.97) and rural (current: 17.68‰, 95% CI: 6.84–28.51; lifetime: 36.83‰, 95% CI: −0.32–73.99) locations had a similar prevalence of Ads (Table 2). No significant difference was found between urban and rural location, with ORs of 1.18 (95% CI: 0.76–1.84) for current prevalence and of 0.97 (95% CI: 0.62–1.51) for lifetime prevalence (Table 2).

#### Generalized anxiety disorder

##### Overall prevalence

The current and lifetime prevalence of GAD was 5.17‰ (95% CI: 3.72–6.63) (Fig. 3) and 4.66‰ (95% CI: 3.17–6.14), respectively (Fig. 2).

##### Prevalence by gender

The prevalence of GAD in males (current: 2.97‰, 95% CI: 1.83–4.12; lifetime: 0.43‰, 95% CI: 0.13–0.72) was lower than that in females (current: 6.32‰, 95% CI: 3.45–9.19; lifetime: 5.63‰, 95% CI: 2.59–8.66) (Table 2), indicating that males were less likely to suffer from GAD than females (OR, current: 0.44, 95% CI: 0.34–0.56; lifetime: 0.49, 95% CI: 0.38–0.65) (Supplementary Figs 1 and 2, Figs S1 and S2).

##### Prevalence by location

As for the prevalence of GAD by location, urban areas did not differ from rural areas. The current prevalence of GAD was 4.56‰ (95% CI: 3.45–5.66) in urban and 5.51‰ (95% CI: 2.73–8.29) in rural areas with an OR of 0.94 (95% CI: 0.61–1.45) (Fig. S3). Moreover, the estimated lifetime prevalence was 4.57‰ (95% CI: 2.09–7.06) in urban and 4.22‰ (95% CI: 1.76–6.68) in rural areas, with an OR of 1.07 (95% CI: 0.83–1.38) (Fig. S4).

#### Non-specific anxiety disorder

##### Overall prevalence

The overall estimated current prevalence of NSAD was 8.30‰ (95% CI: 4.49–12.10) (Fig. 3), while the lifetime prevalence was a few percentage points lower at 6.89‰ (95% CI: 0.43–13.35) (Fig. 2).

##### Prevalence by gender

The current prevalence of NSAD in males was 4.01‰ (95% CI: 1.98–6.05), while in females, it was 7.65‰ (95% CI: 2.96–12.34) (Table 2). A significant difference was found between males and females (OR = 0.51, 95% CI: 0.38–0.67) (Fig. S1).

##### Prevalence by location

The current prevalence of NSAD was 7.60‰ (95% CI: 2.19–13.01) in urban areas and 4.66‰ (95% CI: 1.94–7.37) in rural areas (Table 2). The OR confirmed a similar rate in urban and rural areas, with a value of 1.61 (95% CI: 0.80–3.24) (Fig. S3).

#### Panic disorder

##### Overall prevalence

The pooled prevalence of panic disorder for current prevalence was 1.08‰ (95% CI: 0.74–1.43) (Fig. 3), while the lifetime prevalence was 3.44‰ (95% CI: 2.46–4.41) (Fig. 2).

##### Prevalence by gender

The current and lifetime prevalences of panic disorder in males were 1.16‰ (95% CI: 0.49–1.84) and 2.30‰ (95% CI: 1.07–3.54), respectively, while in females, they were 2.01‰ (95% CI: 0.67–3.34) and 4.53‰ (95% CI: 2.01–7.05), respectively (Table 2). Compared with females, males seemed to have a lower risk of developing panic disorder (current: OR = 0.50, 95% CI: 0.32–0.77; lifetime: OR = 0.49, 95% CI: 0.33–0.72 (Figs S1 and S2).

##### Prevalence by location

The current prevalence of panic disorder in urban and rural areas was 1.10‰ (95% CI: 0.49–1.72) and 1.97‰ (95% CI: 0.51–3.43), respectively, while the lifetime prevalences were 3.18‰ (95% CI: 1.17–5.19) and 3.19‰ (95% CI: 1.20–5.18), respectively (Table 2). The prevalence in urban areas did not differ from that in rural areas. The ORs were 0.64 (95% CI: 0.35–1.18) for current and 0.82 (95% CI: 0.56–1.21) for lifetime prevalence (Figs S1 and S2).

#### Social phobia

##### Overall prevalence

The current and lifetime prevalences of social phobia were estimated to be 0.70‰ (95% CI: 0.48–0.92) (Fig. 3) and 4.11% (95% CI: 3.24–4.99) (Fig. 2), respectively.

##### Prevalence by gender

The current and lifetime prevalences of social phobia in males were 1.94‰ (95% CI: 0.83–3.05) and 5.68‰ (95% CI: 2.26–9.10), respectively, and 2.21‰ (95% CI: 1.15–3.28) and 7.63% (2.28–12.98), respectively, in females (Table 2). The ORs were 0.82 (95% CI: 0.53–1.27) for current prevalence and 0.77 (95% CI: 0.62–0.96) for lifetime prevalence (Figs S1 and S2), indicating that the current prevalence in males was similar to the current prevalence in females, while the lifetime prevalence in males was significantly lower than that in females.

##### Prevalence by location

The current prevalence of social phobia in urban and rural areas was 1.56‰ (95% CI: (0.59–2.52) and 1.52‰ (95% CI: 1.03–2.01), respectively, while the lifetime rates were 4.18‰ (95% CI: 0.60–7.77) and 5.70‰ (95% CI: 0.33–11.07), respectively (Table 2). We found no significant difference between urban and rural areas in current prevalence, with an OR of 1.04 (95% CI: 0.50–2.16). In contrast, urban areas had a lower risk of lifetime prevalence than rural areas, with an OR of 0.68 (95% CI: 0.51–0.90) (Figs S3 and S4).

#### Agoraphobia

##### Overall prevalence

The overall current prevalence of agoraphobia was 0.19‰ (95% CI: 0.10–0.28) (Fig. 3), and the lifetime prevalence was 2.15‰ (95% CI: 1.56–2.74) (Fig. 2).

##### Prevalence by gender

The current and lifetime prevalences of agoraphobia in males were 0.00‰ (95% CI: −0.01–0.01) and 0.21‰ (95% CI: −0.05–0.47), respectively, while in females, they were 0.77‰ (95% CI: 0.03–1.52) and 11.09‰ (95% CI: 3.41–18.76), respectively (Table 2). Females suffered more frequently from agoraphobia than males. Significant ORs of 0.17 (95% CI: 0.04–0.76) for current prevalence and 0.34 (95% CI: 0.28–0.42) for lifetime prevalence were found (Figs S1 and S2).

##### Prevalence by location

The current prevalence of agoraphobia was 0.41‰ (95% CI: −0.16–0.98) in urban areas and 0.71‰ (95% CI: 0.18–1.24) in rural areas (Table 2), with an OR of 0.58 (95% CI: 0.12–2.80) (Fig. S3). Additionally, the lifetime prevalence was 5.23‰ (95% CI: 0.71–9.75) in urban and 10.43‰ (95% CI: −1.06–21.92) in rural areas (Table 2), with an OR of 0.52 (95% CI: 0.17–1.60) (Fig. S4), indicating that no significant differences between urban and rural areas were found.

#### Specific phobia

##### Overall prevalence

For specific phobia, the current prevalence was 0.63‰ (95% CI: 0.49–0.77) (Fig. 3), and the lifetime prevalence was 19.61‰ (95% CI: 15.18–24.04) (Fig. 2).

##### Prevalence by gender

In this study, 3.37‰ (95% CI: 1.78–4.95) and 9.39‰ (95% CI: 5.23–13.55) were calculated as the current prevalences of specific phobia in males and females, respectively, while 17.91‰ (95% CI: 8.48–27.33) and 37.14‰ (95% CI: 19.19–55.09) were calculated as the lifetime prevalences of specific phobia in males and females, respectively (Table 2). When compared to females, males had a lower risk of suffering from specific phobia than females (current: OR = 0.43, 95% CI: 0.35–0.54; lifetime: OR = 0.47, 95% CI: 0.40–0.56) (Figs S1 and S2).

##### Prevalence by location

The current and lifetime prevalences of specific phobia in urban versus (v. or vs.) rural areas were 3.25‰ (95% CI: 1.07–5.44) v. 3.58‰ (95% CI: 0.56–6.59), respectively, and 25.84‰ (95% CI: −2.13–49.56) v. 23.60‰ (95% CI: 4.88–42.32), respectively (Table 2). No significant differences were detected between location (urban/rural) in both current (OR = 1.05, 95% CI: 0.21–5.35) and lifetime prevalence (OR = 0.96, 95% CI: 0.37–2.48) (Figs S3 and S4).

#### Post-traumatic stress disorder

##### Overall prevalence

The current and lifetime prevalences of PTSD in the overall population were 0.49‰ (95% CI: 0.35–0.63) (Fig. 3) and 1.83‰ (95% CI: 1.23–2.43), respectively (Fig. 2).

##### Prevalence by gender

Though the prevalence of PTSD in males (current: 2.42‰, 95% CI: 1.69–3.14; lifetime: 0.07‰, 95% CI: −0.10–0.23) was slightly lower than that in females (current: 4.26‰, 95% CI: 2.88–5.64; lifetime: 0.89‰, 95% CI: −0.09–0.11) (Table 2), no significant difference was found between males and females (current: OR = 0.56, 95% CI: 0.32–1.00; lifetime: OR = 0.89, 95% CI: 0.12–6.74) (Figs S1 and S2).

##### Prevalence by location

Interestingly, urban (2.97‰, 95% CI: −0.03–5.96) and rural (3.34‰, 95% CI: 2.66–4.01) locations did not differ in the risk of developing PTSD, with an OR of 0.86 (95% CI: 0.26–2.81) for the current prevalence (Table 2). However, urban (0.01%, 95% CI: −0.08–0.10) locations had less risk of suffering from PTSD than rural ones (1.98‰, 95% CI: −13.62–17.57), with an OR of 0.39 (95% CI: 0.20–0.76) (Figs S3 and S4).

#### Obsessive-compulsive disorder

##### Overall prevalence

The prevalence of OCD was 0.90‰ (95% CI: 0.58–1.22) (Fig. 3) for the current prevalence and 3.17‰ (95% CI: 2.04–4.31) for the lifetime prevalence (Fig. 2).

##### Prevalence by gender

The current prevalence of OCD was 1.11‰ (95% CI: 0.08–2.15) in males and 1.72% (95% CI: −0.10–3.54) in females, while the lifetime prevalence was 3.55‰ (95% CI: 1.01–6.08) and 8.71‰ (95% CI: 3.64–13.79) in males and females, respectively (Table 2). Males and females were similar in current prevalence of OCD, with an OR of 0.87 (95% CI: 0.51–1.51), but the males were at a lower risk of developing OCD than females, with an OR of 0.54 (95% CI: 0.36–0.82) (Figs S1 and S2).

##### Prevalence by location

No significant difference was observed in the lifetime prevalence of OCD between urban and rural (OR = 0.68, 95% CI: 0.39–1.18) areas (Fig. S3), as the prevalences in urban and rural locations were 2.77‰ (95% CI: 0.37–5.18) and 4.19‰ (95% CI: 1.20–7.19), respectively (Table 2). However, those in urban areas had a 2.32 times higher risk of developing OCD than those in rural areas (OR = 2.32, 95% CI: 1.08–4.99) (Fig. S4), as the current prevalences in urban and rural areas were 1.41‰ (95% CI: −0.51–3.32) and 0.69‰ (95% CI: −0.31–1.69), respectively (Table 2).

#### Meta-regression

There was remarkable heterogeneity in the identified studies in this meta-analysis (all I^2^ > 50%, ranging from 81.30% to 99.60%; *p* < 0.001). No possible sources of heterogeneity for the current and lifetime prevalences of ADs were detected. The potential sources of heterogeneity in the current prevalence of specific phobia were sample size (*p* = 0.047), province (Ningxia: *p* = 0.017, Yunnan: *p* = 0.026) and diagnostic tool used (CIDI: *p* < 0.001), while a potential source of the lifetime prevalence was diagnostic tool used (*p* = 0.040). In addition, year of investigation was associated with heterogeneity in the lifetime prevalence of GAD (*p* = 0.014) and PTSD (*p* = 0.030), while year of investigation (*p* = 0.009) and province (Guangdong: *p* = 0.028, Henan: *p* = 0.032, Shandong: *p* = 0.027, Shannxi: *p* = 0.026) were the sources of heterogeneity of the current prevalence of PTSD. The potential sources of the current prevalence of agoraphobia were the identity of the investigators (*p* = 0.022) and province (Liaoning: *p* = 0.006). Moreover, the identity of the investigators (OCD, others: *p* = 0.032; social phobia, others: *p* = 0.037) and the diagnostic tool used (OCD, CIDI: *p* = 0.048; social phobia, CIDI: *p* = 0.037) should both be considered sources of heterogeneity in the current prevalence of OCD and social phobia. More details are given in Table S3.

#### Sensitivity analysis

The results showed that none of the studies influenced the pooled current prevalence of panic disorder, specific phobia, PTSD, or OCD. However, the results of the current prevalences of ADs, GAD, NSAD, social phobia, and agoraphobia were affected after several single studies were omitted. As for the lifetime prevalence of ADs and their subtypes, all of them yielded inconsistent results when some individual studies were removed. Table S4 presents the detailed results of the sensitivity analyses.

#### Publication bias

Based on the asymmetric shape of the funnel plots, Egger’s and Begg’s tests showed a significant value (*p* < 0.05) for current and lifetime prevalences of ADs and most AD subtypes (Table S5).

## Discussion

To the best of our knowledge, this meta-analysis is the first to report the prevalence of ADs and their subtypes in mainland China. The current and lifetime prevalences were, respectively, 24.47‰ and 41.12‰ for ADs, 5.17‰ and 4.66‰ for GAD, 8.30‰ and 6.89‰ for NSAD, 1.08‰ and 3.44‰ for panic disorder, 0.70% and 4.11‰ for social phobia, 0.19% and 2.15% for agoraphobia, 0.63‰ and 19.61‰ for specific phobia, 0.49‰ and 1.83% for PTSD, and 0.90‰ and 3.17‰ for OCD. Compared with males, females had a higher risk of developing ADs and their subtypes except for PTSD. No significant differences were found in ADs and most of the subtypes between urban and rural areas. However, individuals in urban areas were likely to have a lower risk of lifetime prevalence of social phobia and PTSD and a higher risk of OCD than those in rural areas.

### ADs and their subtypes

Compared with other countries, the prevalence of ADs was lower in mainland China. The lifetime prevalence of ADs was found to be 28.8% in the United States^11^ and 20.0% in Australia^39^, which were approximately 7 and 5 times, respectively, the lifetime prevalence (41.12‰) in mainland China. Moreover, the twelve-month prevalence of ADs was reported to be 6.8% in Mexico^40^. Regarding other eastern Asian countries, the lifetime prevalence of ADs in Japan was 8.1%^12^, while in South Korea, it was 8.7%^13^, which were both higher than that in mainland China. In addition, with respect to Hong Kong, the 12-month prevalence of GAD was estimated to reach 5.04%^41^. Therefore, compared to western developed countries, Asian countries were likely to have a lower prevalence of ADs. The comparative results were consistent with previous studies that suggested that the prevalence of ADs was lower in Asian countries and in less developed countries^13^,^40^,^42^. Several probable factors could lead to these differences. First, the more pronounced perceived stigma in developing countries might be associated with the lower detected rate of ADs in mainland China than in some developed countries^6^. Second, a high rate of misdiagnosis of ADs^43^ and ADs comorbid with other mental disorders^44^ may have affected the lower prevalence of ADs in mainland China. Third, it was noteworthy that the diagnostic tools used (e.g., SCID and CIDI) and the diagnostic criteria (e.g., DSM and ICD) were based mostly on residents of Western countries, which might explain the disparity in prevalence in mainland Chinese given their inability to rephrase or understand the criteria in the context of their different cultural backgrounds^9^,^20^ and given potential issues with translation. Although the validity of the Chinese version of the CIDI-3.0 was found to be acceptable in diagnosing ADs and some other mental disorders^45^, some studies have found that international diagnostic criteria fail to detect some positive symptoms owing to a lack of sensitivity to the way in which complaints are manifested in the mainland Chinese population because of culture differences; for example, the mainland Chinese population tends to interpret their emotions physically, using somatizations to express physical problems^46^,^47^. As a result, the prevalence may have been underestimated. Fourth, age may be a risk factor in the emergence of ADs. Though ADs are believed to follow a chronic course, the prevalence of ADs has been found to decrease with age^2^. Setting the minimum age at 15 years old was a result of the World Health Organization’s suggestion that youth who are 15 years of age or older can experience full-fledged anxiety disorders^48^. In the present study, the age ranges in the studies were not all the same, and, interestingly, as the results showed, a wide range of prevalences was found in ADs and specific phobia but not in social phobia and GAD. Here, age may be taken into consideration. It is thought that the age of onset of ADs differs. The median age of onset in ADs has been reported to be 11 years^49^ The earliest median age at onset of specific phobia was reported to be 7 years, and for social anxiety disorder and GAD, this value was 13 and 31 years, respectively^2^. However, the age of the subjects included in our study was 15 years old and older, with the mean age ranging from 32.49 to 53 years; this might have affected the prevalence and could limit the representativeness of the prevalence in the population. Moreover, specific phobia had the highest prevalence reported in previous studies^11^,^13^,^49^,^50^,^51^,^52^; however, GAD has been reported to be an independent disorder with remarkable stable lifetime prevalence in the general population^53^,^54^.

### Gender difference

Females have a higher prevalence of ADs than males, as reported in previous studies^55^,^56^. Similar to our study, in a systematic review of the global prevalence of common mental disorders, Steel *et al*. found the strongest evidence that gender affects the occurrence of ADs, with an estimated lifetime prevalence of 4.3% for males and 8.7% for females^42^. A study in 15 countries showed that females were at an approximately 2.1 times higher risk than males of developing ADs, indicating that gender is a risk factor in ADs^56^. Though most ADs were found to be present in more females than in males (males vs. females, OR < 1), no significance difference was found in PTSD. Several explanations of this predominance of ADs among females may help interpret our findings. (1) It has been suggested that the female reproductive cycle may contribute to the significantly higher prevalence of ADs in women^5^,^57^. The intensive fluctuations in oestrogen and progesterone during the menstrual cycle, pregnancy or postpartum periods have been related to alterations in the neuroprotective effects of the hormone, which could increase the chronicity correlated with the occurrence of ADs^5^. (2) The lower risk of developing ADs in males has seemed to be related to differential access to appropriate health services^56^. Wang *et al*. demonstrated that although females were more likely to access health care treatment, they were less likely to receive mental health care treatment than males. Moreover, men have been reported to be more likely to turn to a professional mental health specialist for help if they were suffering from an AD^58^. (3) Several factors, such as environmental, genetic and physiological factors, may play a key role in the differences between females and males in AD development^59^,^60^,^61^. (4) PTSD was the only anxiety disorder that showed no significant difference between males and females in the present study. Exposure to trauma has been reported to be associated with PTSD development^62^. However, males and females differ in the types of trauma to which they are exposed. Males are exposed to more combat, physical attacks, threats and kidnapping, while females are more subject to sexual assault, sexual molestation, rape, childhood physical abuse and childhood parental neglect^54^. Though women have been reported to have a higher prevalence of developing ADs and to have a more chronic course in PTSD than men, gender has not been shown to differ in the persistence of ADs^56^. Moreover, the misdiagnosis of an AD with another AD, bulimia nervosa or major depressive disorder seems to predominantly affect females more so than males, which may have led to the results showing no gender differences in PTSD^51^,^63^.

### Location differences

Similar to our study, no significant differences between urban and rural areas were reported by Baxter *et al*., although the prevalence of ADs worldwide has been shown to be higher in rural (16.9%) than urban (8.4%)^9^ areas. A study on the prevalence of mental disorders in Korea showed that urban residence did not differ from rural residence in prevalence of ADs^13^. However, Peen *et al*. found an urban-rural difference in ADs (OR = 1.21, 95% CI: 1.02–1.21)^64^. In addition, William *et al*. demonstrated that ADs were significantly more prevalent among urban dwellers^65^. As for the differences found in social phobia, PTSD and OCD, the environment, social status, economic level and medical conditions may be relevant. A high risk of trauma, lack of social insurance support after trauma, low educational level, poor economic conditions and limited access to health services may increase the susceptibility of rural residents to PTSD and social phobia^66^,^67^. By contrast, the heavy workload, fierce competition and higher educational level of urban dwellers may contribute to the higher risk of OCD^65^.

### Sources of heterogeneity

A wide range of heterogeneity existed in the prevalence results of the included studies. Heterogeneity could not be avoided in the meta-analyses, especially those based on cross-sectional studies^67^. The sources (e.g., year of investigation, province, sample size, identity of investigators and diagnostic tool) were considered to be associated with heterogeneity using meta-regression. With respect to significant heterogeneity, we used random effects models in the meta-analysis. Additionally, regarding the potential sources of heterogeneity, we further analysed these sources by subgroup (including the identification of investigators and diagnostic tool used).

### Sensitivity analysis

The outputs of the pooled prevalences were influenced after five studies (“Wei B 2010”^18^, “Liu J”^32^, “Lee S”^22^, “M R Phillips”^17^ and “Wang WQ”^35^) were removed. When we reviewed these studies in detail and compared them with the others, we found that their prevalences were clearly lower than the average and that they had relatively large sample sizes, which may have resulted in the significant change to the results. Moreover, their quality scores were either 9 or 10, which demonstrated that the larger sample sizes and lower risk of bias may have contributed to the lower prevalence identified in this study.

### Publication bias

All included studies were eligible for the meta-analysis. Publication bias was determined to be present in this meta-analysis, although a relatively comprehensive search strategy was applied to identify the correlated studies. The fact that only studies published in Chinese and/or English were included in our study may have played a substantial role in the publication bias.

### Limitations

Some limitations should be noted in the interpretation of the results of this meta-analysis. First, the absence of cross-sectional investigations on ADs in 15 regions (11 provinces: Heilongjiang, Jilin, Shanxi, Anhui, Jiangxi, Jiangsu, Hunan, Hubei, Sichuan, Guizhou and Hainan; 2 municipalities: Tianjin and Chongqing; 2 autonomous regions: Inner Mongolia and Xinjiang) may affect the representativeness of the results. Second, different methodologies were used in the different studies, which may have contributed to some of the differences. Third, some studies failed to provide all the necessary data for the analysis. In addition, the strict confinement to the selection criteria, such as the investigation year, areas of mainland China and populations, may have partially caused the disparity in the results of this study. Finally, geographical differences such as different customs, different cultural issues, and even different local policies of governments can be considered a limitation.

In conclusion, the pooled current (24.47‰) and lifetime (41.12‰) prevalences of ADs were estimated for the first time by this meta-analysis. Generally, females had a higher risk of developing ADs than males, whereas urban residence did not differ from rural residence. The uniform methodology and sufficient data played an essential role in estimating the prevalences of ADs and their subtypes. Thus, epidemiological surveys on the prevalence of ADs using uniform methodology (screening tools, diagnostic tools and diagnostic criteria) should be conducted in regions throughout mainland China, which could result in uniform statistical magnitude in future studies. As a result, better evidence could be provided by meta-analyses regarding the management, prevention and control of ADs and their subtypes in mainland China and even throughout the world.

## Materials and Methods

### Literature search

A systematic and comprehensive literature search strategy was used to identify related studies for this meta-analysis in several electronic databases, including PubMed, Embase, Chinese National Knowledge Infrastructure, Chinese Biological Medical Literature Database, Chongqing VIP database for Chinese Technical Periodicals, and Wanfang Databases from their inception to July 17, 2015. The following key words were used when searching the Chinese databases: ‘anxiety disorder’, ‘generalized anxiety disorder’, ‘non-specific anxiety disorder’, ‘panic disorder’, ‘social phobia’, ‘agoraphobia’, ‘specific phobia’, ‘post-traumatic stress disorder’, ‘epidemiological’, ‘prevalence’ and ‘report’. However, when searching the English databases, we used not only the key words that were used in the Chinese databases but also their abbreviations (e.g., ‘AD’, ‘GAD’, ‘NSAD’, ‘PTSD’ and ‘OCD’), ‘China’ and ‘meta-analysis’. Subsequently, studies published in Chinese or English were included. In addition, to best avoid overlooking a related study, reference lists and contents were also retrieved.

### Study selection

The following criteria were used to select the papers for the analysis:

- Cross-sectional study conducted in mainland China (excluding Hong Kong, Macao, and Taiwan);
- Studies based on the general population;
- Studies published either in Chinese or English;
- Studies that directly provide the prevalence of ADs or studies that indirectly provide the relevant data for computing the prevalence. As for the relevant data, they included the number of total study participants and the number of AD cases.
- Studies used random sampling (excluding census sampling);
- Studies conducted at the territorial level (i.e., city level or above);
- Studies that adopted structure diagnostic interviews with international diagnostic criteria as the diagnostic tools, such as the SCID, CIDI or ADIS;
- Studies that included study subjects aged 15 years or older.

Studies that met any of the following criteria were excluded from the analysis:

- Studies that did not provide available rates or data for the prevalence calculation;
- Studies that were conducted before 2000;
- Studies that were conducted in specific areas or based on specific populations. Special areas included areas after an earthquake, hospitals and iron mine factories. With respect to specific populations, these could include troops, criminals, women only or others.
- Studies that did not explain the sampling method;
- Studies that were duplicated or contained in another study.

The study selection process was conducted by two researchers (Guo XJ and Meng Z) independently. If disagreements occurred, the researchers discussed the issue or the third researcher (Su L) became involved to reach a consensus.

### Data extraction and assessment of study quality

After a consensus on the included studies was obtained, data were extracted from the studies, including author name, publication year, survey dates, province, territorial level, location (urban/rural), sampling methods, sample size (overall; males/females; urban/rural), ages included, screening tools, diagnostic tools and criteria, effective response rate, name of investigators, and the current and/or lifetime cases/prevalence of ADs and eight common subtypes.

The quality of the studies was evaluated using the “Strengthening the Reporting of Observational Studies in Epidemiology” (STROBE) guidelines^68^, a tool primarily used to assess the risk of selection and performance bias. The STROBE guidelines contain 5 items with a total score of 10 (each item: low risk = 2, moderate risk = 1, high risk = 0). The aggregate scores represented the risk of bias.

Two researchers (Guo XJ and Meng Z) completed the work independently, and mutual discussion or a third researcher (Su L) were involved when disagreements occurred in making the final decision.

### Statistical analysis

In the present meta-analysis, the statistical software programmes STATA 12.0 version (Stata Corporation, College Station, TX, USA), Review Manager Version 5.2 (RevMan 5.2; The Cochrane Collaboration, The Nordic Cochrane Centre, Copenhagen, Denmark) and ESRI ArcGIS 10.0 version for desktop (http://www.esri.com/software/arcgis/arcgis-for-desktop) were used. The pooled prevalence estimates and their 95% confidence intervals (95% CI) were conducted by The DeSimonian and Laird method (14). The prevalences were presented as percentages, and “0.001” was used instead of “0” if the studies did not cite any cases to protect valid data from exclusion in the process of calculation. Heterogeneity was assessed by Cochran’s *x*^2^-based Q test and I^2^ statistics. The I^2^ statistics ranged from 0assessed by Cochran’s *x*^2^-based Q test and I^2^ statistics. The I^2^ statistics ranged from 0 to 100%, and p < 0.1 or I^2^ ≥ 50% was considered to indicate moderate or high heterogeneity. A random-effects model (The DeSimonian and Laird method) was used when a significant Q test (p < 0.1 or I^2^ ≥ 50%) occurred. Otherwise, a Mantel-Haenszel fixed-effects model was used. Gender (males/females) and location (urban/rural) differences were analysed using RevMan 5.2, with which the odds ratios and their 95% CIs were calculated. There are 5 levels of regions in China: province, prefecture, county, township, and village. The Administrative territory distribution uses a layered structure. At present, there are 34 provincial, 333 prefectural, 2862 county, and 41636 township level regions in China^69^,^70^ (details are shown in Fig. S5). Currently, mainland China (excluding Hong Kong, Macao, and Taiwan) is composed of 23 provinces, 4 municipalities and 5 autonomous regions. With respect to the differences in geographical distribution of mainland China, ESRI ArcGIS 10.0 version software for desktop was utilized. Furthermore, a meta-regression was performed to explore the sources of heterogeneity, and a sensitivity analysis was conducted to analyse the effects of single studies on the pooled prevalence after sequentially excluding individual studies; in other words, this process detected the robustness of a single study in the combined prevalence after omitting the included studies one by one each time. Moreover, the funnel plots and Egger’s test played an essential role in assessing the publication bias present in our study.

## Additional Information

**How to cite this article**: Guo, X. *et al*. Meta-analysis of the prevalence of anxiety disorders in mainland China from 2000 to 2015. *Sci. Rep*. **6**, 28033; doi: 10.1038/srep28033 (2016).

## Supplementary Material

Supplementary Information

## Figures and Tables

**Table 1:** Baseline characteristics of the studies included in the meta-analysis of Ads.

References	Year	≥Age	Mean age (SD)	Location	Territorial level	U&R*	Sampling method	Sample	Screening tools	Diagnostic tools	Diagnostic criteria	identity of investigator
MR Phillips^17^	2001	18	43.2(17.2)	Zhejiang	provincial	U&R	multistage stratified random	14639	GHQ-12	SCID-I/P	DSM-IV	doctor and nurse of psychiatry department
	2002	18	–	Qinghai	provincial	U&R	multistage stratified random	11178	GHQ-12	SCID-I/P	DSM-IV	doctor and nurse of psychiatry department
	2004	18	46(15)	Shandong	provincial	U&R	multistage stratified random	22718	GHQ-12	SCID-I/P	DSM-IV	doctor and nurse of psychiatry department
	2005	18	45(13)	Gansu	city	U&R	multistage stratified random	10249	GHQ-12	SCID-I/P	DSM-IV	doctor and nurse of psychiatry department
	2005	18	–	Shandong	city	U&R	multistage stratified random	4776	GHQ-12	SCID-I/P	DSM-IV	doctor and nurse of psychiatry department
Lee S^22^	2001	18	–	Beijing/shanghai	provincial	U	multi-stage cluster random	1628	CIDI	CIDI	DSM-IV	psychiatrist
	2001	18	–	Beijing/shanghai	provincial	U	multi-stage cluster random	570	CIDI	CIDI	DSM-IV	psychiatrist
	2001	18	–	Beijing/shanghai	provincial	U	multi-stage cluster random	5201	CIDI	CIDI	DSM-IV	psychiatrist
Ma X^27^	2003	15	–	Beijing	provincial	U&R	stratified, multi-stage systematic selection	5926	CIDI1.0	CIDI1.0	ICD-10	psychiatrist
Liu SM^26^	2003	15-59	–	Tibet	Provincial	U&R	classification quota random cluster	1756	neurosis screening table	SCID-I/P	DSM-IV	psychiatrist & non-psychiatric doctor
Li KQ^23^	2004	18	44(15)	Hebei	provincial	U&R	multi-stage stratified cluster random	20716	GHQ-12	SCID-I/P	DSM-IV	doctor and nurse of psychiatry department
Li N^24^	2004	18	–	Liaoning	provincial	U&R	multi-stage stratified cluster random	13358	CIDI1.0	CIDI	DSM-III-R	public health doctor
Ruan Y^34^	2005	15	39(15)	Yunnan	city	U&R	PPS	5033	CIDI2.1	CIDI2.1	DSM-IV	doctor and nurse of psychiatry department & medical student
Duan WD^31^	2005	18	32.49(11.16)	Guangdong	city	U	multi-stage stratified random	7134	CIDI3.1	CIDI3.1	ICD-10	psychiatrist & university student
Zhao ZH^38^	2006	15	47.3(17.2)	Guangdong	city	U&R	stratified cluster random	7418	CIDI3.0	SCID-I/P	DSM-IV	psychiatrist & trained researcher
Zhang XN^37^	2007	18	–	Liaoning	city	U&R	stratified random	5059	CIDI3.0	CIDI3.0	DSM-IV	psychiatrist & psychologist & postgraduate majoring in epidemiology and statistics
Wei B^18^	2007	15	–	Guangxi	provincial	U&R	multi-stage stratified cluster random	18219	CIDI3.0	CIDI3.0	ICD-10	psychiatrist &psychologist & undergraduate majoring in clinic medicine
Yu JC^36^	2009	16	40.3(15.2)	Guangdong	city	U&R	stratified cluster	2707	CIDI3.0	CIDI3.0	DSM-IV	doctor and nurse of psychiatry department & medical student
Fang X^21^	2009	15	41.3(16.6)	Fujian	provincial	U&R	multi-stage stratified cluster random	9986	GHQ-12	SCID-I/P	DSM-IV	doctor and nurse of psychiatry department & medical student
Chen HM^29^	2010	18	45(16)	Hebei	city	U&R	multi-stage stratified cluster	2360	GHQ-12	SCID-I/P	DSM-IV	doctor and nurse of psychiatry department & medical student
Chen XL^30^	2010	16	46.7(13.2)	Shannxi	city	U&R	PPS	2447	CIDI-3.0	CIDI-3.0	DSM-IV	trained college student
Liu ZR^19^	2010	16	53(17)	Beijing	city	U&R	multi-stage stratified sampling	2469	CIDI3.0	CIDI3.0	DSM-IV/ICD-10	trained researcher
Ma X^28^	2010	18	–	Beijing	provincial	U&R	multi-stage stratified cluster random	16032	–	SCID-I/P	DSM-IV	trained researcher
Wang WQ^35^	2010	18	43(16)	Fujian	city	U&R	multi-stage stratified cluster	10764	GHQ-12	SCID-I/P	DSM-IV	doctor and nurse of psychiatry department & medical student
Liu J^32^	2011	15	44.37(16.25)	Henan	city	U&R	multi-stage stratified cluster random	29636	Handbook	SCID-I/P	DSM-IV	non-psychiatric doctor & psychiatrist
Lu DY^33^	2011	15	44.65(14.25)	Guangdong	city	U&R	multi-stage stratified cluster random	2373	–	SCID-I/P	DSM-IV	trained researcher
Li T^25^	2012	18	43.98(15.44)	Ningxia	provincial	R	multi-stage stratified cluster random	4156	CIDI-CAPI	CIDI	ICD-10	postgraduate & undergraduate majoring on preventive medicine

Note: U: Urban; R: Rural; U&R: Urban & Rural; SD: Standard deviation. GHQ-12: General Health Questionnaire; CIDI: Composite International Diagnostic Interview; CAPI: Computer Assisted Personal Interviewing; SCID-I/P: Structured Clinical Interview for DSM-IV-TR Axis I Disorders-Patient Edition; Handbook: Mental disease epidemiology survey handbook; ICD-10: International Classification of Diseases, The 10th version. DSM-IV: Diagnostic and Statistical Manual of Mental Disorders, the Fourth vision. DSM-III-R: Diagnostic and Statistical Manual of Mental Disorders, the Third revised vision. PPS: Probability Proportional to Size.

**Table 2:** The prevalences of ADs and the differences in gender (males/females) and location (urban/rural).

Diseases	Items	current prevlence					lifetime prevalence				
		P*(%)	95% CI_1_	model	OR	95% CI_2_	P*(%)	95% CI_1_	Model	OR	95% CI_2_
Anxiety disorders	overall	24.47	17.97–30.98	–	–	–	41.12	31.09–51.15	–	–	–
	males	15.37	8.31–22.43	random	0.57	0.44–0.75	28.46	8.63–48.29	random	0.56	0.43–0.73
	females	25.74	11.87–39.61				53.69	16.74–90.65			
	urban	16.99	3.40–30.58	random	1.18	0.76–1.84	37.97	10.97–64.97	random	0.97	0.62–1.32
	rural	17.68	6.84–28.51				36.83	−0.32–73.99			
Generalized anxiety disorder	overall	5.17	3.72–6.63	–	–	–	4.66	3.17–6.14	–	–	–
	males	2.97	1.83–4.12	fixed	0.44	0.34–0.56	0.43	0.13–0.72	fixed	0.49	0.38–0.65
	females	6.32	3.45–9.19				5.63	2.59–8.66			
	urban	4.56	3.45–5.66	random	0.94	0.61–1.45	4.57	2.09–7.06	fixed	1.07	0.62–1.51
	rural	5.51	2.73–8.29				4.22	1.76–6.68			
Non-specific anxiety disorder	overall	8.30	4.49–12.10	–	–	–	6.89	0.43–13.35	–	–	–
	males	4.01	1.98–6.05	fixed	0.51	0.38–0.67	–	–	–	–	–
	females	7.65	2.96–12.34				–	–			
	urban	7.60	2.19–13.01	random	1.61	0.80–3.24	–	–	–	–	–
	rural	4.66	1.94–7.37				–	–			
Panic disorder	overall	1.08	0.74–1.43	–	–	–	3.44	2.46–4.41	–	–	–
	males	1.16	0.49–1.84	fixed	0.50	0.32–0.77	2.30	1.07–3.54	fixed	0.49	0.33–0.72
	females	2.01	0.67–3.34				4.53	2.01–7.05			
	urban	1.10	0.49–1.72	fixed	0.64	0.35–1.18	3.18	1.17–5.19	fixed	0.82	0.56–1.21
	rural	1.97	0.51–3.43				3.19	1.20–5.18			
Social phobia	overall	0.70	0.48–0.92	–	–	–	4.11	3.24–4.99	–	–	–
	males	1.94	0.83–3.05	fixed	0.82	0.53–1.27	5.68	2.26–9.10	fixed	0.77	0.62–0.96
	females	2.21	1.15–3.28				7.63	2.28–12.98			
	urban	1.56	0.59–2.52	fixed	1.04	0.50–2.16	4.18	0.60–7.77	fixed	0.68	0.51–0.90
	rural	1.52	1.03–2.01				5.70	0.33–11.07			
Agoraphobia	overall	0.19	0.10–0.28	–	–	–	2.15	1.56–2.74	–	–	–
	males	0.00	−0.01−0.01	fixed	0.17	0.04–0.76	0.21	−0.05–0.45	fixed	0.34	0.28–0.42
	females	0.77	0.03–1.52				11.09	3.41–18.76			
	urban	0.41	−0.16–0.98	fixed	0.58	0.12–2.30	5.23	0.71–9.75	random	0.52	0.17–1.60
	rural	0.71	0.18–1.24				10.43	–1.06–21.92			
Specific phobia	overall	0.63	0.49–0.77	–	–	–	19.61	15.18–24.04	–	–	–
	males	3.37	1.78–4.95	fixed	0.43	0.35–0.54	17.91	8.48–27.33	fixed	0.47	0.40–0.56
	females	9.39	5.23–13.55				37.14	19.19–55.09			
	urban	3.25	1.07–5.44	random	1.05	0.21–5.35	25.84	2.13–49.56	random	0.96	0.37–2.48
	rural	3.58	0.56–6.59				23.60	4.88–42.32			
Post-traumatic stress disorder	overall	0.49	0.35–0.63	–	–	–	1.83	1.23–2.43	–	–	–
	males	2.42	1.69–3.14	random	0.56	0.32–1.00	0.07	−0.10–0.23	random	0.89	0.12–6.74
	females	4.26	2.88–5.64				0.01	−0.09–0.11			
	urban	2.97	−0.03–5.96	random	0.86	0.26–2.81	0.01	−0.08–0.10	fixed	0.39	0.20–0.76
	rural	3.34	2.66–4.01				1.98	−13.62–17.57			
Obsessive compulsive disorder	overall	0.90	0.58–1.22	–	–	–	3.17	2.04–4.31	–	–	–
	males	1.11	0.08–2.15	fixed	0.87	0.51–1.51	3.55	1.01–6.08	fixed	0.54	0.36–0.82
	females	1.72	−0.10–3.54				8.71	3.64–13.79			
	urban	1.41	−0.51–3.32	fixed	2.32	1.08–4.99	2.77	0.37–5.18	fixed	0.68	0.39–1.18
	rural	0.69	−0.31–1.69				4.19	1.20–7.19			

P: prevalence; OR: odds ratio; CI: confidence interval.
