# Supplementary material for: Meta-analysis of the prevalence of anxiety disorders in mainland China from 2000 to 2015
Source: Sci Rep. 2016 Jun 16;6:28033. doi: 10.1038/srep28033 (PMC4910078; doi:10.1038/srep28033)
Supplement: Supplementary Information [file srep28033-s1.pdf]

Meta-analysis of the prevalence of anxiety disorders in mainland China from 2000 to 2015

Xiaojing Guo[1*], Zhen Meng[2*], Guifeng Huang[1*], Jingyuan Fan[2], Wenwen Zhou[2], Weijun Ling[1], Juan Jiang[1], Jianxiong Long[1§], Li Su[1§]

[1] School of Public Health of Guangxi Medical University, Nanning, Guangxi, China.

[2] Pre-Clinical Faculty of Guangxi Medical University, Nanning, Guangxi, China.

[*]First co-authors

[§]Corresponding author

Corresponding to:

Jianxiong Long, School of Public Health of Guangxi Medical University, 22 Shuangyong Road, Nanning, Guangxi ,China; Tel: +86771 5602912, Fax: +86771 5350823, E-mail: longjx12345@163.com.

Li Su, School of Public Health of Guangxi Medical University, 22 Shuangyong Road, Nanning, Guangxi ,China; Tel: +86771 5602912, Fax: +86771 5350823, E-mail: suli2018@hotmail.com.

Legends of Supplementary Information

Fig S1: Gender difference in the current prevalence of ADs and their subtypes

a. Gender difference in current prevalence of anxiety disorders

b. Gender difference in current prevalence of agoraphobia

c. Gender difference in current prevalence of generalized anxiety disorder

d. Gender difference in current prevalence of non-specific anxiety disorder

e. Gender difference in current prevalence of obsessive-compulsive disorder

f. Gender difference in current prevalence of panic disorder

g. Gender difference in current prevalence of post-traumatic stress disorder

h. Gender difference in current prevalence of social phobia

i. Gender difference in current prevalence of specific phobia

Fig S2: Gender difference in the lifetime prevalence of ADs and their subtypes

a. Gender difference in lifetime prevalence of anxiety disorders

b. Gender difference in lifetime prevalence of agoraphobia

c. Gender difference in lifetime prevalence of generalized anxiety disorder

d. Gender difference in lifetime prevalence of obsessive-compulsive disorder

e. Gender difference in lifetime prevalence of panic disorder

f. Gender difference in lifetime prevalence of post-traumatic stress disorder

g. Gender difference in lifetime prevalence of social phobia

h. Gender difference in lifetime prevalence of specific phobia

Fig S3: Location difference in the current prevalence of ADs and their subtypes

a. Location difference in current prevalence of anxiety disorders

b. Location difference in current prevalence of agoraphobia

c. Location difference in current prevalence of generalized anxiety disorder

d. Location difference in current prevalence of non-specific anxiety disorder

e. Location difference in current prevalence of obsessive-compulsive

disorder

f. Location difference in current prevalence of panic disorder

g. Location difference in current prevalence of post-traumatic stress disorder

h. Location difference in current prevalence of social phobia

i. Location difference in current prevalence of specific phobia

Fig S4: Location difference in the lifetime prevalence of ADs and their subtypes

a. Location difference in lifetime prevalence of anxiety disorders

b. Location difference in lifetime prevalence of agoraphobia

c. Location difference in lifetime prevalence of generalized anxiety disorder

d. Location difference in lifetime prevalence of obsessive-compulsive disorder

e. Location difference in lifetime prevalence of panic disorder

f. Location difference in lifetime prevalence of post-traumatic stress disorder

g. Location difference in lifetime prevalence of social phobia

h. Location difference in lifetime prevalence of specific phobia

Figure S5: The geographical and territorial structure of mainland China

Table S1: Studies characteristics with the extracted prevalence of ADs in this meta-analysis

Table S2: Literature quality score scale for accessing risk of bias

Table S3: Meta-regression for prevalence of Ads

Table S4: The results of sensitivity analysis **a.** Results of sensitivity analysis: the results of sensitivity analysis in ADs and its subtypes **b.** Results of sensitivity analysis: the characteristics of the studies those influenced the pooled results

Table S5: Heterogeneity and publication bias of ADs

Data S1: The excluded studies in this meta-analysis

Supplementary Information

Study or Subgroup	males Events	males Total	females Events	females Total	Weight	Odds Ratio M-H, Random, 95% CI
Chen HM 2012	27	1167	33	1193	16.9%	0.83 [0.50, 1.39]
Liu J 2012	91	15042	154	14594	31.9%	0.57 [0.44, 0.74]
Liu ZR 2013	24	967	54	1502	18.0%	0.68 [0.42, 1.11]
M R Phillips 2009	95	7176	225	7463	33.2%	0.43 [0.34, 0.55]
**Total (95% CI)**		**24352**		**24752**	**100.0%**	**0.57 [0.44, 0.75]**
Total events	237		466			

Heterogeneity: Tau² = 0.04; Chi² = 7.05, df = 3 (P = 0.07); I² = 57%
Test for overall effect: Z = 4.11 (P < 0.0001)

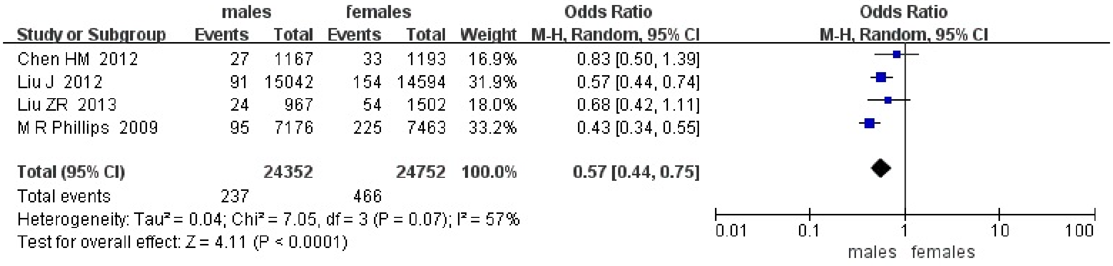

Fig S1a. Gender difference in current prevalence of anxiety disorders

Study or Subgroup	males Events	males Total	females Events	females Total	Weight	Odds Ratio M-H, Fixed, 95% CI
Duan WD 2011	1	3615	1	3519	8.2%	0.97 [0.06, 15.57]
Liu ZR 2013	0	967	2	1502	15.9%	0.31 [0.01, 6.47]
M R Phillips 2009	0	7176	9	7463	75.8%	0.05 [0.00, 0.94]
**Total (95% CI)**		**11758**		**12484**	**100.0%**	**0.17 [0.04, 0.76]**
Total events	1		12			

Heterogeneity: Chi² = 2.28, df = 2 (P = 0.32); I² = 12%
Test for overall effect: Z = 2.32 (P = 0.02)

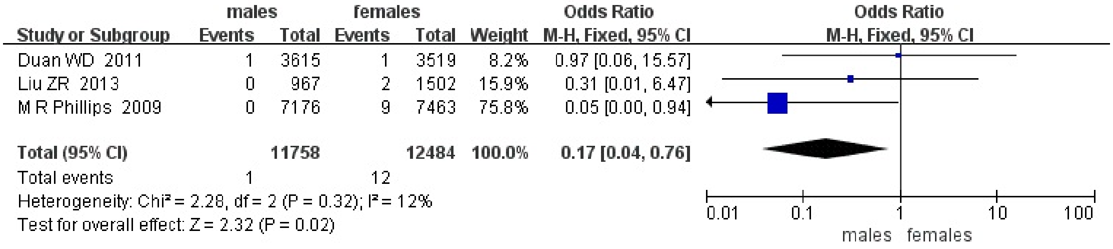

Fig S1b. Gender difference in current prevalence of agoraphobia.

Study or Subgroup	males Events	males Total	females Events	females Total	Weight	Odds Ratio M-H, Fixed, 95% CI
Fang X 2011	24	5079	59	4907	29.7%	0.39 [0.24, 0.63]
Li KQ 2007	39	10343	88	10373	43.6%	0.44 [0.30, 0.65]
Liu ZR 2013	3	967	3	1502	1.2%	1.55 [0.31, 7.72]
M R Phillips 2009	16	7176	33	7463	16.1%	0.50 [0.28, 0.91]
Zhao ZH 2009	5	3226	22	4192	9.5%	0.29 [0.11, 0.78]
**Total (95% CI)**		**26791**		**28437**	**100.0%**	**0.44 [0.34, 0.56]**
Total events	87		205			

Heterogeneity: Chi² = 3.48, df = 4 (P = 0.48); I² = 0%
Test for overall effect: Z = 6.48 (P < 0.00001)

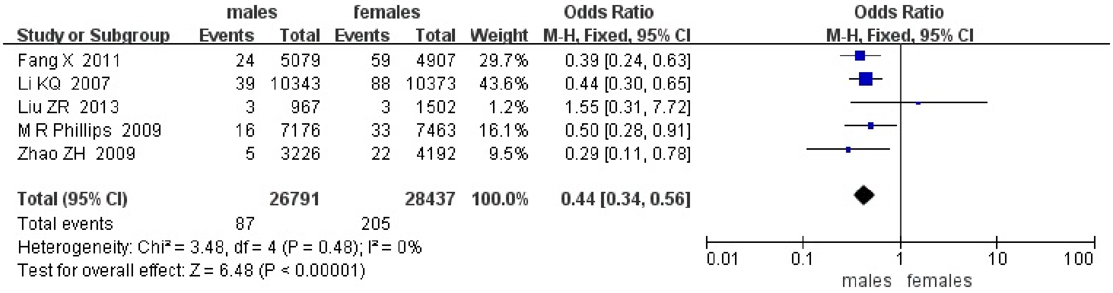

Fig S1c. Gender difference in current prevalence of generalized anxiety disorder.

Study or Subgroup	males Events	males Total	females Events	females Total	Weight	Odds Ratio M-H, Fixed, 95% CI
Fang X 2011	24	5079	35	4907	24.5%	0.66 [0.39, 1.11]
M R Phillips 2009	38	7176	90	7463	60.8%	0.44 [0.30, 0.64]
Wang WQ 2013	11	4957	23	5807	14.6%	0.56 [0.27, 1.15]
**Total (95% CI)**		**17212**		**18177**	**100.0%**	**0.51 [0.38, 0.67]**
Total events	73		148			

Heterogeneity: Chi² = 1.66, df = 2 (P = 0.44); I² = 0%
Test for overall effect: Z = 4.70 (P < 0.00001)

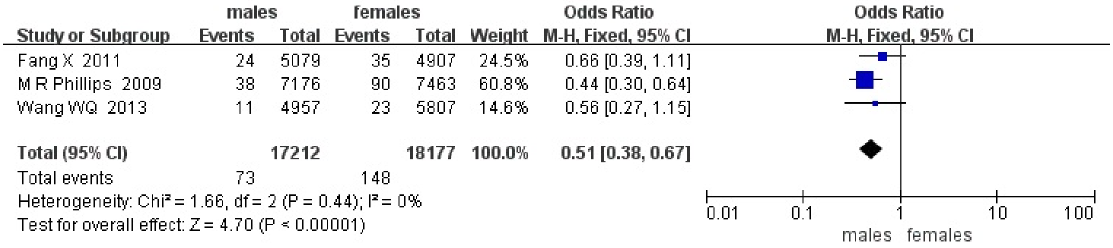

Fig S1d. Gender difference in current prevalence of non-specific anxiety disorder.

Study or Subgroup	males Events	males Total	females Events	females Total	Weight	Odds Ratio M-H, Fixed, 95% CI
Liu ZR 2013	6	967	14	1502	39.2%	0.66 [0.25, 1.73]
M R Phillips 2009	4	7176	1	7463	3.5%	4.16 [0.47, 37.24]
Wang WQ 2013	13	10343	16	10373	57.3%	0.81 [0.39, 1.69]
**Total (95% CI)**		**18486**		**19338**	**100.0%**	**0.87 [0.51, 1.51]**
Total events	23		31			
Heterogeneity: Chi² = 2.30, df = 2 (P = 0.32); I² = 13%						
Test for overall effect: Z = 0.49 (P = 0.63)						

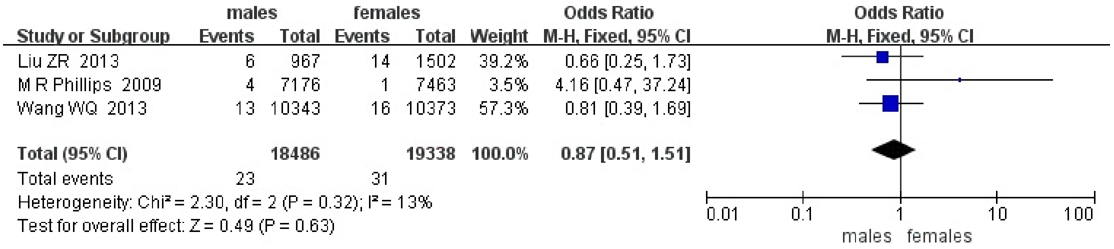

Fig S1e. Gender difference in current prevalence of obsessive-compulsive disorder.

Study or Subgroup	males Events	males Total	females Events	females Total	Weight	Odds Ratio M-H, Fixed, 95% CI
Fang X 2011	9	5079	12	4907	20.1%	0.72 [0.30, 1.72]
Li KQ 2007	16	10343	39	10373	64.1%	0.41 [0.23, 0.74]
Liu ZR 2013	1	967	1	1502	1.3%	1.55 [0.10, 24.87]
M R Phillips 2009	4	7176	9	7463	14.5%	0.46 [0.14, 1.50]
**Total (95% CI)**		**23565**		**24245**	**100.0%**	**0.50 [0.32, 0.77]**
Total events	30		61			
Heterogeneity: Chi² = 1.81, df = 3 (P = 0.61); I² = 0%						
Test for overall effect: Z = 3.14 (P = 0.002)						

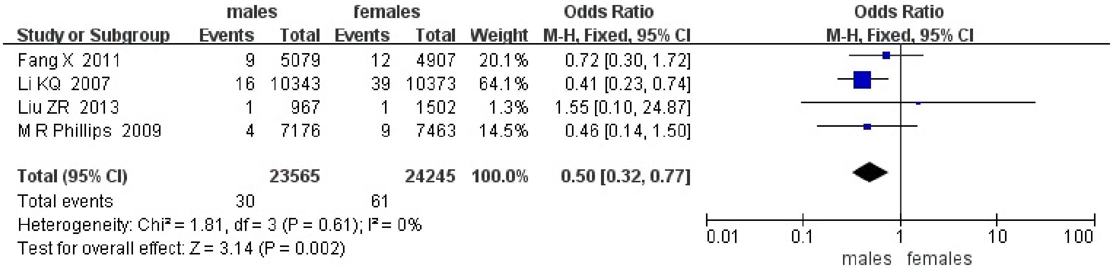

Fig S1f. Gender difference in current prevalence of panic disorder.

Study or Subgroup	males Events	males Total	females Events	females Total	Weight	Odds Ratio M-H, Random, 95% CI
Li KQ 2007	28	10343	38	10373	54.4%	0.74 [0.45, 1.20]
M R Phillips 2009	15	7176	38	7463	45.6%	0.41 [0.22, 0.74]
**Total (95% CI)**		**17519**		**17836**	**100.0%**	**0.56 [0.32, 1.00]**
Total events	43		76			
Heterogeneity: Tau² = 0.10; Chi² = 2.24, df = 1 (P = 0.13); I² = 55%						
Test for overall effect: Z = 1.95 (P = 0.05)						

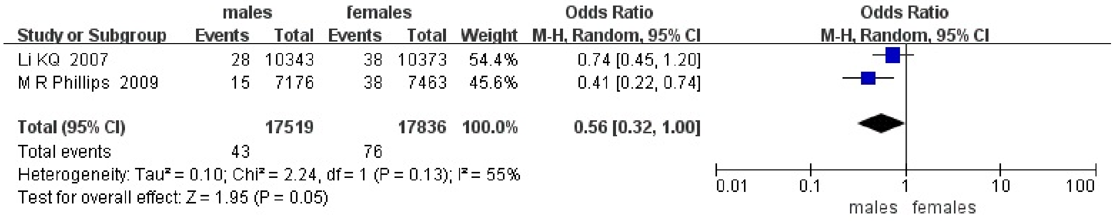

Fig S1g. Gender difference in current prevalence of post-traumatic stress disorder.

Study or Subgroup	males Events	males Total	females Events	females Total	Weight	Odds Ratio M-H, Fixed, 95% CI
Duan WD 2011	12	3615	12	3519	27.2%	0.97 [0.44, 2.17]
Fang X 2011	9	5079	6	4907	13.7%	1.45 [0.52, 4.08]
Li KQ 2007	11	10343	21	10373	46.9%	0.52 [0.25, 1.09]
Liu ZR 2013	4	967	7	1502	12.2%	0.89 [0.26, 3.04]
**Total (95% CI)**		**20004**		**20301**	**100.0%**	**0.82 [0.53, 1.27]**
Total events	36		46			
Heterogeneity: Chi² = 2.80, df = 3 (P = 0.42); I² = 0%						
Test for overall effect: Z = 0.90 (P = 0.37)						

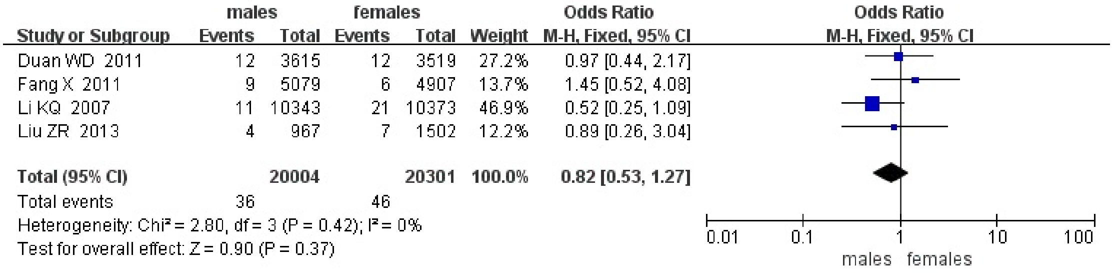

Fig S1h. Gender difference in current prevalence of social phobia.

Study or Subgroup	males Events	males Total	females Events	females Total	Weight	Odds Ratio M-H, Fixed, 95% CI
Duan WD 2011	19	3615	57	3519	22.2%	0.32 [0.19, 0.54]
Fang X 2011	14	5079	30	4907	11.7%	0.45 [0.24, 0.85]
Li KQ 2007	46	10343	93	10373	35.6%	0.49 [0.35, 0.70]
Liu ZR 2013	12	967	37	1502	11.0%	0.50 [0.26, 0.96]
M R Phillips 2009	15	7176	44	7463	16.6%	0.35 [0.20, 0.64]
Wang WQ 2013	5	4957	8	5807	2.8%	0.73 [0.24, 2.24]
**Total (95% CI)**		**32137**		**33571**	**100.0%**	**0.43 [0.35, 0.54]**
Total events	111		269			
Heterogeneity: Chi² = 3.29, df = 5 (P = 0.66); I² = 0%						
Test for overall effect: Z = 7.36 (P < 0.00001)						

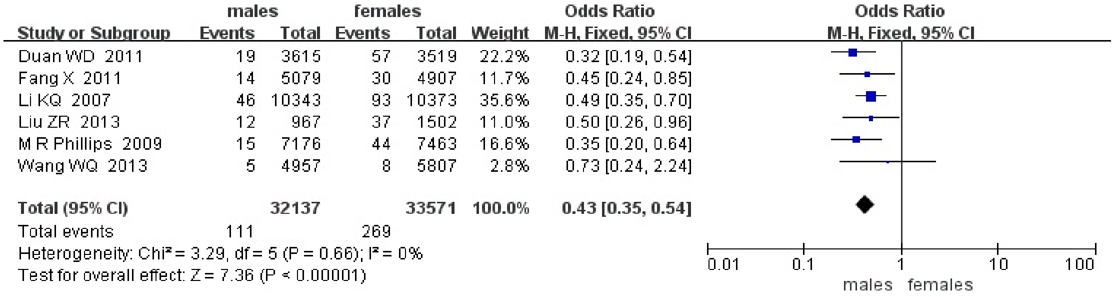

Fig S1i. Gender difference in current prevalence of specific phobia.

Study or Subgroup	Male Events	Male Total	Female Events	Female Total	Weight	Odds Ratio M-H, Random, 95% CI
Li N 2008	295	6610	668	6748	30.1%	0.43 [0.37, 0.49]
Liu ZR 2013	44	967	103	1502	20.5%	0.65 [0.45, 0.93]
Ruan Y 2010	100	2416	224	2617	25.9%	0.46 [0.36, 0.59]
Wei B 2010	13	9196	10	9023	8.0%	1.28 [0.56, 2.91]
Zhang XN 2010	26	2314	42	2745	15.6%	0.73 [0.45, 1.20]
**Total (95% CI)**		**21503**		**22635**	**100.0%**	**0.56 [0.43, 0.73]**
Total events	478		1047			

Heterogeneity: $Tau^2 = 0.06$; $Chi^2 = 13.67$, df = 4 (P = 0.008); $I^2 = 71\%$
Test for overall effect: Z = 4.22 (P < 0.0001)

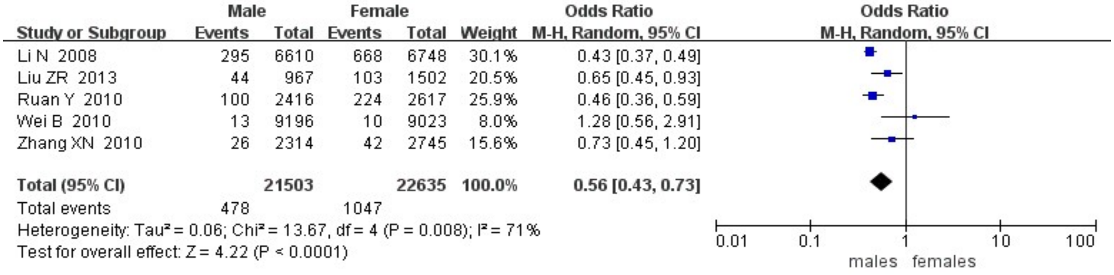

Fig S2a. Gender difference in lifetime prevalence of anxiety disorders

Study or Subgroup	Male Events	Male Total	Female Events	Female Total	Weight	Odds Ratio M-H, Fixed, 95% CI
Chen XL 2012	0	950	4	1497	1.0%	0.17 [0.01, 3.25]
Duan WD 2011	6	3615	12	3519	3.5%	0.49 [0.18, 1.30]
Li N 2008	106	6610	300	6748	83.4%	0.35 [0.28, 0.44]
Liu ZR 2013	0	967	3	1502	0.8%	0.22 [0.01, 4.29]
Ruan Y 2010	10	2416	40	2617	10.9%	0.27 [0.13, 0.54]
Yu JC 2010	0	1300	1	1407	0.4%	0.36 [0.01, 8.86]
**Total (95% CI)**		**15858**		**17290**	**100.0%**	**0.34 [0.28, 0.42]**
Total events	122		360			

Heterogeneity: $Chi^2 = 1.29$, df = 5 (P = 0.94); $I^2 = 0\%$
Test for overall effect: Z = 10.14 (P < 0.00001)

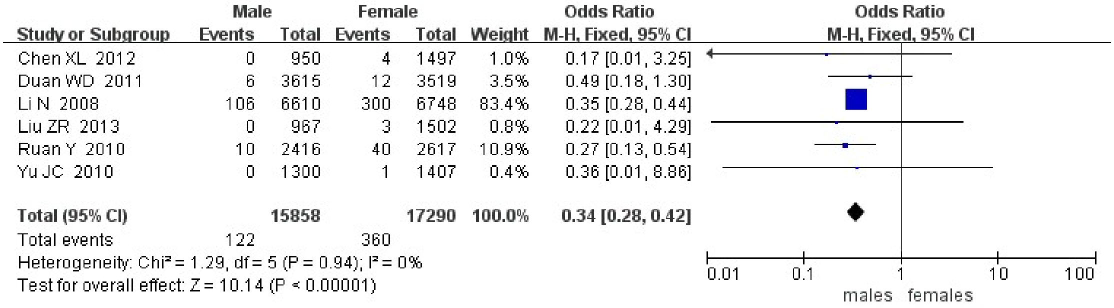

Fig S2b. Gender difference in lifetime prevalence of agoraphobia.

Study or Subgroup	Male Events	Male Total	Female Events	Female Total	Weight	Odds Ratio M-H, Fixed, 95% CI
Chen XL 2012	0	950	2	1497	1.2%	0.31 [0.02, 6.56]
Li N 2008	26	6610	67	6748	40.4%	0.39 [0.25, 0.62]
Liu ZR 2013	3	967	5	1502	2.4%	0.93 [0.22, 3.91]
Ma X 2009	18	2735	53	3191	29.7%	0.39 [0.23, 0.67]
Ruan Y 2010	17	2416	23	2617	13.4%	0.80 [0.43, 1.50]
Wei B 2010	5	9196	6	9023	3.7%	0.82 [0.25, 2.68]
Yu JC 2010	0	1300	3	1407	2.1%	0.15 [0.01, 2.99]
Zhang XN 2010	8	2314	13	2745	7.2%	0.73 [0.30, 1.76]
**Total (95% CI)**		**26488**		**28730**	**100.0%**	**0.49 [0.38, 0.65]**
Total events	77		172			

Heterogeneity: $Chi^2 = 6.78$, df = 7 (P = 0.45); $I^2 = 0\%$
Test for overall effect: Z = 5.14 (P < 0.00001)

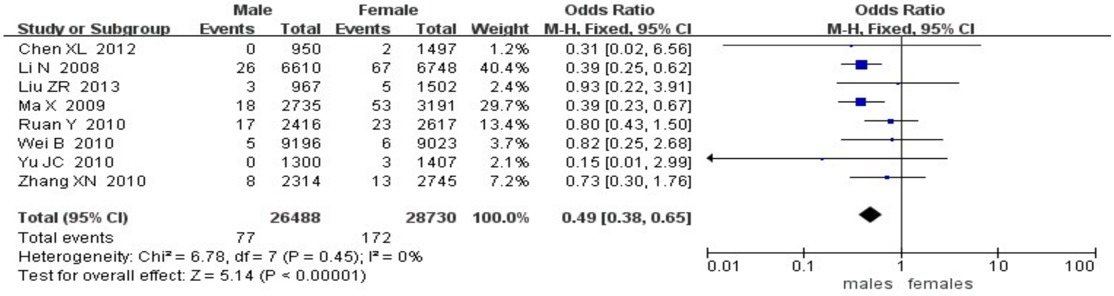

Fig S2c. Gender difference in lifetime prevalence of generalized anxiety disorder.

Study or Subgroup	Male Events	Male Total	Female Events	Female Total	Weight	Odds Ratio M-H, Fixed, 95% CI
Chen XL 2012	5	950	20	1497	23.0%	0.39 [0.15, 1.04]
Liu ZR 2013	16	967	46	1502	52.8%	0.53 [0.30, 0.95]
Ruan Y 2010	3	2416	7	2617	10.0%	0.46 [0.12, 1.79]
Yu JC 2010	6	1300	8	1407	11.4%	0.81 [0.28, 2.34]
Zhang XN 2010	2	2314	2	2745	2.7%	1.19 [0.17, 8.43]
**Total (95% CI)**		**7947**		**9768**	**100.0%**	**0.54 [0.36, 0.82]**
Total events	32		83			

Heterogeneity: $Chi^2 = 1.65$, df = 4 (P = 0.80); $I^2 = 0\%$
Test for overall effect: Z = 2.90 (P = 0.004)

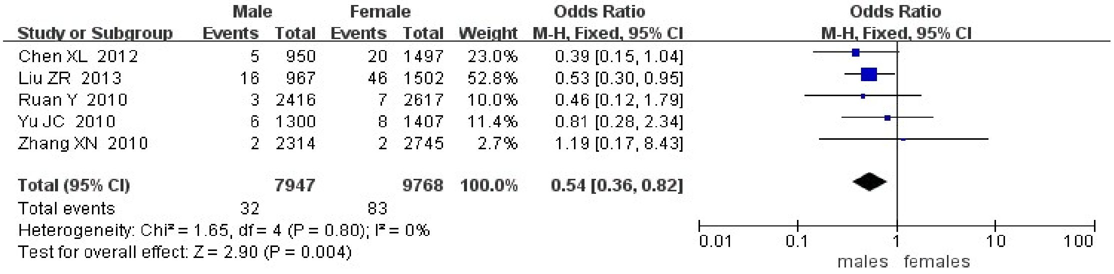

Fig S2d. Gender difference in lifetime prevalence of obsessive-compulsive disorder.

Study or Subgroup	Male Events	Male Total	Female Events	Female Total	Weight	Odds Ratio M-H, Fixed, 95% CI
Chen XL 2012	2	950	4	1497	3.9%	0.79 [0.14, 4.31]
Li N 2008	10	6610	36	6748	44.3%	0.28 [0.14, 0.57]
Liu ZR 2013	3	967	11	1502	10.7%	0.42 [0.12, 1.52]
Ruan Y 2010	3	2416	3	2617	3.6%	1.08 [0.22, 5.37]
Yu JC 2010	3	1300	3	1407	3.6%	1.08 [0.22, 5.37]
Zhang XN 2010	16	2314	30	2745	34.0%	0.63 [0.34, 1.16]
**Total (95% CI)**		**14557**		**16516**	**100.0%**	**0.49 [0.33, 0.72]**
Total events	37		87			

Heterogeneity: $Chi^2 = 5.25$, $df = 5$ ($P = 0.39$); $I^2 = 5\%$
Test for overall effect: $Z = 3.60$ ($P = 0.0003$)

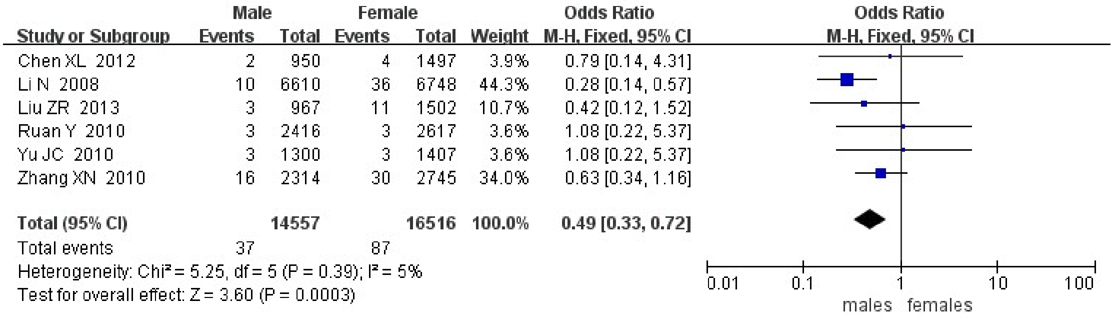

Fig S2e. Gender difference in lifetime prevalence of panic disorder.

Study or Subgroup	Male Events	Male Total	Female Events	Female Total	Weight	Odds Ratio M-H, Random, 95% CI
Chen XL 2012	0	950	0	1497		Not estimable
Ruan Y 2010	16	2416	17	2617	49.4%	1.02 [0.51, 2.02]
Wei B 2010	4	9196	0	9023	25.1%	8.83 [0.48, 164.11]
Yu JC 2010	0	1300	7	1407	25.6%	0.07 [0.00, 1.26]
**Total (95% CI)**		**13862**		**14544**	**100.0%**	**0.89 [0.12, 6.74]**
Total events	20		24			

Heterogeneity: $Tau^2 = 2.04$; $Chi^2 = 5.47$, $df = 2$ ($P = 0.06$); $I^2 = 63\%$
Test for overall effect: $Z = 0.11$ ($P = 0.91$)

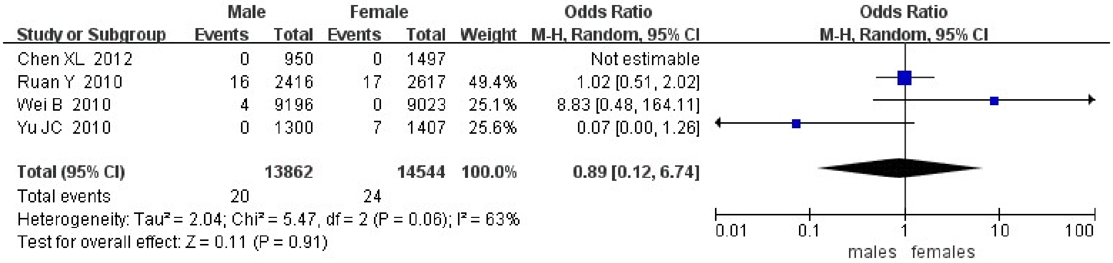

Fig S2f. Gender difference in lifetime prevalence of post-traumatic stress disorder.

Study or Subgroup	Male Events	Male Total	Female Events	Female Total	Weight	Odds Ratio M-H, Fixed, 95% CI
Chen XL 2012	4	950	9	1497	3.6%	0.70 [0.21, 2.28]
Duan WD 2011	58	3615	48	3519	25.0%	1.18 [0.80, 1.73]
Li N 2008	71	6610	109	6748	55.8%	0.66 [0.49, 0.89]
Liu ZR 2013	7	967	14	1502	5.7%	0.78 [0.31, 1.93]
Ruan Y 2010	3	2416	14	2617	7.0%	0.23 [0.07, 0.81]
Wei B 2010	1	9196	0	9023	0.3%	2.94 [0.12, 72.27]
Yu JC 2010	3	1300	5	1407	2.5%	0.65 [0.15, 2.72]
**Total (95% CI)**		**25054**		**26313**	**100.0%**	**0.77 [0.62, 0.96]**
Total events	147		199			

Heterogeneity: $Chi^2 = 9.99$, $df = 6$ ($P = 0.12$); $I^2 = 40\%$
Test for overall effect: $Z = 2.33$ ($P = 0.02$)

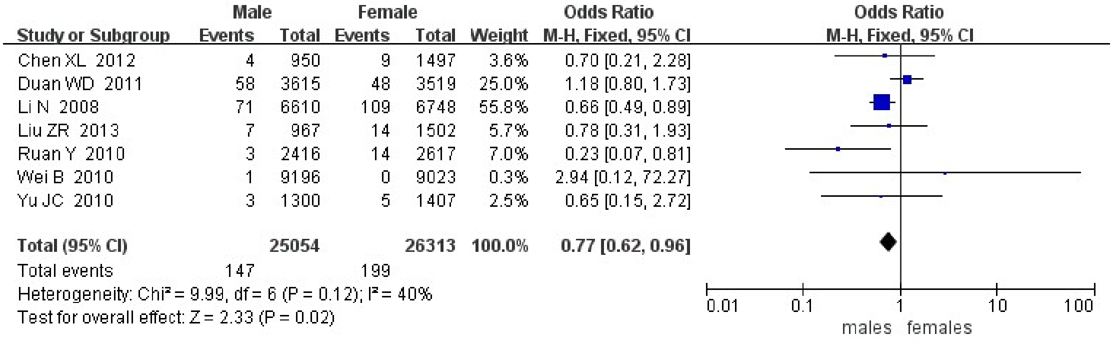

Fig S2g. Gender difference in lifetime prevalence of social phobia.

Study or Subgroup	Male Events	Male Total	Female Events	Female Total	Weight	Odds Ratio M-H, Fixed, 95% CI
Chen XL 2012	9	950	38	1497	7.1%	0.37 [0.18, 0.76]
Duan WD 2011	90	3615	167	3519	40.2%	0.51 [0.39, 0.67]
Liu ZR 2013	21	967	55	1502	10.3%	0.58 [0.35, 0.97]
Ruan Y 2010	68	2416	168	2617	38.2%	0.42 [0.32, 0.56]
Yu JC 2010	8	1300	18	1407	4.2%	0.48 [0.21, 1.10]
**Total (95% CI)**		**9248**		**10542**	**100.0%**	**0.47 [0.40, 0.56]**
Total events	196		446			

Heterogeneity: $Chi^2 = 2.08$, $df = 4$ ($P = 0.72$); $I^2 = 0\%$
Test for overall effect: $Z = 8.55$ ($P < 0.00001$)

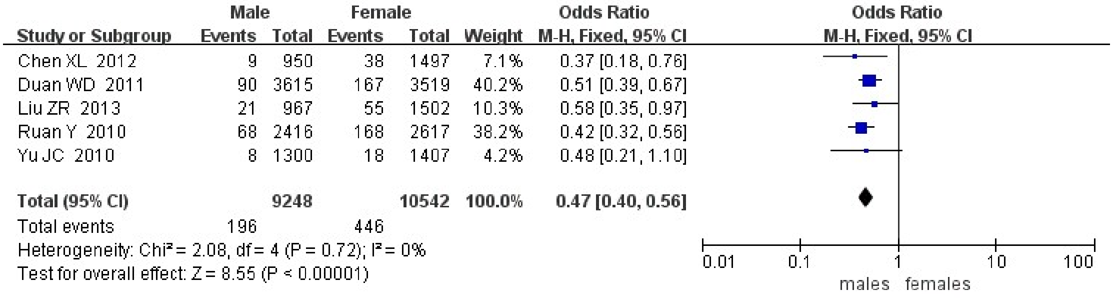

Fig S2h. Gender difference in lifetime prevalence of specific phobia.

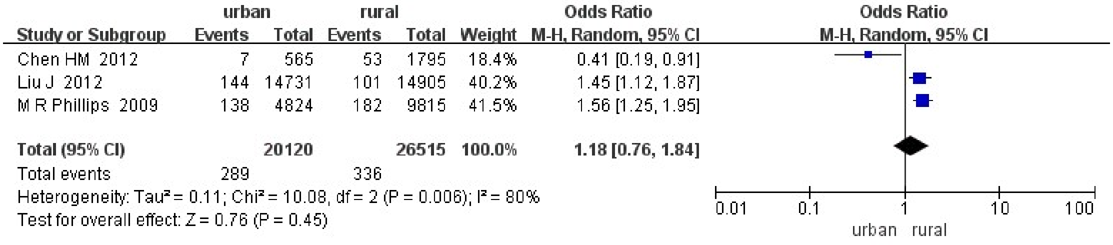

Fig S3a. Location difference in current prevalence of anxiety disorders

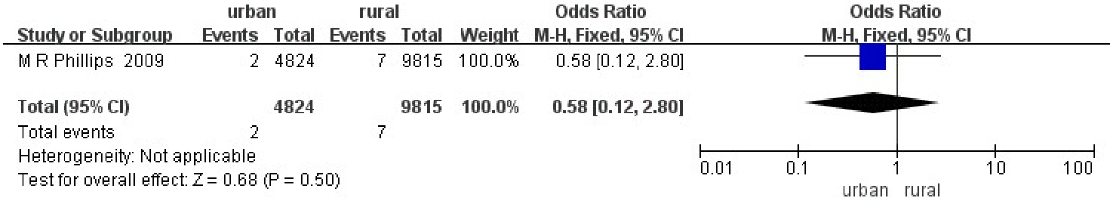

Fig S3b. Location difference in current prevalence of agoraphobia.

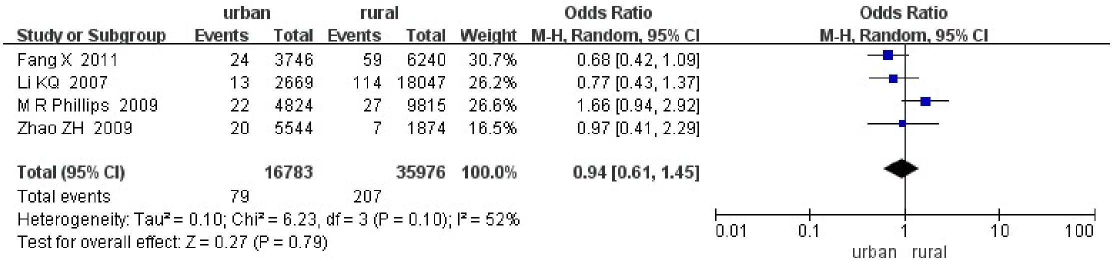

Fig S3c. Location difference in current prevalence of generalized anxiety disorder.

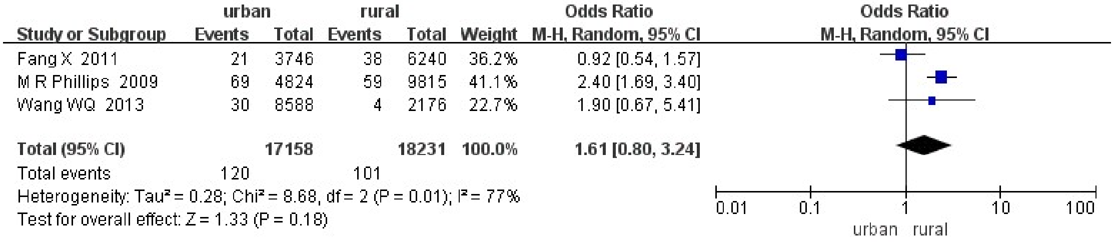

Fig S3d. Location difference in current prevalence of non-specific anxiety disorder.

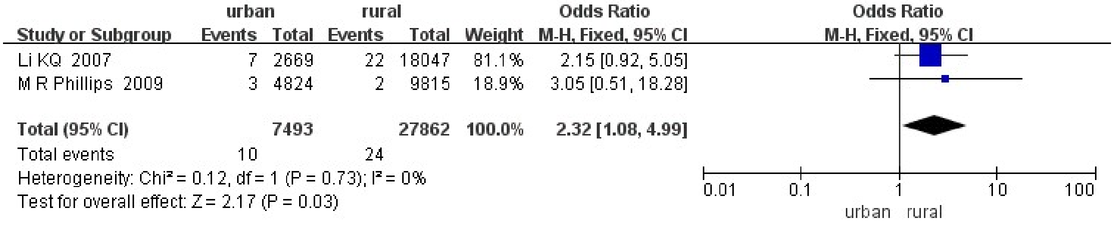

Fig S3e. Location difference in current prevalence of obsessive-compulsive disorder.

Study or Subgroup	urban Events	urban Total	rural Events	rural Total	Weight	Odds Ratio M-H, Fixed, 95% CI
Fang X 2011	7	3746	14	6240	35.7%	0.83 [0.34, 2.06]
Li KQ 2007	2	2669	53	18047	46.4%	0.25 [0.06, 1.05]
M R Phillips 2009	5	4824	8	9815	17.9%	1.27 [0.42, 3.89]
**Total (95% CI)**		**11239**		**34102**	**100.0%**	**0.64 [0.35, 1.18]**
Total events	14		75			

Heterogeneity: Chi² = 3.39, df = 2 (P = 0.18); I² = 41%
Test for overall effect: Z = 1.43 (P = 0.15)

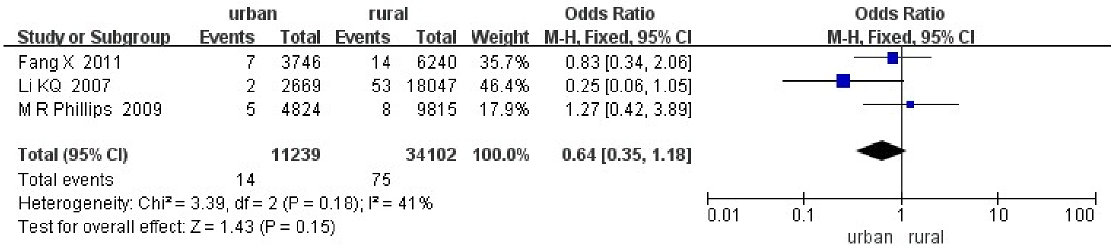

Fig S3f. Location difference in current prevalence of panic disorder.

Study or Subgroup	urban Events	urban Total	rural Events	rural Total	Weight	Odds Ratio M-H, Random, 95% CI
Li KQ 2007	4	2669	62	18047	43.7%	0.44 [0.16, 1.20]
M R Phillips 2009	22	4824	31	9815	56.3%	1.45 [0.84, 2.50]
**Total (95% CI)**		**7493**		**27862**	**100.0%**	**0.86 [0.26, 2.81]**
Total events	26		93			

Heterogeneity: Tau² = 0.57; Chi² = 4.33, df = 1 (P = 0.04); I² = 77%
Test for overall effect: Z = 0.26 (P = 0.80)

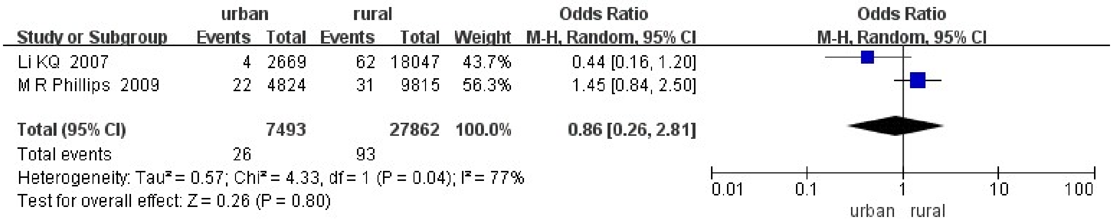

Fig S3g. Location difference in current prevalence of post-traumatic stress disorder.

Study or Subgroup	urban Events	urban Total	rural Events	rural Total	Weight	Odds Ratio M-H, Fixed, 95% CI
Fang X 2011	6	3746	9	6240	48.3%	1.11 [0.40, 3.12]
Li KQ 2007	4	2669	28	18047	51.7%	0.97 [0.34, 2.76]
**Total (95% CI)**		**6415**		**24287**	**100.0%**	**1.04 [0.50, 2.16]**
Total events	10		37			

Heterogeneity: Chi² = 0.03, df = 1 (P = 0.85); I² = 0%
Test for overall effect: Z = 0.09 (P = 0.92)

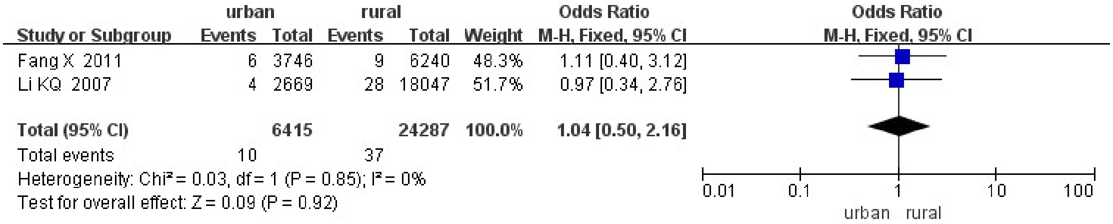

Fig S3h. Location difference in current prevalence of social phobia.

Study or Subgroup	urban Events	urban Total	rural Events	rural Total	Weight	Odds Ratio M-H, Random, 95% CI
Fang X 2011	36	3746	8	6240	26.0%	7.56 [3.51, 16.28]
Li KQ 2007	4	2669	135	18047	25.0%	0.20 [0.07, 0.54]
M R Phillips 2009	13	4824	46	9815	26.6%	0.57 [0.31, 1.06]
Wang WQ 2013	11	8588	2	2176	22.3%	1.39 [0.31, 6.29]
**Total (95% CI)**		**19827**		**36278**	**100.0%**	**1.05 [0.21, 5.35]**
Total events	64		191			

Heterogeneity: Tau² = 2.50; Chi² = 40.13, df = 3 (P < 0.00001); I² = 93%
Test for overall effect: Z = 0.06 (P = 0.95)

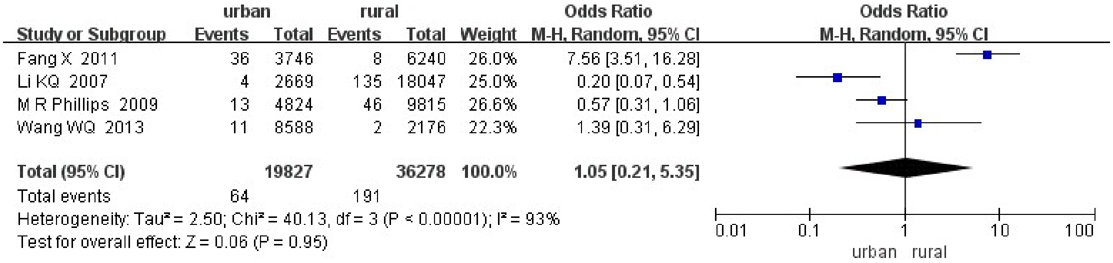

Fig S3i. Location difference in current prevalence of specific phobia.

Study or Subgroup	urban Events	urban Total	rural Events	rural Total	Weight	Odds Ratio M-H, Random, 95% CI
Li N 2008	345	5517	618	7841	31.6%	0.78 [0.68, 0.89]
Ruan Y 2010	189	2431	135	2602	30.0%	1.54 [1.23, 1.94]
Wei B 2010	8	7028	15	11191	14.8%	0.85 [0.36, 2.00]
Zhang XN 2010	39	3204	29	1855	23.6%	0.78 [0.48, 1.26]
**Total (95% CI)**		**18180**		**23489**	**100.0%**	**0.97 [0.62, 1.51]**
Total events	581		797			

Heterogeneity: Tau² = 0.16; Chi² = 25.77, df = 3 (P < 0.0001); I² = 88%
Test for overall effect: Z = 0.14 (P = 0.88)

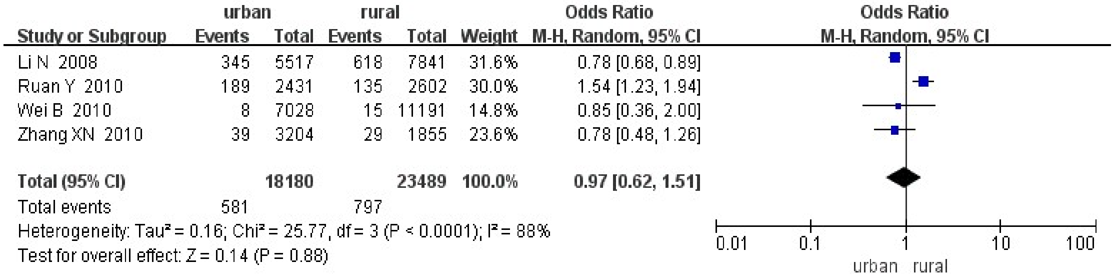

Fig S4a. Location difference in lifetime prevalence of anxiety disorders.

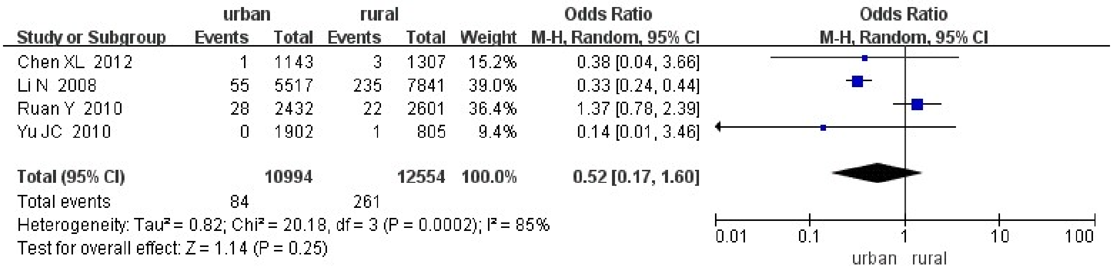

Fig S4b. Location difference in lifetime prevalence of agoraphobia.

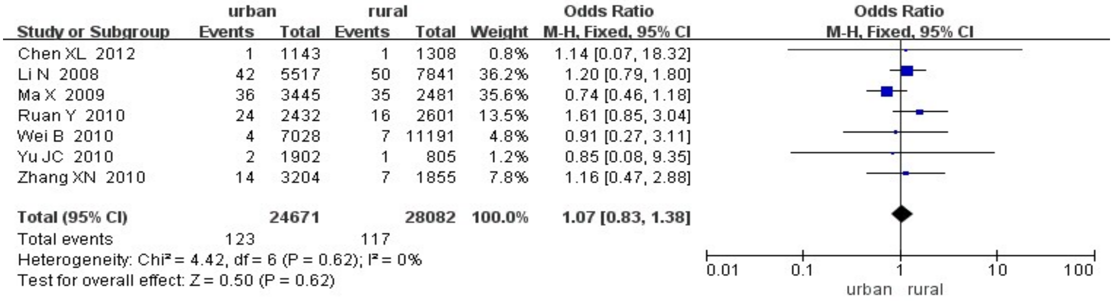

Fig S4c. Location difference in lifetime prevalence of generalized anxiety disorder.

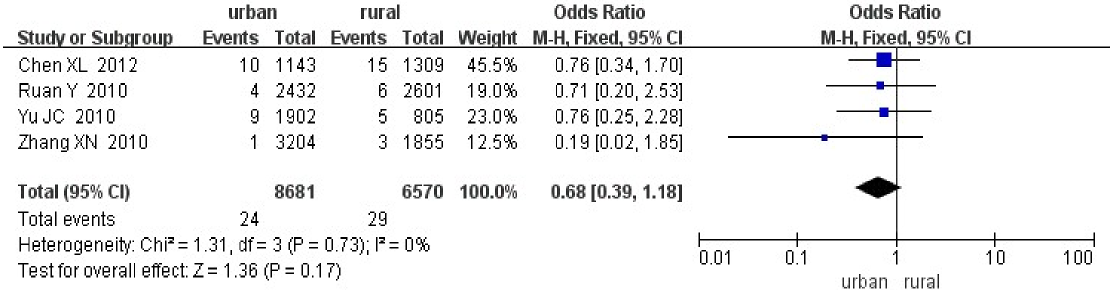

Fig S4d. Location difference in lifetime prevalence of obsessive-compulsive disorder.

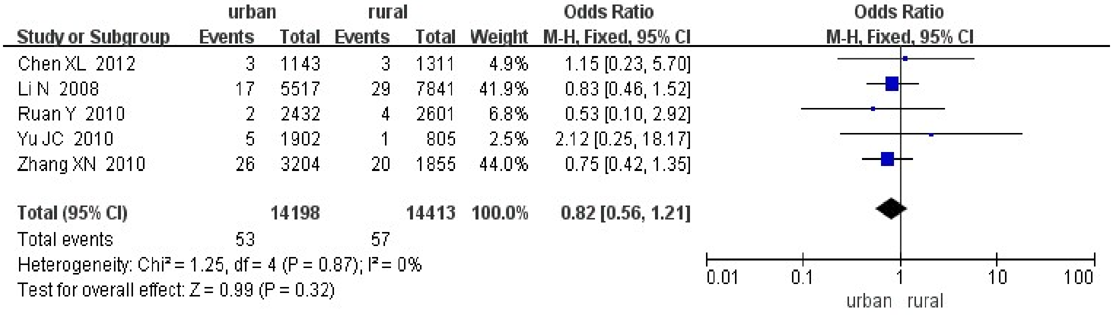

Fig S4e. Location difference in lifetime prevalence of panic disorder.

Study or Subgroup	urban Events	urban Total	rural Events	rural Total	Weight	Odds Ratio M-H, Fixed, 95% CI
Chen XL 2012	0	1143	0	1312		Not estimable
Ruan Y 2010	8	2432	25	2601	79.3%	0.34 [0.15, 0.76]
Wei B 2010	0	7028	4	11191	11.4%	0.18 [0.01, 3.29]
Yu JC 2010	5	1902	2	805	9.2%	1.06 [0.20, 5.47]
**Total (95% CI)**		**12505**		**15909**	**100.0%**	**0.39 [0.20, 0.76]**
Total events	13		31			

Heterogeneity: Chi² = 1.82, df = 2 (P = 0.40); I² = 0%
Test for overall effect: Z = 2.78 (P = 0.005)

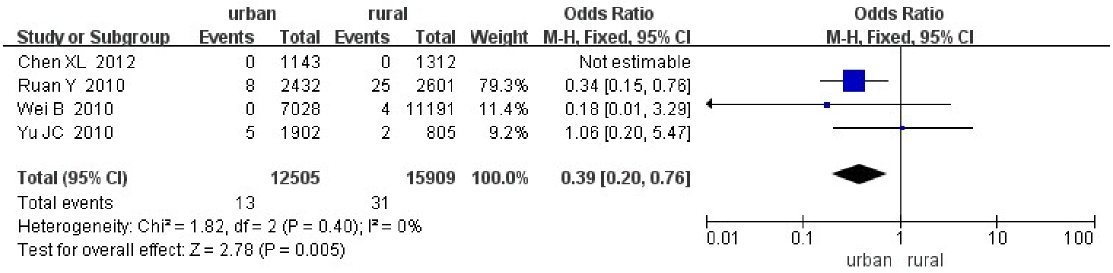

Fig S4f. Location difference in lifetime prevalence of post-traumatic stress disorder

Study or Subgroup	urban Events	urban Total	rural Events	rural Total	Weight	Odds Ratio M-H, Fixed, 95% CI
Chen XL 2012	6	1143	7	1305	5.3%	0.98 [0.33, 2.92]
Li N 2008	53	5517	127	7841	85.0%	0.59 [0.43, 0.81]
Ruan Y 2010	11	2432	6	2601	4.7%	1.97 [0.73, 5.32]
Wei B 2010	1	7028	0	11191	0.3%	4.78 [0.19, 117.29]
Yu JC 2010	4	1902	4	805	4.6%	0.42 [0.11, 1.69]
**Total (95% CI)**		**18022**		**23743**	**100.0%**	**0.68 [0.51, 0.90]**
Total events	75		144			

Heterogeneity: Chi² = 7.42, df = 4 (P = 0.12); I² = 46%
Test for overall effect: Z = 2.68 (P = 0.007)

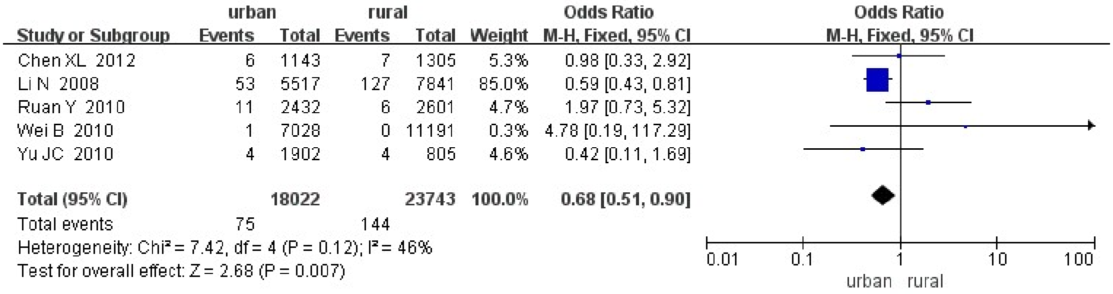

Fig S4g. Location difference in lifetime prevalence of social phobia.

Study or Subgroup	urban Events	urban Total	rural Events	rural Total	Weight	Odds Ratio M-H, Random, 95% CI
Chen XL 2012	12	1143	35	1306	33.0%	0.39 [0.20, 0.75]
Ruan Y 2010	140	2432	96	2601	38.2%	1.59 [1.22, 2.08]
Yu JC 2010	20	1902	6	805	28.7%	1.42 [0.57, 3.54]
**Total (95% CI)**		**5477**		**4712**	**100.0%**	**0.96 [0.37, 2.48]**
Total events	172		137			

Heterogeneity: Tau² = 0.59; Chi² = 15.36, df = 2 (P = 0.0005); I² = 87%
Test for overall effect: Z = 0.08 (P = 0.94)

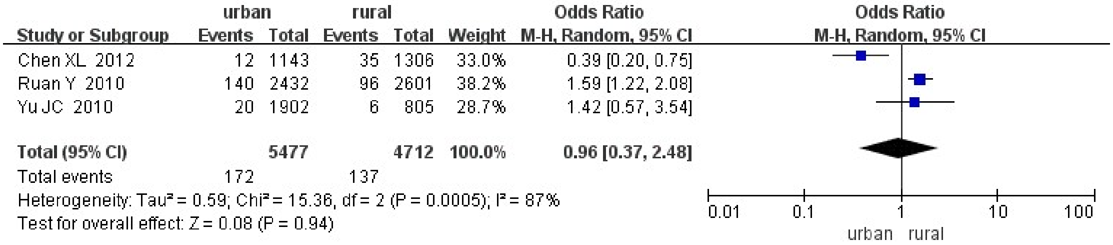

Fig S4h. Location difference in lifetime prevalence of specific phobia.

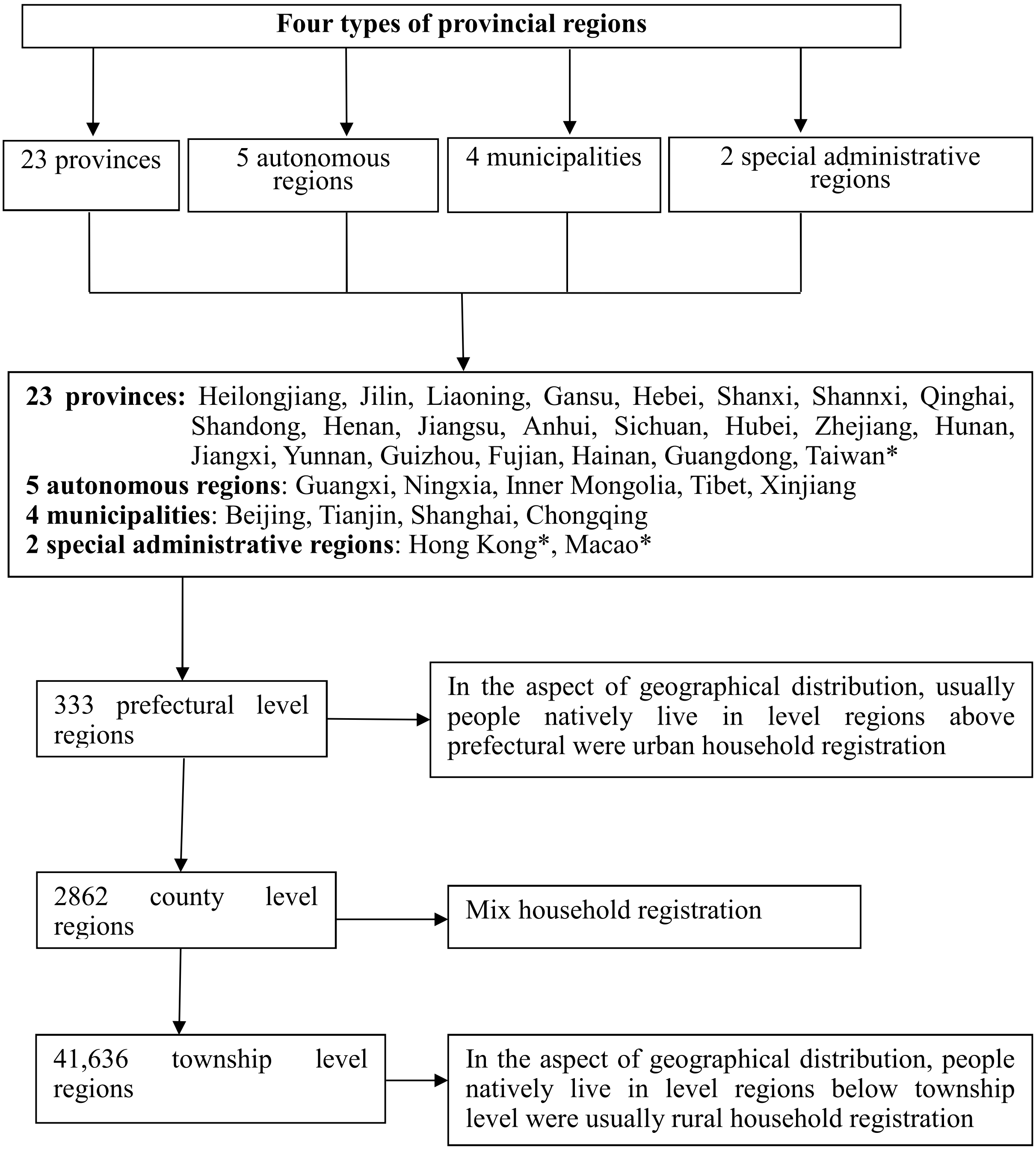

Figure S5. The geographical and territorial structure of mainland China

Administrative layer structure: Township level regions are directly affiliated to the corresponding county level regions; the county level regions are directly affiliated to the corresponding prefectural level regions; and the prefectural level regions are directly affiliated to the corresponding provincial region.

* The present study was conducted in mainland China (not including Hong Kong, Macao and Taiwan).

Table S1. Studies characteristics with the extracted prevalence of ADs in this meta-analysis

Subtypes	Study	Location	Year	Sample			Current/Life-time prevalence (‰)				
				overall	male/female	urban/rural	overall (n)	male (n)	female (n)	urban (n)	rural (n)
Anxiety disorders	M R Phillips 2009	Zhejiang	2001	14639	7176/7463	4824/9815	21.86/- (320/-)	13.24/- (95/-)	30.15/- (225/-)	28.61/- (138/-)	18.54/- (182/-)
		Qinghai	2002	11178	5652/5526	2632/8546	19.59/- (219/-)	-	-	-	-
		Shandong	2004	22718	10457/12261	5660/17059	30.77/- (699/-)	-	-	-	-
		Gansu	2005	10249	5517/4730	1645/8604	29.95/- (307/-)	-	-	-	-
		Shandong	2005	4776	2211/2565	2380/2396	39.15/- (187/-)	-	-	-	-
	Lee S 2007	Beijing/ Shanghai	2001	1628	-	-	-/47.91 (-/78)	-	-	-	-
	Liu SM 2012	Tibet	2003	1756	-	-	18.79/19.36 (33/34)	-	-	-	-
	Li KQ 2007	Hebei	2004	20716	10343/10373	2669/18047	43.30/- (897/-)	-	-		
	Li N 2008	Liaoning	2004	13358	6610/6748	5517/7841	22.23/72.09 (297/963)	-/44.63 (-/295)	-/98.99 (-/668)	-/62.53 (-/345)	-/78.82 (-/618)
	Ruan Y 2010	Yunnan	2005	5033	2416/2617	2431/2602	26.43/64.38 (133/324)	-/41.39 (-/100)	-/85.59 (-/224)	-/77.71 (-/189)	-/51.88 (-/135)
	Zhang XN 2010	Liaoning	2007	5059	2314/2745	3204/1855	-/13.44 (-/68)	-/11.24 (-/26)	-/15.30 (-/42)	-/12.17 (-/39)	-/15.63 (-/29)
	Wei B 2010	Guangxi	2007	18219	9196/9023	7028/11191	1.26/1.26 (23/23)	-/1.41 (-/13)	-/1.11 (-/10)	-/1.14 (-/8)	-/1.34 (-/15)
	Chen HM 2012	Hebei	2010	2360	1167/1193	565/1795	25.42/29.66 (60/70)	23.14/- (27/-)	27.66/- (33/-)	12.39/- (7/-)	29.53/- (53/-)
	Liu ZR 2013	Beijing	2010	2469	967/1502	-	31.59/59.54 (78/147)	24.82/45.50 (24/44)	35.95/68.58 (54/103)	-	-
	Ma X 2012	Beijing	2010	16032	-	-	31.87/43.41 (511/696)	-	-	-	-
	Wang WQ 2013	Fujian	2010	10764	4957/5807	8588/2176	5.57/7.90 (60/85)	-	-	-	-
	Liu J 2012	Henan	2011	29636	15042/14594	14731/14905	8.27/10.87 (245/322)	6.05/- (91/-)	10.55/- (154/-)	9.78/- (144/-)	6.78/- (101/-)
	Lu DY 2015	Shandong	2011	2373	1354/1019	-	5.48/5.90 (13/14)	-	-	-	-
	Li T 2013	Ningxia	2012	4156	1766/2390	0/41576	57.51/172.52 (239/717)	-	-	-	-

Subtypes	Study	Location	Year	Sample			Current/Life-time prevalence (‰)				
				overall	male/female	urban/rural	overall (n)	male (n)	female (n)	urban (n)	rural (n)
Generalized anxiety disorder	M R Phillips 2009	Zhejiang	2001	14639	7176/7463	4824/9815	3.35/- (49/-)	2.23/- (16/-)	4.42/- (33/-)	4.56/- (22/-)	2.75/- (27/-)
		Qinghai	2002	11178	5652/5526	2632/8546	14.49/- (162/-)	-	-	-	-
		Shandong	2004	22718	10457/12261	5660/17059	6.95/- (158/-)	-	-	-	-
		Gansu	2005	10249	5517/4730	1645/8604	6.44/- (66/-)	-	-	-	-
		Shandong	2005	4776	2211/2565	2380/2396	31.41/- (150/-)	-	-	-	-
	Lee S 2007	Beijing/Shanghai	2001	5201	-	-	-/8.08 (-/42)	-	-	-	-
	Ma X 2009	Beijing	2003	5926	2735/3191	3445/2481	-/11.98 (-/71)	-/6.58 (-/18)	-/16.60 (-/53)	-/10.45 (-/36)	-/14.11 (-/35)
	Liu SM 2012	Tibet	2003	1756	-	-	-/5.69 (-/10)	-	-	-	-
	Li KQ 2007	Hebei	2004	20716	10343/10373	2669/18047	6.13/6.13 (127/127)	3.77/- (39/-)	8.48/- (88/-)	4.87/- (13/-)	6.32/- (114/-)
	Li N 2008	Liaoning	2004	13358	6610/6748	5517/7841	3.07/6.96 (41/93)	-/3.93 (-/26)	-/9.93 (-/67)	-/7.61 (-/42)	-/6.38 (-/50)
	Ruan Y 2010	Yunnan	2005	5033	2416/2617	2432/2601	1.59/7.95 (8/40)	-/7.04 (-/17)	-/8.79 (-/23)	-/9.87 (-/24)	-/6.150 (-/16)
	Zhao ZH 2009	Guangdong	2006	7418	3226/4192	5544/1874	3.64/3.64 (27/27)	1.55/- (5/-)	5.25/- (22/-)	3.61/- (20/-)	3.74/- (7/-)
	Zhang XN 2010	Liaoning	2007	5059	2314/2745	3204/1855	-/4.15 (-/21)	-/3.46 (-/8)	-/4.74 (-/13)	-/4.37 (-/14)	-/3.77 (-/7)
	Wei B 2010	Guangxi	2007	18219	9196/9023	7028/11191	0.60/0.60 (11/11)	-/0.54 (-/5)	-/0.66 (-/6)	-/0.57 (-/4)	-/0.63 (-/7)
	Yu JC 2010	Guangdong	2009	2707	1300/1407	1902/805	0.74/1.11 (2/3)	-/0.00 (-/0)	-/2.13 (-/3)	-/1.05 (-/2)	-/1.24 (-/1)
	Fang X 2011	Fujian	2009	9986	5079/4907	3746/6240	8.31/- (83/-)	4.73/- (24/-)	12.02/- (59/-)	6.41/- (24/-)	9.46/- (59/-)
	Chen XL 2012	Shannxi	2010	2447	950/1497	1143/1308	0.41/0.82 (1/2)	-/0.00 (-/0)	-/1.34 (-/2)	-/0.87 (-/1)	-/0.77 (-/1)
	Liu ZR 2013	Beijing	2010	2469	967/1502	-	2.43/3.24 (6/8）	3.10/3.10 (3/3）	2.00/3.33 (3/5）	-	-
	Wang WQ 2013	Fujian	2010	10764	4957/5807	8588/2176	0.46/0.46 (5/5)	-	-	-	-
	Liu J 2012	Henan	2011	29636	15042/14594	14731/14905	5.1/6.88 (151/204)	-	-	-	-
	Lu DY 2015	Shandong	2011	2373	1354/1019	-	4.64/4.64 (11/11)	-	-	-	-

Subtypes	Study	Location	Year	Sample			Current/Life-time prevalence (‰)				
				overall	male/female	urban/rural	overall (n)	male (n)	female (n)	urban (n)	rural (n)
Non-specific anxiety disorder	M R Phillips 2009	Zhejiang	2001	14639	7176/7463	4824/9815	8.74/- (128/-)	5.30/- (38/-)	12.06/- (90/-)	14.30/- (69/-)	6.01/- (59/-)
		Qinghai	2002	11178	5652/5526	2632/8546	3.22/- (36/-)	-	-	-	-
		Shandong	2004	22718	10457/12261	5660/17059	21.70/- (493/-)	-	-	-	-
		Gansu	2005	10249	5517/4730	1645/8604	14.15/- (145/-)	-	-	-	-
		Shandong	2005	4776	2211/2565	2380/2396	3.98/- (19/-)	-	-	-	-
	Li KQ 2007	Hebei	2004	20716	10343/10373	2669/18047	21.09/21.24 (437/440)	-	-	-	-
	Fang X 2011	Fujian	2009	9986	5079/4907	3746/6240	5.91/- (59/-)	4.73/- (24/-)	7.13/- (35/-)	5.61/- (21/-)	6.09/- (38/-)
	Wang WQ 2013	Fujian	2010	10764	4957/5807	8588/2176	3.16/4.37 (34/47)	2.22/- (11/-)	3.96/- (23/-)	3.49/- (30/-)	1.84/- (4/-)
	Liu J 2012	Henan	2011	29636	15042/14594	14731/14905	0.78/0.91 (23/27)	-	-	-	-
	Lu DY 2015	Shandong	2011	2373	1354/1019	-	0.84/1.26 (2/3)	-	-	-	-
Panic disorder	M R Phillips 2009	Zhejiang	2001	14639	7176/7463	4824/9815	0.89/- (13/-)	0.56/- (4/-)	1.21/- (9/-)	1.04/- (5/-)	0.82/- (8/-)
		Qinghai	2002	11178	5652/5526	2632/8546	0.54/- (6/-)	-	-	-	-
		Shandong	2004	22718	10457/12261	5660/17059	0.48/- (11/-)	-	-	-	-
		Gansu	2005	10249	5517/4730	1645/8604	1.76/- (18/-)	-	-	-	-
		Shandong	2005	4776	2211/2565	2380/2396	1.88/- (9/-)	-	-	-	-
	Lee S 2007	Beijing/ Shanghai	2001	5201	-	-	-/4.04 (-/21)	-	-	-	-
	Liu SM 2012	Tibet	2003	1756	-	-	-/8.54 (-/15)	-	-	-	-
	Li KQ 2007	Hebei	2004	20716	10343/10373	2669/18047	2.65/3.57 (55/74)	1.55/- (16/-)	3.76/- (39/-)	0.75/- (2/-)	2.94/- (53/-)
	Li N 2008	Liaoning	2004	13358	6610/6748	5517/7841	1.72/3.44 (23/46)	-/1.51 (-/10)	-/5.33 (-/36)	-/3.08 (-/17)	-/3.70 (-/29)
	Ruan Y 2010	Yunnan	2005	5033	2416/2617	2432/2601	0.20/1.19 (1/6）	-/1.24 (-/3)	-/1.15 (-/3)	-/0.82 (-/2)	-/1.54 (-/4)
	Zhao ZH 2009	Guangdong	2006	7418	3226/4192	5544/1874	1.48/3.24 (11/24)	-	-	-	-
	Zhang XN 2010	Liaoning	2007	5059	2314/2745	3204/1855	-/9.09 (-/46)	-/6.91 (-/16)	-/10.93 (-/30)	-/8.11 (-/26)	-/10.78 (-/20)
	Yu JC 2010	Guangdong	2009	2707	1300/1407	1902/805	1.11/2.22 (3/6)	-/2.31 (-/3)	-/2.13 (-/3)	-/2.63 (-/5)	-/1.24 (-/1)

Subtypes	Study	Location	Year	Sample			Current/Life-time prevalence (‰)				
				overall	male/female	urban/rural	overall (n)	male (n)	female (n)	urban (n)	rural (n)
**Panic disorder**	Fang X 2011	Fujian	2009	9986	5079/4907	3746/6240	2.10/- (21/-)	1.77/- (9/-)	2.45/- (12/-)	1.87/- (7/-)	2.24/- (14/-)
	Chen XL 2012	Shannxi	2010	2447	950/1497	1143/1311	0.41/2.45 (1/6)	-/2.11 (-/2)	-/2.67 (-/4)	-/2.62 (-/3)	-/2.30 (-/3)
	Liu ZR 2013	Beijing	2010	2469	967/1502	-	0.81/5.67 (2/14)	1.03/3.10 (1/3)	0.67/7.32 (1/11)	-	-
	Wang WQ 2013	Fujian	2010	10764	4957/5807	8588/2176	0.19/0.19 (2/2)	-	-	-	-
	Liu J 2012	Henan	2011	29636	15042/14594	14731/14905	0.37/0.44 (11/13)	-	-	-	-
	Li T 2013	Ningxia	2012	4156	1766/2390	0/41576	3.85/10.11 (16/42)	-	-	-	-
**Social phobia**	M R Phillips 2009	Zhejiang	2001	14639	7176/7463	4824/9815	0.27/- (4/-)	-	-	-	-
		Qinghai	2002	11178	5652/5526	2632/8546	0.00/- (0/-)	-	-	-	-
		Shandong	2004	22718	10457/12261	5660/17059	0.18/- (4/-)	-	-	-	-
		Gansu	2005	10249	5517/4730	1645/8604	1.17/- (12/-)	-	-	-	-
		Shandong	2005	4776	2211/2565	2380/2396	0.21/- (1/-)	-	-	-	-
	Lee S 2007	Beijing/Shanghai	2001	5201	-	-	-/5.00 (-/26)	-	-	-	-
	Li KQ 2007	Hebei	2004	20716	10343/10373	2669/18047	1.54/1.64 (32/34)	1.06/- (11/-)	2.02/- (21/-)	1.50/- (4/-)	1.55/- (28/-)
	Li N 2008	Liaoning	2004	13358	6610/6748	5517/7841	4.79/13.48 (64/180)	-/10.74 (-/71)	-/16.15 (-/109)	-/9.61 (-/53)	-/16.20 (-/127)
	Ruan Y 2010	Yunnan	2005	5033	2416/2617	2432/2601	0.99/3.38 (5/17)	-/1.24 (-/3)	-/5.35 (-/14)	-/4.52 (-/11)	-/2.31 (-/6)
	Duan WD 2011	Guangdong	2005	7134	3615/3519	-	3.36/14.86 (24/106)	3.32/16.04 (12/58)	3.41/13.64 (12/48)	-	-
	Wei B 2010	Guangxi	2007	18219	9196/9023	7028/11191	0.05/0.05 (1/1)	-/0.11 (-/1)	-/0.00 (-/0)	-/0.14 (-/1)	-/0.00 (-/0)
	Yu JC 2010	Guangdong	2009	2707	1300/1407	1902/805	0.74/2.96 (2/8)	-/2.31 (-/3)	-/3.55 (-/5)	-/2.10 (-/4)	-/4.97 (-/4)
	Fang X 2011	Fujian	2009	9986	5079/4907	3746/6240	1.50/- (15/-)	1.77/- (9/-)	1.22/- (6/-)	1.60/- (6/-)	1.44/- (9/-)
	Chen XL 2012	Shannxi	2010	2447	950/1497	1143/1305	1.23/5.31 (3/13)	-/4.21 (-/4)	-/6.01 (-/9)	-/5.25 (-/6)	-/5.36 (-/7)
	Liu ZR 2013	Beijing	2010	2469	967/1502	-	4.46/8.51 (11/21)	4.14/7.24 (4/7)	4.66/9.32 (7/14)	-	-
	Wang WQ 2013	Fujian	2010	10764	4957/5807	8588/2176	0.19/0.19 (2/2)	-	-	-	-
	Liu J 2012	Henan	2011	29636	15042/14594	14731/14905	0.3/0.3 (9/9)	-	-	-	-
	Li T 2013	Ningxia	2012	4156	1766/2390	0/41576	1.92/10.11 (8/42)	-	-	-	-

Subtypes	Study	Location	Year	Sample			Current/Life-time prevalence (‰)				
				overall	male/female	urban/rural	overall (n)	male (n)	female (n)	urban (n)	rural (n)
Agoraphobia	M R Phillips 2009	Zhejiang	2001	14639	7176/7463	4824/9815	0.61/- (9/-)	0.00/- (0/-)	1.21/- (9/-)	0.41/- (2/-)	0.71/- (7/-)
		Qinghai	2002	11178	5652/5526	2632/8546	0.18/- (2/-)	-	-	-	-
		Shandong	2004	22718	10457/12261	5660/17059	0.04/- (1/-)	-	-	-	-
		Gansu	2005	10249	5517/4730	1645/8604	0.29/- (3/-)	-	-	-	-
		Shandong	2005	4776	2211/2565	2380/2396	0.00/- (0/-)	-	-	-	-
	Lee S 2007	Beijing/Shanghai	2001	5201	-	-	-/0.00 (-/0)	-	-	-	-
	Li KQ 2007	Hebei	2004	20716	10343/1033	2669/18047	0.39/0.43 (8/9)	-	-	-	-
	Li N 2008	Liaoning	2004	13358	6610/6748	5517/7841	8.46/30.39 (113/406)	-/16.04 (-/106)	-/44.46 (-/300)	-/9.97 (-/55)	-/29.97 (-/235)
	Ruan Y 2010	Yunnan	2005	5033	2416/2617	2432/2601	2.78/9.93 (14/50)	-/4.14 (-/10)	-/15.28 (-/40)	-/11.51 (-/28)	-/8.46 (-/22)
	Duan WD 2011	Guangdong	2005	7134	3615/3519	-	0.28/2.52 (2/18)	0.28/1.66 (1/6)	0.28/3.41 (1/12)	-	-
	Yu JC 2010	Guangdong	2009	2707	1300/1407	1902/805	0.00/0.37 (0/1)	-/0.00 (-/0)	-/0.71 (-/1)	-/0.00 (-/0)	-/1.24 (-/1)
	Fang X 2011	Fujian	2009	9986	5079/4907	3746/6240	0.70/- (7/-)	-	-	-	-
	Chen XL 2012	Shannxi	2010	2447	950/1497	1143/1307	0.41/1.63 (1/4)	-/0.00 (-/0)	-/2.67 (-/4)	-/0.87 (-/1)	-/2.30 (-/3)
	Liu ZR 2013	Beijing	2010	2469	967/1502	-	0.81/1.22 (2/3)	0.00/0.00 (0/0)	1.33/2.00 (2/3)	-	-
	Wang WQ 2013	Fujian	2010	10764	4957/5807	8588/2176	0.19/0.19 (2/2)	-	-	-	-
	Liu J 2012	Henan	2011	29636	15042/14594	14731/14905	0.07/0.10 (2/3)	-	-	-	-
	Li T 2013	Ningxia	2012	4156	1766/2390	0/41576	1.68/7.70 (7/32)	-	-	-	-
Specific phobia	M R Phillips 2009	Zhejiang	2001	14639	7176/7463	4824/9815	4.03/- (59/-)	2.09/- (15/-)	5.90/- (44/-)	2.69/- (13/-)	4.69/- (46/-)
		Qinghai	2002	11178	5652/5526	2632/8546	0.00/- (0/-)	-	-	-	-
		Shandong	2004	22718	10457/12261	5660/17059	0.53/- (12/-)	-	-	-	-
		Gansu	2005	10249	5517/4730	1645/8604	2.93/- (30/-)	-	-	-	-
		Shandong	2005	4776	2211/2565	2380/2396	0.00/- (0/-)	-	-	-	-
	Lee S 2007	Beijing/Shanghai	2001	5201	-	-	-/25.96 (-/135)	-	-	-	-
	Liu SM 2012	Tibet	2003	1756	-	-	-/5.13 (-/9)	-	-	-	-
	Li KQ 2007	Hebei	2004	20716	10343/10373	2669/18047	6.71/7.43 (139/154)	4.45/- (46/-)	8.94/- (93/-)	1.50/- (4/-)	7.48/- (135/-)

Subtypes	Study	Location	Year	Sample			Current/Life-time prevalence (‰)				
				overall	male/female	urban/rural	overall (n)	male (n)	female (n)	urban (n)	rural (n)
Specific phobia	Ruan Y 2010	Yunnan	2005	5033	2416/2617	2432/2601	20.27/46.89 (102/236)	-/28.15 (-/68)	-/64.20 (-/168)	-/57.57 (-/140)	-/36.91 (-/96)
	Duan WD 2011	Guangdong	2005	7134	3615/3519	-	10.65/36.02 (76/257)	5.26/24.90 (19/90)	16.20/47.46 (57/167)	-	-
	Yu JC 2010	Guangdong	2009	2707	1300/1407	1902/805	5.17/9.60 (14/26)	-/6.15 (-/8)	-/12.79 (-/18)	-/10.52 (-/20)	-/7.45 (-/6)
	Fang X 2011	Fujian	2009	9986	5079/4907	3746/6240	4.41/- (44/-)	2.76/- (14/-)	6.11/- (30/-)	9.61/- (36/-)	1.28/- (8/-)
	Chen XL 2012	Shannxi	2010	2447	950/1497	1143/1306	10.63/19.21 (26/47)	-/9.47 (-/9)	-/25.38 (-/38)	-/10.50 (-/12)	-/26.78 (-/35)
	Liu ZR 2013	Beijing	2010	2469	967/1502	-	19.85/30.78 (49/76)	12.41/21.72 (12/21)	24.63/36.62 (37/55)	-	-
	Wang WQ 2013	Fujian	2010	10764	4957/5807	8588/2176	1.21/1.39 (13/15)	1.01/- (5/-)	1.38/- (8/-)	1.28/- (11/-)	0.92/- (2/-)
	Liu J 2012	Henan	2011	29636	15042/14594	14731/14905	0.74/0.91 (22/27)	-	-	-	-
	Li T 2013	Ningxia	2012	4156	1766/2390	0/41576	24.06/42.59 (100/177)	-	-	-	-
Post-traumatic stress disorder	M R Phillips 2009	Zhejiang	2001	14639	7176/7463	4824/9815	3.62/- (53/-)	2.09/- (15/-)	5.09/- (38/-)	4.56/- (22/-)	3.16/- (31/-)
		Qinghai	2002	11178	5652/5526	2632/8546	0.98/- (11/-)	-	-	-	-
		Shandong	2004	22718	10457/12261	5660/17059	0.40/- (9/-)	-	-	-	-
		Gansu	2005	10249	5517/4730	1645/8604	1.85/- (19/-)	-	-	-	-
		Shandong	2005	4776	2211/2565	2380/2396	1.05/- (5/-)	-	-	-	-
	Lee S 2007	Beijing/ Shanghai	2001	1628	-		-/3.07 (-/5)	-	-	-	-
	Liu SM 2012	Tibet	2003	1756	-	-	-/2.85 (-/5)	-	-	-	-
	Li KQ 2007	Hebei	2004	20716	10343/10373	2669/18047	3.19/5.02 (66/104)	2.71/- (28/-)	3.66/- (38/-)	1.50/- (4/-)	3.44/- (62/)-
	Ruan Y 2010	Yunnan	2005	5033	2416/2617	2432/2601	2.58/6.56 (13/33)	-/6.62 (-/16)	-/6.50 (-/17)	-/3.29 (-/8)	-/9.61 (-/25)
	Zhao ZH 2009	Guangdong	2006	7418	3226/4192	5544/1874	0.94/3.1 (7/23)	-	-	-	-
	Wei B 2010	Guangxi	2007	18219	9196/9023	7028/11191	0.22/0.22 (4/4)	-/0.43 (-/4)	-/0.00 (-/0)	-/0.00 (-/0)	-/0.36 (-/4)
	Yu JC 2010	Guangdong	2009	2707	1300/1407	1902/805	0/2.59 (0/7)	-/0.00 (-/0)	-/4.98 (-/7)	-/2.63 (-/5)	-/2.48 (-/2)
	Fang X 2011	Fujian	2009	9986	5079/4907	3746/6204	1.20/- (12/-)	-	-	-	-
	Chen XL 2012	Shannxi	2010	2447	950/1497	1143/1312	0.00/0.00 (0/0)	-/0.00 (-/0)	-/0.00 (-/0)	-/0.00 (-/0)	-/0.00 (-/0)
	Wang WQ 2013	Fujian	2010	10764	4957/5807	8588/2176	0.37/1.11 (4/12)	-	-	-	-
	Liu J 2012	Henan	2011	29636	15042/14594	14731/14905	0.27/0.44 (8/13)	-	-	-	-

Subtypes	Study	Location	Year	Sample			Current/Life-time prevalence (‰)				
				overall	male/female	urban/rural	overall (n)	male (n)	female (n)	urban (n)	rural (n)
Obsessive compulsive disorder	M R Phillips 2009	Zhejiang	2001	14639	7176/7463	4824/9815	0.34/- (5/-)	0.56/- (4/-)	0.13/- (1/-)	0.62/- (3/-)	0.20/- (2/-)
		Qinghai	2002	11178	5652/5526	2632/8546	0.18/- (2/-)	-	-	-	-
		Shandong	2004	22718	10457/12261	5660/17059	0.48/- (11/-)	-	-	-	-
		Gansu	2005	10249	5517/4730	1645/8604	1.37/- (14/-)	-	-	-	-
		Shandong	2005	4776	2211/2565	2380/2396	0.63/- (3/-)	-	-	-	-
	Li KQ 2007	Hebei	2004	20716	10343/10373	2669/18047	1.40/1.54 (29/32)	1.26/- (13/-)	1.54/- (16/-)	2.62/- (7/-)	1.22/- (22/)-
	Ruan Y 2010	Yunnan	2005	5033	2416/2617	2432/2601	0.99/1.99 (5/10)	-/1.24 (-/3)	-/2.67 (-/7)	-/1.64 (-/4)	-/2.31 (-/6)
	Zhang XN 2010	Liaoning	2007	5059	2314/2745	3204/1855	-/0.79 (-/4)	-/0.86 (-/2)	-/0.73 (-/2)	-/0.31 (-/1)	-/1.62 (-/3)
	Yu JC 2010	Guangdong	2009	2707	1300/1407	1902/805	1.11/5.17 (3/14)	-/4.62 (-/6)	-/5.69 (-/8)	-/4.73 (-/9)	-/6.21 (-/5)
	Fang X 2011	Fujian	2009	9986	5079/4907	3746/6240	1.40/- (14/-)	-	-	-	-
	Chen XL 2012	Shannxi	2010	2447	950/1497	1143/1309	3.27/10.22 (8/25)	-/5.26 (-/5)	-/13.36 (-/20)	-/8.75 (-/10)	-/11.48 (-/15)
	Liu ZR 2013	Beijing	2010	2469	967/1502	-	7.70/25.11 (19/62)	6.20/16.55 (6/16)	9.32/30.63 (14/46)	-	-
	Wang WQ 2013	Fujian	2010	10764	4957/5807	8588/2176	0.28/0.56 (3/6)	-	-	-	-
	Liu J 2012	Henan	2011	29636	15042/14594	14731/14905	0.64/0.88 (19/26)	-	-	-	-
	Li T 2013	Ningxia	2012	4156	1766/2390	0/41576	3.61/7.46 (15/31)	-	-	-	-

Table S2. Literature quality score scale for accessing risk of bias

References	Year	Location	response rate (%)	items					Total scores
				S1*	S2*	S3*	P1*	P2*	
M R Phillips 2009	2001	Zhejiang	97.50	2	2	2	2	2	10
	2002	Qinghai	93.14	2	2	2	2	2	10
	2004	Shandong	94.62	2	2	2	2	2	10
	2005	Gansu	90.23	2	2	2	2	2	10
	2005	Shandong	99.50	2	2	2	2	2	10
Lee S 2007	2001	Beijing/shanghai	74.8/74.6	2	2	2	2	2	10
Ma X 2009	2003	Beijing	94.56	2	2	2	2	2	10
Liu SM 2012	2003	Tibet	above 90	2	2	2	2	1	9
Su KQ 2007	2004	Hebei	86.32	2	1	2	2	2	9
Li N 2008	2004	Liaoning	86.09	2	2	2	2	2	10
Lu J 2009	2005	Yunnan	82.60	2	1	1	2	2	8
Duan WD 2011	2005	Guangdong	79.98	2	1	1	2	2	8
Zhao ZH 2009	2006	Guangdong	-	2	2	0	2	2	8
Zhang XN 2010	2007	Liaoning	95.81	2	2	2	2	2	10
Wei B 2010	2007	Guangxi	85.58	2	2	2	2	2	10
Yu JC 2010	2009	Guangdong	82.21	2	2	1	2	2	9
Fang X 2011	2009	Fujian	86.58	2	2	2	2	2	10
Chen HM 2012	2010	Hebei	85.82	2	2	2	2	2	10
Chen XL 2012	2010	Shannxi	69.87	2	2	0	2	2	8
Liu ZR 2013	2010	Beijing	72.90	2	2	1	2	2	9
Ma X 2012	2010	Beijing	80.70	2	1	1	2	1	7
Wang WQ 2013	2010	Fujian	89.17	2	1	2	2	2	9
Liu J 2012	2011	Henan	98.79	2	2	2	2	2	10
Lu DY 2015	2011	Guangdong	-	2	2	0	2	1	7
Li T 2013	2012	Ningxia	88.80	2	2	2	2	2	10

S1*: Selection (sample population)

S2*: Selection (sample size)

S3*: Selection (participation)

P1*: Performance bias (outcome assessment)

P2*: Performance bias(analytical method to control for bias)

Table S3. Meta-regression for prevalence of ADs

Variables	current prevalence					lifetime prevalence				
	Covariates	No. study	P*	Coef. (95%CI)	P-value	Covariates	No. study	P*	Coef. (95%CI)	P-value
year	-	17	24.47	-0.13 (-2.39,2.12)	0.90	-	13	41.12	1.41 (-7.14,9.97)	0.72
sample size	-	17	24.47	-0.00 (-0.00,0.00)	0.47	-	13	41.12	-0.00 (-0.00,0.00)	0.39
province	Zhejiang(Ref.)	1	21.86			Yunnan(Ref.)	1	64.38		
	Beijing	2	31.83	9.88 (-20.89,40.65)	0.59	Beijing	2	50.85	-12.99 (-174.25,148.27)	0.76
	Fujian	1	5.57	-16.29 (-51.18, 18.60)	0.45	Beijing/ Shanghai	1	47.91	-16.47 (-203.46,170.52)	0.74
	Gansu	1	29.95	8.09 (-27.13,43.31)	0.70	Fujian	1	7.90	-56.48 (-242.11,129.15)	0.32
	Guangdong	1	5.48	-16.38 (-51.53,18.77)		Guangdong	1	5.90	108.14 (-79.16,295.44)	0.13
	Guangxi	1	1.26	-20.60 (-55.42,14.22)	0.35	Guangxi	1	1.26	-63.12 (-248.72,122.48)	0.28
	Hebei	2	34.60	13.08 (-17.63,43.79)	0.50	Hebei	1	29.66	-34.72 (-220.92,151.48)	0.51
	Henan	1	8.27	-13.59 (-53.96,54.70)	0.99	Henan	1	10.87	-53.51 (-239.12,132.10)	0.34
	Liaoning	1	22.23	0.37 (-34.68,35.42)	0.15	Liaoning	2	42.75	-21.65 (-182.66,139.36)	0.62
	Ningxia	1	57.51	35.65 (-1.01,72.31)	0.91	Ningxia	1	172.52	108.14 (-79.16,295.44)	0.13
	Qinghai	1	19.59	-2.27 (-37.33,32.79)	0.85	Tibet	1	19.36	-45.02 (-231.15,141.11)	0.41
	Shandong	2	34.57	12.88 (-17.69,43.46)	0.88					
	Tibet	1	18.79	4.57 (-30.98,40.12)	0.83					
	Yunnan	1	26.43	21.86 (-2.91,46.63)	0.19					
territorial level	provincial(Ref.)	9	27.37			provincial(Ref.)	6	59.13		
	city	8	21.10	-6.00 (-21.27,9.27)	0.42	city	7	26.21	-31.85 (-86.51,22.79)	0.23
Risk of bias assessment	7(Ref.)	2	18.68			9(Ref.)	2	39.30		
	8	1	26.43	7.75 (-34.60,50.10)	0.70	7	2	24.65	-14.75 (-131.73,102.22)	0.78
	9	4	24.79	6.08 (-34.60,50.11)	0.67	8	1	64.38	24.97 (-118.40,168.35)	0.70
	10	10	25.31	6.72 (-34.60,50.12)	0.60	10	8	42.62	4.85 (-87.73,97.43)	0.91
diagnostic tool	SCID(Ref.)	10	24.20			SCID(Ref.)	6	19.36		
	CIDI	7	24.98	0.68 (-15.16,16.53)	0.93	CIDI	7	61.30	41.81 (-9.74,93.38)	0.10
identity of investigators	psychiatrist(Ref.)	13	23.11			psychiatrist(Ref.)	9	26.84		
	others	4	28.96	5.80 (-12.31,23.91)	0.51	others	4	73.04	44.88 (-10.94,100.70)	0.10

ADs: anxiety disorders,

No. study =number of studies, P* = Estimated prevalence was calculated separately, Coef. = Regression coefficient,

Ref. = Reference category

Table S4a. Results of sensitivity analysis: the results of sensitivity analysis in ADs and its subtypes.

Subtypes	Current prevalence			Lifetime prevalence		
		Estimate	95% CI		Estimate	95% CI
ADs	Combined	7.75	(7.36, 8.14)	Combined	5.73	(5.30,6.17)
	Wei B 2010*	16.54	(15.94, 17.14)	Wei B 2010*	16.77	(15.97,17.58)
GAD	Combined	1.99	(1.79, 2.19)	Combined	1.77	(1.55, 2.00)
	Wei B 2010*	2.65	(2.40, 2.89)	Wei B 2010*	2.56	(2.27, 2.85)
	Wang WQ 2013*	2.49	(2.26, 2.72)	Wang WQ 2013*	2.36	(2.09, 2.63)
	Liu J 2012*	1.79	(1.58, 2.00)	Liu J 2012*	1.46	(1.23,1.69)
NSAD	Combined	2.51	(2.25, 2.77)	-	-	-
	Liu J 2012*	6.35	(5.88, 6.82)			
PD	Combined	0.59	(0.47, 0.70)	Combined	0.76	(0.60, 0.93)
	None			Wang WQ 2013*	1.04	(0.82, 0.93)
				Liu J 2012 *	0.74	(0.57, 0.90)
SP	Combined	0.00	(0.00, 0.01)	Combined	0.23	(0.14, 0.31)
	M R Phillips 2009*	0.23	(0.16, 0.30)	Wei B 2010*	0.60	(0.45, 0.75)
AP	Combined	0.01	(-0.01, 0.02)	Combined	0.00	(-0.01, 0.02)
	M R Phillips 2009*	0.02	(0.00, 0.04	Lee S 2007*	0.24	(0.14, 0.33)
SPP	Combined	0.00	(0.00, 0.01)	Combined	2.09	(1.80, 2.38）
	None			Liu J 2012*	5.28	（4.72, 5.85)
PTSD	Combined	0.01	(0.00, 0.03)	Combined	0.02	(-0.01, 0.04)
	None			Liu J 2012*	0.58	(0.43, 0.73)
OCD	Combined	0.53	(0.42, 0.65)	-	-	-
	None					

Table S4b. Results of sensitivity analysis: the characteristics of the studies those influenced the pooled results.

References*	quality score	subtypes	$OR_{(M:F)}$; OR 95%CI	$OR_{(U:R)}$;OR 95%CI
Wei B 2010*	10	ADs-current	-	-
		GAD-current	-	-
		ADs-lifetime	1.28; (0.56, 2.91)	0.85; (0.36, 2.00)
		GAD-lifetime	0.82; (0.25, 2.68)	0.91; (0.27, 3.11)
		SP-lifetime	2.94; (0.12, 72.27)	4.78; (0.19, 117.29)
Liu J 2012*	10	GAD-current	-	-
		NSAD-current	-	-
		GAD-lifetime	-	-
		PD-lifetime	-	-
		SPP-lifetime	-	-
		PTSD-lifetime	-	-
Wang WQ 2013*	9	GAD-current	-	-
		GAD-lifetime	-	-
		PD-lifetime	-	-
M R Phillips 2009*	10	SP-current	-	-
		AP-current	-	-
Lee S 2007*	10	AP-lifetime	-	-

*result was significantly changed when the study being omitted.

ADs: anxiety disorders; GAD: generalized anxiety disorder; NSAD: non-specific anxiety disorder;

PD: panic disorder; SP: social phobia; AP: agoraphobia; PTSD: post-traumatic stress disorder;

-current: current prevalence

-lifetime: lifetime prevalence

OR: Odds Ratio

M:F: Males versus Females

U:R : Urban versus Rural

Table S5. Heterogeneity and publication bias of anxiety disorders

items	statistic methods	subtypes	current prevalence			lifetime prevalence		
			p value	95%CI	I^2(%)	p value	95%CI	I^2(%)
Heterogeneity	Stata	ADs	<0.001	(17.97,30.98)	99.50	<0.001	(31.09,51.15)	99.60
		GAD	<0.001	(3.72,6.63)	97.70	<0.001	(3.17,6.14)	96.80
		NSAD	<0.001	(4.49,12.10)	99.10	<0.001	(0.43,13.35)	99.30
		PD	<0.001	(0.74,1.43)	85.20	<0.001	(2.46,4.41)	94.80
		SP	<0.001	(0.48,0.92)	91.90	<0.001	(3.24,4.99)	97.50
		AP	<0.001	(0.10,0.28)	91.30	<0.001	(1.56,2.74)	98.20
		SPP	<0.001	(0.49,0.77)	98.00	<0.001	(15.18,24.04)	99.00
		PTSD	<0.001	(0.35,0.63)	93.80	<0.001	(1.23,2.43)	95.60
		OBD	<0.001	(0.58,1.22)	81.30	<0.001	(2.04,4.31)	93.40
Publication bias	Funnel plots	ADs	0.00	(1087.05,2479.52)	-	0.00	(612.26,2126.49)	-
		GAD	0.00	(1721.66, 4951.85)	-	0.00	(1457.11, 4039.40)	-
		NSAD	0.00	(2084.80, 4279.57)	-	-	-	-
		PD	0.00	(1804.64, 6400.28)	-	0.00	(1489.73, 4961.80)	-
		SP	0.00	(21844.59, 56808.9)	-	0.01	(1565.00, 9706.32)	-
		AP	0.00	(22122.10, 67184.13)	-	0.02	(4684.15, 53335.10)	-
		SPP	0.02	(4955.80, 40300.19)	-	0.00	(842.66, 2269.31)	-
		PTSD	0.00	(19737.93, 44537.53)	-	0.02	(5159.28, 39643.12)	-
		OBD	0.01	(944.89, 6078.47)	-	-	-	-
	Egger's tests	ADs	0.00	(10.15,21.78)	-	0.00	(8.87,24.39)	-
		GAD	0.00	(5.19, 13.02)	-	0.00	(2.90, 9.76)	-
		NSAD	0.01	(4.81, 20.86)	-	-	-	-
		PD	0.00	(1.89, 5.34)	-	0.00	(3.32, 6.85)	-
		SP	0.00	(2.03, 4.07)	-	0.00	(3.91, 9.03)	-
		AP	0.00	(1.33, 4.18)	-	0.03	(0.78, 9.29)	-
		SPP	0.00	(5.07, 8.41)	-	0.00	(6.79, 14.81)	-
		PTSD	0.00	(2.75, 5.23)	-	0.00	(2.22, 6.38)	-
		OBD	0.00	(2.12, 4.78)	-	-	-	-

ADs: anxiety disorders; GAD: generalized anxiety disorder; NSAD：non-specific anxiety disorder;
PD: panic disorder; SP: social phobia; AP: agoraphobia; SPP: specific phobia;
PTSD: post-traumatic stress disorder; OCD: obsessive compulsive disorder

Data S1: The excluded studies in this meta-analysis

Excluded studies

We excluded 281 studies for different reasons, and details were described as below:

- ✧ Studies based on specific populations, regions or situations: 198
  - a. Seniors: 9 [1-9]
  - b. Children/ adolescents: 8 [10-17]
  - c. Graduate students/Undergraduate students/ Primary, middle or high school students: 26 [18-43]
  - d. The troops: 14 [44-57]
  - e. Populations suffered from earthquake: 45 [58-102]
  - f. Patients in hospital: 48 [103-150]
  - g. Criminals: 7 [151-157]
  - h. Population related with mining / steel/ railway industry: 9 [158-166]
  - i. Other kinds of special populations: 24 [167-190]
  - j. Review on special populations: 8 [191-198]
- ✧ Review: 14 [199-212]
- ✧ investigation time before 2000: 10 [213-222]
- ✧ No available data for the prevalence calculation: 2 [223,224]
- ✧ Not random sampling: 1 [225]
- ✧ county level: 4 [226-229]
- ✧ village level: 1 [230]
- ✧ Studies did not use structure diagnostic interviews with international diagnostic criteria as diagnostic tools: 2 [231,232]
- ✧ Data duplicated with included articles: 49

including the following types：

1) The study [233] we included was 4-provincal investigation, which contained in the five studies [234-238] we excluded in each four provinces. 6 city-level studies [239-244] were included in the province-wide study [238]conducted in Shandong.

2) Two studies [245,246] in Beijing and Shanghai were excluded for because they were repeated with one study we included [247].

3) One study [248] in Beijing duplicated with one paper included [249] were excluded

4) In Hebei province, the provincial study [250] we included contained 7 studies [251-257] of Baoding city, and repeat with another 7 studies [258-264].

5) In Liaoning province, 7 studies [265-271] were contained in and the province-wide study [272] included in the meta-analysis. Additionally, in Dalian city, there were 2 studies [273,274] were repetitions of the study included [275].

6) In Guangxi, there were 6 studies [276-281] we excluded for that they were the same studies with a included study [282].

7) In Yunnan, the study by Lu et al. [283],was repeated with the study we included in [284].

8) In Fujian, Xiamen city, a study [285] was repeated with a included study [286].

9) In Guangdong, a study in Yunfu city [287] was contained by the included study [288]; in Shenzhen city, a study [289] was excluded for that it was the same study with an included study [290].

10) In Ningxia, a study [291] was contained by a included study [292].

11) In Tibet, we excluded a study [293] for that it was the same studies with an included study [294].

References

1. Chou, K. L. Panic disorder in older adults: evidence from the national epidemiologic survey on alcohol and related conditions. *Int J Geriatr Psychiatry* **25**, 822-832; doi:10.1002/gps.2424 (2010).
2. Chou, K. L., Mackenzie, C. S., Liang, K. & Sareen, J. Three-year incidence and predictors of first-onset of DSM-IV mood, anxiety, and substance use disorders in older adults: results from Wave 2 of the National Epidemiologic Survey on Alcohol and Related Conditions. *J Clin Psychiatry* **72**, 144-155; doi:10.4088/JCP.09m05618gry (2011).
3. Li, Z. *et al.* Epidemiological survey on mental diseases in the elderly in Zhongshan City. *J Clin Psychiatry* **12**, 351 (2002).
4. Liu, Z. M. *A population-based research on the prevalence, influencing factors of mental disorders and relevant mental health sevice among elderly in rural community of Anhui Province* master thesis, Anhui Medici University (2010).
5. Lu, X. Y. *et al.* Epidemiological investigation on senile mental disorders in JiangXi Province. *Chin J Guide* **3**, 8-9 (2005).
6. Qu, W. Z. *et al.* Epidemiological characteristics of anxiety disorders in elderly in Pudong New District，Shanghai: A community-based study. *Chin J Clinicians (Electrinic Edition)* **5**, 1346-1349 (2011).
7. Yang, G. F. *et al.* Epidemic survey on depression and anxiety condition in in elderly in Qinhuangdao City of Hebei Province. *Geriatr Health Care* **14**, 181-192 (2008).
8. Zhang, X. *et al.* Generalized anxiety in community-dwelling elderly: Prevalence and clinical characteristics. *J Affect Disord* **172C**, 24-29; doi:10.1016/j.jad.2014.09.036 (2014).
9. Zhou, T. X., Zhang, S. P., Jiang, Y. Q. & Wang, J. M. An epidemiological survey on elderly neurouses. *ShanghaiArchives of Psychiatry* **12**, 125-131 (2000).
10. Su, L. Y. Investigation and the factors of the prevalence of mental disorders in adolescents aging from 13 to 16 in Hunan Province *Chinese Mental Health Journal* **7**, 221-224 (1993).
11. Sun, J., Boschen, M. J., Farrell, L. J., Buys, N. & Li, Z. J. Obsessive-compulsive symptoms in a normative Chinese sample of youth: prevalence, symptom dimensions, and factor structure of the Leyton Obsessional Inventory--Child Version. *J Affect Disord* **164**, 19-27; doi:10.1016/j.jad.2014.04.004 (2014).
12. Sun, J., Li, Z., Buys, N. & Storch, E. A. Correlates of comorbid depression, anxiety and helplessness with obsessive-compulsive disorder in Chinese adolescents. *J Affect Disord* **174**, 31-37; doi:10.1016/j.jad.2014.11.004 (2015).
13. Wang, F. S. *Study on social anxiety status and its influential factors of left-behind children in one rural area of Anhui Province* master thesis, Anhui medical university, (2008).
14. Wang, K. *A two year follow-up study of anxiety disorders in Children aged 7-10* master thesis, Zhongnan University, (2004).
15. Wei, B. G. Biologic factors for anxiety disorders in children and adolescents. *J Bengbu Med Coll* **26**, 471-472 (2001).
16. Xu, W. M. *Study on the prevalence of psychiatric disorders among children and adolescents in Liaoning Province*, China medical university, (2008).
17. Zhang, W. F. *Prevalence of psychiatric school children disorders among 6-17 year old in Liaoning Province* master thesis, Dalian medicial university, (2010).
18. Du, Q. F. *et al.* The analysis of social anxiety disorder prevalence rates and influencing factors of Chengdu primary school students *Chin J Behav Med & Brain Sci* **19**, 964-966 (2010).
19. Gao, Z. Y., Chen, X. J. & Lei, P. C. Epidemic survey on neuroses in 1542 college students. *Xianning medical college academic journal* **9**, 27-30 (1995).

20. Guan, B. Q. *et al.* Prevalence of psychiatric disorders in primary and middle school students in Hunan Province. *Chin J Contemp Pediatr* **12**, 123-127 (2010).
21. Huang, X. Y., Chen, J. X., Wang, H. W. & Huang, H. J. Prevalence of neurosis and its influencing factors in poor undergraduates. *Chin J Sch Health* **29**, 226-227 (2008).
22. Huang, Z. Z., Chen, X. W., Wu, X. N. & Fan, X. Y. The study on neurosis in students of university in Fuzhou City. *Sichuan Mental Health* **230**, 230-232 (1998).
23. Huang, Z. Z., Chen, X. W., Wu, X. N., Fan, X. Y. & Wang, J. L. Investigation on the prevalence of neurasthenia in medical college students. *Chinese Journal of School Doctor* **12**, 340-341 (1998).
24. Kou, C. G. *et al.* Prevalence survey on obsessive compulsive neurosis in college students. *Journal of Jilin University (Medicine Edition)* **35**, 384-388 (2009).
25. Mao, F. Q., Li, Z. T. & Wang, J. H. Description of mental diseases caught by graduate student. *Heath Psychology Journal* **4**, 63-64 (2003).
26. Nie, Y. S., Cao, J. Q., Zhou, Y. Q., Yang, J. & Yang, J. W. The prevalence rate and growth environmental factors of social anxiety disorder among Daqing college students. *Chin J Behav Med & Brain Sci* **23**, 793-796 (2014).
27. Su, C. *et al.* Epidemiological survey of social anxiety disorder in primary school pupils of Zhanjiang urban districts. *Chinese Mental Health Journal* **20**, 736-738 (2006).
28. Su, L. Y., Luo, X. R., yang, Z. W., Wan, G. B. & Li, X. R. Anxiety disorders in Chinese elementary school pupils. *Chin J Nerv Ment Dis* **29**, 330-333 (2003).
29. Sun, Y. J., Xu, P. & Zhang, L. H. Analysis and related factors of obsessive compulsive disorder in college Students. *Health Psychology Journal* **8**, 318-320 (2000).
30. Wang, W. Stress and Post-traumatic disorder in College Students. *Soft Science of Health* **25**, 52-54 (2011).
31. Wang, Z. M. *et al.* Epidemiologic situation of neurosis and its causes in some medical college in Henan. *Journal of Henan Medical University* **35**, 438-441 (2000).
32. Wu, C. D. *et al.* Epidemiological survey of middle school students aging from 14 to 18. *Hainan Med J* **2**, 60-63 (1991).
33. Xiao, R. *The prevalence and risk factors of social anxiety disorder in high schools and universities in Chengdu* Maste thesis, Sichuan University, (2004).
34. Xiao, R. *et al.* Co-morbidity of sicial anxiety disorder and social function in high school and university in Chengdu. *Chinese Jounal of Clinical Rehabilitation* **10**, 38-40 (2006).
35. Xiao, R. *et al.* Prevalence and risk factors of social anxiety disorder in high schools and universities in Chengdu. *Sichuan University Journal* **37**, 636-640 (2006).
36. Xie, B. *The epidemiology of obsessive compulsive disorder in undergraduates and association with SAPAP3 gene single nucleotide polymorphism* master thesis, Jilin University, (2007).
37. Xu, Y. H., Li, C. Y. & Mao, Y. T. Study on the state of neurosis of university students. *China Journal of Health Psychology* **19**, 152-154 (2011).
38. Ye, H. S. *The epidemiological investigation of anxiety disorders in urban and rural primary and middle school in Changsha* master thesis, Zhongnan University, (2007).
39. Ye, H. S., Luo, X. R., Zhou, X., Shen, X. Y. & Wang, X. H. The epidemiological investigation of anxiety disorders in urban-rural primary and middle school students in Huaihua. *J Clin Psychosom Dis* **14**, 501-504 (2008).
40. Zhang, K. *et al.* Prevalence of neurosis in a college. *Chinese Mental Health Journal* **17**, 339-341 (2003).
41. Zhang, Q. M. & Liu, C. Q. Epidemiological survey of social anxiety disorder in college and middle school students in Qingzhou City. *Journal of China Traditional Chinese Medicine Information* **3**, 43 (2011).
42. Zhang, X. Q. Prevalence of axiety disorders among students in Hefei. *Chinese Mental Health Journal* **18**, 726-734 (2004).

43. Zhang, Y. C. *A study of neurosis epidemiology characteristic analysis and preventing measures in college students* master thesis, Jilin University, (2005).
44. Chen, J. J., Wu, X. B. & Liu, Q. B. The prevalence of neuroses in a military force. *Chinese Mental Health Journal* **14**, 335-336 (2000).
45. Chen, J. J., Wu, X. B. & Liu, Q. B. The result Analysis on neurosis in a military force by SCL-9Q EPQ. *Health Psychology Journal* **18**, 112-114 (2000).
46. Gan, J. L. *et al.* An analysis on prevalence of mental illness in new soldiers from 1986 to 2001. *J Chin Psychol Med* **12**, 1-2 (2002).
47. Gao, P. Q. *et al.* Epidemiological Investigation on Neurosis in People's Armed Police. *Medical Journal of the Chinese People's Armed Police Forces* **10**, 100-101 (1999).
48. Li, W. Z. Epidemiological study on necrosis in People's Armed Police Forces. *Medical Journal of the Chinese People's Armed Police Forces* **10**, 100-101 (2001).
49. Li, Z. *et al.* Epidemiological investigation on neurosis of police in Zhongshan City. *Medical Journal of Chinese People's Health* **18**, 527-530 (2006).
50. Liu, Q. B. *et al.* Epidemiological survey and analysis on mental diseases in Shenyang millitary region. *Journal of Clinical Psychiatry* **4**, 94-95 (1994).
51. Wang, H. *et al.* Social anxiety disorder in the Chinese military: prevalence, comorbidities, impairment, and treatment-seeking. *Psychiatry Res* **220**, 903-908; doi:10.1016/j.psychres.2014.07.063 (2014).
52. Wang, H. L. *et al.* Epidemiological investigation on post-traumatic stress disorder in the Chinese People's Police Forces. *Hebei Mental Health* **9**, 1-4 (1996).
53. Xie, Y. Q. *Psychological health survey of inpatients in basic troop* Master thesis, Southern Medical University, (2013).
54. Yu, J. T. *et al.* Epidemiological investigation of post-traumatic stress disorder in army aviation regiment. *J Southeast Univ (Med Sci Edi)* **34**, 397-398 (2015).
55. Zhou, H. Y. *et al.* Sampling survey of anxiety and depression of grass-root soldiers. *Military Medical Journal of South China* **23**, 48-49 (2009).
56. Zhou, X. M. Epidemiological investigation on anxiety disorder of public security personnel of Zhongshan. *J Clin Psychosom Dis* **15**, 352-553 (2009).
57. Zou, H. G. *et al.* Epidemiology investigation on mental diseases in Guangzhou military region. *Sichuan Mental Health* **9**, 241-243 (1995).
58. Cao, H., McFarlane, A. C. & Klimidis, S. Prevalence of psychiatric disorder following the 1988 Yun Nan (China) earthquake--the first 5-month period. *Soc Psychiatry Psychiatr Epidemiol* **38**, 204-212; doi:10.1007/s00127-003-0619-2 (2003).
59. Chen, H. *et al.* The presence of post-traumatic stress disorder symptoms in earthquake survivors one month after a mudslide in southwest China. *Nurs Health Sci* **16**, 39-45; doi:10.1111/nhs.12127 (2014).
60. Cui, L. J. *et al.* A cross-sectional study on the mental status of survivors 1 month after earthquake in An county of Sichuan. *Chinese Journal of Nervous and Mental Diseases* **35**, 300-301 (2009).
61. Dong, Q. L. *et al.* The study of post traumatic stress disorder，anxiety and depression on different groups after the Yushu earthquake. *J Clin Psychiatry* **24**, 80-82 (2014).
62. Dong, Q. L. *et al.* The study of psychological situation on different people in different stages after the earthquake. *Chongqing Medicine* **43**, 962-964 (2014).
63. Dong, Y., Qi, J. L., Yang, Z., Guo, L. & Huang, Z. F. Epidemiological survey on the prevalence of posttraumatic stress disorder in male rescue servicemen. *People's Military Surgeon* **53**, 825-827 (2010).
64. Fu, J. *et al.* Posttraumatic stress disorder of women after Wenchuan earthquake. *Journal of Practical Obstetrics and Gynecology* **24**, 744-746 (2008).
65. Fu, Y. *et al.* Analysis of prevalence of PTSD and its influencing factors among college students after the Wenchuan earthquake. *Child Adolesc Psychiatry Ment Health* **7**, 1; doi:10.1186/1753-2000-7-1 (2013).
66. Gao, H. S. *et al.* A follow- up study of post- traumatic stress disorder of SARS patients after discharge/GAO Hongsheng. *Chinese Journal of Rehabilitation Medicine* **21**, 1003-1026 (2006).

67. Gu, K. S. *et al.* Prevalence and correlated factors of PTSD in China rescue cadets after the Wenchuan earthquake. *Mil Med Sci* **35**, 685-699 (2011).
68. Guo, J. *et al.* Post-traumatic stress disorder among adult survivors of the Wenchuan earthquake in China: a repeated cross-sectional study. *J Anxiety Disord* **28**, 75-82; doi:10.1016/j.janxdis.2013.12.001 (2014).
69. Hu, T. C., Yang, X. & Wang, X. Z. Epidemiological survey on adolescent mental illneas in Qingchuan earthquake disaster area. *China Journal of Health Psychology* **19**, 933-934 (2011).
70. Jia, Z. B., Duan, G. F., Zhang, W., Tian, W. H. & Liu, W. Z. Life quality of child and adolescent survivors of Wenchuan earthquake: A follow-up study *Chinese Jounal of Social Medicine* **31**, 403-406 (2014).
71. Kun, P. *et al.* Prevalence of post-traumatic stress disorder in Sichuan Province, China after the 2008 Wenchuan earthquake. *Public Health* **123**, 703-707; doi:10.1016/j.puhe.2009.09.017 (2009).
72. Lau, J. T. *et al.* Psychological distress among adolescents in Chengdu, Sichuan at 1 month after the 2008 Sichuan earthquake. *J Urban Health* **87**, 504-523; doi:10.1007/s11524-010-9447-3 (2010).
73. Li, Y. G., Shi, W., Hu, Y. L., Wang, Z. & Su, L. C. Post-traumatic stress disorder in the wounded half a year after earthquake *J Clin Psychiatry* **20**, 179-181 (2010).
74. Liu, X. *et al.* A risk score for predicting post-traumatic stress disorder in adults in a Chinese earthquake area. *J Int Med Res* **40**, 2191-2198 (2012).
75. Liu, Z. Y. *et al.* One-year follow-up study of post-traumatic stress disorder among adolescents following the Wen-Chuan earthquake in China. *Biosci Trends* **4**, 96-102 (2010).
76. Lv, Y. *A study on the post-traumatic stress disorder of volunteers in Chongqing areas 19 months after Wenchuan earthquakes* master thesis, Southwest University (2010).
77. Ma, X. *et al.* Risk indicators for post-traumatic stress disorder in adolescents exposed to the 5.12 Wenchuan earthquake in China. *Psychiatry Res* **189**, 385-391; doi:10.1016/j.psychres.2011.02.016
10.1016/j.psychres.2010.12.016 (2011).
78. Qi, J. L. *et al.* Study on post-traumatic stress disorder in male rescuer in the 3th months and in the 6th month after Wenchuan earthquake. *China Journal of Health Psychology* **19**, 670-672 (2011).
79. Ruan, Z. R., Mo, K. Y., Dai, K. R. & Wang, R. M. A study of prevalence of stress disordes after a violent earthquake in Wenchuan. *Jounal of Practical Mental Techniques* **16**, 179-180 (2009).
80. Song, D. Y. *et al.* Prevalence and impact factors of PTSD in Chinese military rescuers after Wenchuan earthquake: A two-year follow-up study. *Mil Med Sci* **35**, 573-577 (2011).
81. Wang, H. *et al.* Identification of post traumatic stress disorder and risk factors in military first responders 6 months after Wen Chuan earthquake in China. *J Affect Disord* **130**, 213-219; doi:10.1016/j.jad.2010.09.026 (2011).
82. Wang, Y. Q., Ning, N., Liu, H. & Wang, Y. Q. Post-traumatic Stress Disorder of 76 Wounded Patients in Lushan Earthquake. *Journal of Nursing（China）* **21**, 9-11 (2014).
83. Wei, P. K. *et al.* Investigation on the PTSD incidence of13893 cases of Wenchaun earthquake victims and the rescue solders. *Modern Preventive Medicine* **37**, 3286-3287 (2010).
84. Wu, J. F. *et al.* Posttraumatic stress disorder and related factors analysis of the hospitalized earthquake-hit wounded or disabled patients *Practical Journal of Clinical Medicine* **6**, 25-26 (2009).
85. Wu, Z., Xu, J. & He, L. Psychological consequences and associated risk factors among adult survivors of the 2008 Wenchuan earthquake. *BMC Psychiatry* **14**, 126; doi:10.1186/1471-244X-14-126 (2014).
86. Xiang, Y. J. *et al.* Prevalence of post-traumatic stress disorder symptoms among middle school students after Wenchuan earthquake. *Chinese Mental Health*

Journal **24**, 17-20 (2010).
87. Yang, X., Hu, T. C. & Guo, L. R. Epidemiological survey on elder people's mental illneas in Qingchuan earthquake disaster area. *Chinese Journal of Gerontology* **31**, 1835-1836 (2011).
88. Yang, Y. F. *et al.* A follow-up study on the post-traumatic stress disorders among middle school students in Wenchuan earthquake region. *Zhonghua Yu Fang Yi Xue Za Zhi* **45**, 354-358 (2011).
89. Yang, Y. F., Ye, Y. L., Li, T., Liu, X. X. & Yuan, P. Mental health status among middle school students in Wenchuan earthquake region. *Zhonghua Yu Fang Yi Xue Za Zhi* **44**, 134-139 (2010).
90. Yuan, K. C. *et al.* Prevalence and predictors of stress disorders following two earthquakes. *Int J Soc Psychiatry* **59**, 525-530; doi:10.1177/0020764012453233 (2013).
91. Zhang, B. *et al.* A Study on the Prevalence of Neurosis and Ist Cause over 20 years - After the Violent Earthquake in Tangshan. *Nervous Diseases and Mental Health* **1**, 8-11 (2001).
92. Zhang, B. *et al.* A study of the prevalence of post-traumatic stress disorder after violent earthquake in Tangshan. *Chin J Psychiatry* **32**, 106-108 (1999).
93. Zhang, B. *et al.* A Cross-sectional Study on the Current Prevalence of Posttraumatic Stress Disorder in Adults Orphaned byTangshan Earthquake in 1976. *Chinese Mental Health Journal* **22**, 469-473 (2008).
94. Zhang, J. J. *et al.* Posttraumatic stress disorder in rescue servicemen after Wenchuan earthquake: a follow-up study. *ACTA ACADEMIAE MEDICINAE MILITARIS TERTIAE* **32**, 2326-2328 (2010).
95. Zhang, Y. H., Zhang, X. J. & Sheng, L. Epidemiological survey on mental diseases afer earthquakes *J Clin Psychiatry* **19**, 396 (2009).
96. Zhang, Z. *et al.* Prevalence of post-traumatic stress disorder among adolescents after the Wenchuan earthquake in China. *Psychol Med* **42**, 1687-1693; doi:10.1017/S0033291711002844 (2012).
97. Zhang, Z., Shi, Z., Wang, L. & Liu, M. One year later: Mental health problems among survivors in hard-hit areas of the Wenchuan earthquake. *Public Health* **125**, 293-300; doi:10.1016/j.puhe.2010.12.008 (2011).
98. Zhang, Z., Shi, Z., Wang, L. & Liu, M. Post-traumatic stress disorder, anxiety and depression among the elderly: a survey of the hard-hit areas a year after the Wenchuan earthquake. *Stress Health* **28**, 61-68; doi:10.1002/smi.1403 (2012).
99. Zhao, G. F. *et al.* A cross-sectional study on the mental status of 780 survivors after Wenchuan earthquake *Chin J Evid-based Med* **8**, 815-819 (2008).
100. Zheng, Y., Fan, F., Liu, X. & Mo, L. Life events, coping, and posttraumatic stress symptoms among Chinese adolescents exposed to 2008 Wenchuan Earthquake, China. *PLoS One* **7**, e29404; doi:10.1371/journal.pone.0029404 (2012).
101. Zhou, X. *et al.* Prevalence and risk factors of post-traumatic stress disorder among adult survivors six months after the Wenchuan earthquake. *Compr Psychiatry* **54**, 493-499; doi:10.1016/j.comppsych.2012.12.010 (2013).
102. Zhou, X. R., Zhou, J. & Shi, Q. J. Half-a-year transitional survey on posttraumatic stress disorder symptoms among medical staff in the earthquake region. *Neural Injury and Functional Reconstruction* **5**, 271-276 (2010).
103. Chen, Y. & Fan, Z. Q. Prevalence survey of anxiety and depression among outpatients in department of acupuncture and moxibustion in general hospital *World Chinese Medicine* **8**, 565-567 (2013).
104. Deng, Y. M., Wang, J. H., He, S. L. & Wang, H. Survey and analysis of adult outpatients' dental anxiety in Chongqing hospital of stomatology. *Jounal of Chongqing Medical* **37**, 918-920 (2012).
105. Fu, Y., Geng, L. & Sheng, L. Prevalence of mental disorders in outpatients consulting gynecologist *Chinese Mental Health Journal* **21**, 271-279 (2007).
106. He, R. *et al.* Prevalence of anxiety disorders of outpatients in internal medicine departments of general hospitals at different level *Chin J Public Health* **24**, 702-704 (2008).

107. He, X. Y. *et al.* A Cross-sectional Study on the prevalence of depression and/or anxiety disorders in the emergency patients in a general hospital in Shanghai *Journal of Bethune Medical Science* **12**, 556-557 (2014).
108. He, Y. L. *et al.* A cross-sectional survey of the prevalence of depressive-anxiety disorders among general hospital outpatients in five cities in China. *Zhonghua Nei Ke Za Zhi* **48**, 748-751 (2009).
109. He, Y. L. *et al.* Prevalence of anxiety disorders among outpatients in general hospitals. *Chinese Mental Health Journal* **26**, 165-170 (2012).
110. Huang, S. W., Huang, J. R., Zhu, X. D., Chu, J. & Huang, M. X. Survey on inpatient with mixed anxiety and depressive disorders in comprehensive hospitals. *J Mod Med Health* **31**, 360-362 (2015).
111. Huang, Y. L. *et al.* Survey of depression and /or anxiety disorders among outpatients in general hospitals of Chengdu *J chin Psychiatry* **21**, 260-262 (2011).
112. Jin, Q. *Study of mental illness in internal medicine and traditional Chinese medicine of the general hospital in Shenyang* master thesis, China Medical University, (2006).
113. Li, G., Jiang, R. H., Guo, C. J., Liu, M. Y. & Zhang, L. J. Prevalence of depressive and anxiety disorders in cardiovascular outpafients from 14 tertiary general hospitals of 5 Chinese cities. *Chin J Cardiol* **42**, 1035-1038 (2014).
114. Li, J. L. *et al.* Prevalence of anxiety disorders in outpatient departments of general hospitals in Beijing. *Chinese General Practice* **16**, 1173-1175 (2013).
115. Li, L. M. *et al.* A survey of depressive and anxiety disorder in outpatients in general hospital. *Ningxia Med J* **33**, 861-862 (2011).
116. Li, X. *et al.* Mood disorders investigation and analysis of 520 cases in gynecology outpatients. *Journal of Aerospace Medicine* **22**, 533-536 (2011).
117. Li, X. *et al.* Investigation on incidence of depression/anxiety in gynecologic outpatients and cognition by gynecologists. *Chinese General Practice* **12**, 2144-2147 (2009).
118. Li, X. J., Sun, H. B., Huang, Y. L. & Zhang, L. Prevalence survey of depression and/or anxiety among clinic clients in neurology departments of the third grade com prehensive hospitals in Chengdu. *Practical Journal of Clinical Medicine* **8**, 64-66 (2011).
119. Li, X. J., Zhang, L. & Li, B. Prevalence of depression and (or) anxiety disorders among outpatients in general hospital in Chengdu. *West China MEdical Journal* **26**, 192-194 (2011).
120. Li, Z., Li, Y. H., Guo, H. R., Cao, S. X. & Song, X. Q. Study on the clinical characteristics of neuroses in psychiatry department of general hospital *China Journal of Health Psychology* **17**, 782-783 (2009).
121. Lihua, M. *et al.* Obsessive-compulsive disorder in general hospital outpatients: prevalence, correlates, and comorbidity in Lanzhou, China. *Asia Pac Psychiatry* **6**, 308-318; doi:10.1111/appy.12113 (2014).
122. Liu, Y. M. & Ma, X. J. The distribution of mental diseases in the first outpatients in psychiatric specialist clinic. *Health Psychology Journal* **9**, 199-300 (2001).
123. Lubetkin, E. I., Jia, H. & Gold, M. R. Depression, anxiety, and associated health status in low-income Chinese patients. *Am J Prev Med* **24**, 354-360 (2003).
124. Ma, L. H. *et al.* Cross-sectional study of neuroses among outpatients in general hospitals of Xi'an. *Journal of Nursing (China)* **20**, 62-65 (2013).
125. Mei, L. *Survey on the prevalence of anxiety disorders of internal medical outpatients in general hospital* master thesis, Shanghai Jiao Tong University (2009).
126. Mei, L., Xiao, Z. P., He, Y. L. & Fan, Q. Survey on the prevalence of anxiety disorders in internal medical outpafients at general hospital. *Natl Med J China* **90**, 3172-3175 (2010).
127. Ni, C. *et al.* Screening and correlates of neurotic disorders among general medical outpatients in Xi'an China. *Perspect Psychiatr Care* **51**, 128-135; doi:10.1111/ppc.12074 (2015).
128. Ni, Y. *et al.* Cross-sectional study of anxiety disorders among non-psychiatric

outpatients in general hospital *Chinese Mental Health Journal* **25**, 801-805 (2011).
129. Peng, H. L. *et al.* A survey on the prevalence of depressive and / or anxiety disorder of outpatients in Changsha general hospitals *Chinese Journal of Clinical Psychology* **16**, 300-330 (2008).
130. Qin, X. *et al.* Prevalence and rates of recognition of anxiety disorders in internal medicine outpatient departments of 23 general hospitals in Shenyang, China. *General hospital psychiatry* **32**, 192-200; doi:10.1016/j.genhosppsych.2009.12.001 (2010).
131. Qin, X. X. *et al.* Prevalence of anxiety disorders and features in general hospitals in Shenyang. *Chinese General Practice* **10**, 899-901 (2007).
132. Qu, L. M. *et al.* Epidemic Inventory of Depression and Anxiety Disorders in Out-patient Departments of Comprehensive Hospitals in Guangzhou District. *Chinese Journal of Clinical Psychology* **17**, 61-63 (2009).
133. Sha, L. *Panic attack and anxiety disorder in outpatients* master thesis, Fudan University, (2009).
134. Shi, L. L. *et al.* Occurrence of depressive and/or anxiety disorders in neurological outpatients from general hospital in Beijing. *Chinese Mental Health Journal* **23**, 616-620 (2009).
135. Song, W. X. *et al.* Investigation on the prevalence of depression and anxiety disorder in patients with cardiovascuIar disease in hospitals at primary level. *Journal of Chongqing Medical University* **37**, 911-913 (2012).
136. Tan, S. Y. *Prevalence survey of mental disorders and the characteristics analysis of suicide attempters of the emergency departments in general hospitals* master thesis, China Medical University, (2008).
137. Tang, M. N. *et al.* in *The 9th Chinese Medical Association National Psychiatry Medicine Academic Conference* (Chinese Conference Database, Guangzhou, Guangdong 2011).
138. Tucci, D. L. & Rubel, E. W. Physiologic status of regenerated hair cells in the avian inner ear following aminoglycoside ototoxicity. *Otolaryngol Head Neck Surg* **103**, 443-450 (1990).
139. Wang, W. *Prevalence and rates of recognition of anxiety disorders in internal medicine outpatient departments of general hospitals in Shenyang* master thesis, China Medical University (2007).
140. Wang, W., Xu, B., Luan, R., Zhan, S. & et al. Study of depressive and anxiety disorder in menopausal gynecologic outpatients in three Chinese cities. *Wei Sheng Yan Jiu* **37**, 211-213 (2008).
141. Wang, X. L. *et al.* Prevalence study of climacteric anxiety and depression in general hospital *Maternal and Child Health Care of China* **22**, 1596-1598 (2007).
142. Wang, Y. H. *et al.* A Study of emotional disorders in inpatient of neurology. *Health Psychology Journal* **9**, 246-248 (2001).
143. Wu, Y. L. & Ba, H. T. Analysis on prevalence of depression and (or) anxiety disorders among outpatients in general hospital. *World Latest Medicine Information* **15**, 70-71 (2015).
144. Xie, D. S., Zhu, Y. & Hou, X. G. Investigation of the prevalence in the outpatients of department of gastroenterology *Medical Information* **23**, 1857-1858 (2010).
145. Ying, D. G., Jiang, S., Yang, H. & Zhu, S. Frequency of generalized anxiety disorder in Chinese primary care. *Postgrad Med* **122**, 32-38; doi:10.3810/pgm.2010.07.2173 (2010).
146. Zhang, M. in *The 9th Chinese Medical Association National Psychiatry Medicine Academic Conference* (Chinese Conference Database, Guangzhou, Guangdong 2011).
147. Zhang, T. *Investigating mental disturbances among inpatients in a general hospital and the development of a detecting tool* master thesis, Central South Universty, (2014).
148. Zhang, Z. F. *et al.* Statistics analysis of clinical diagnosis on 1531 the First

outpatients in Psychiatric Specialist Chinic. *Medical Journal of Chinese People's Health* **13**, 65-67 (2001).
149. Zhou, Z. H., Huang, W. J., Jin, S. H., Guo, J. Z. & Chen, S. Y. Prevalence and management of mental disorders in general practice clinic of general hospital *Chinese General Practice* **15**, 2942-2945 (2012).
150. Zhu, C. Y. *et al.* Study on the risk factors of dpression / anxiety disorders among medical outpatients of comprehensive hospital in Guangzhou City. *Modern Preventive Medicine* **37**, 491-493 (2010).
151. Lv, C. R. *et al.* Investigation on the Prevalence of mental disorders in prisoners *J Clin Psychol Med* **13**, 226-227 (2003).
152. Zhang, J. Morbidity and trauma characteristic of posttraumatic stress disorder in the female criminals. *Guangxi Medical Journal* **34**, 540-545 (2012).
153. Zhang, J. *et al.* The epidemiological study of mental disorder among female criminals in a prison in GuangXi Zhuang Nationality Autonomous Region. *Journal of International Psychiatry* **40**, 199-202 (2013).
154. Zhao, S., Sun, C. M. & Yu, Y. W. An epidemiological study of mental disorders among jail termers. *J Chin Psychosom Dis* **15**, 508-509 (2009).
155. Zhao, S., Yuan, Y. G., Cui, Z. J. & Sun, C. M. An epidemiological study of mental disorders among male criminals in Jiangsu. *Jounal of Clinical Medicine in Practice* **14**, 152-160 (2010).
156. Zhou, J. S. *et al.* Prevalence of mental disorders in the male juvenile detention centers of Hunan and Sichuan *J Cent South Univ (Med Sci)* **37**, 217-221 (2012).
157. Zhou, J. S. *et al.* Prevalence of mental disorders in the male juvenile delinquents, Changsha and Chengdu. *Chinese Journal of Clinical Psychology* **20**, 53-55 (2012).
158. Hou, C. L. *The epidemiology and neuroimaging research in coal mining accident surviors wiht posttraumatic stress disorder* doctor thesis, Jilin University (2007).
159. Li, H. B. *Research on post-traumatic stress disorder for miners after the coalmine disasters* doctor thesis, Jilin University (2011).
160. Liao, J. X., Yang, C. H., Zhang, Z. M. & Hu, P. L. Investigation on post-traumatic stress disorder in injured coal miners. *China Journal of Health Psychology* **20**, 1797-1799 (2012).
161. Ni, Y. L. Epidemiological survey on the prevalence of mental diseases in Guiyang steelworkers. *Chinese Medical Journal of Metallurgical Industry* **12**, 25-27 (1995).
162. Sun, Z. L. *et al.* Epidemiological survey on the prevalence of mental diseases among miners in Anhui, Huaibei. *J chin Psychiatry* **21**, 181-183 (2011).
163. Tang, M. Q. *et al.* A survey on psychological status of population in lead mining area in Ningnan county *Journal of Occupational Health and Damage* **5**, 7-11 (1990).
164. Wang, B. J. Epidemiological survey on prevalence of mental diseases in Shengli oil field in 1996. *J chin Psychiatry* **22**, 72-74 (1996).
165. Wang, H. H. *et al.* A preliminary survey on psychological status of the7.29 coalmine mishap survivors *Chin J Nerv Ment Dis* **35**, 432-434 (2009).
166. Wang, Q. S., Yu, H. Y., Chen, S. D. & Han, Y. H. Epidemiological survey on the prevalence of mental diseases stuff in railway department *Journal of Qiqihar University of Medicine* **23**, 205-206 (2002).
167. Chen, L. *et al.* Prevalence and determinants of chronic post-traumatic stress disorder after floods. *Disaster Med Public Health Prep* **9**, 504-508; doi:10.1017/dmp.2015.64 (2015).
168. Gu, Y. *et al.* Relationship between domestic child abuse and mental health in children aged 10-13. *Chin J Behav Med & Brain Sci* **21**, 1128-1130 (2012).
169. Han, L., Su, N., Liu, T. B. & Yang, D. S. The comparasion the prevalence of population in factory and of population in rural *Journal of Clinical Psychiatry* **3**, 36-38 (1993).
170. He, J. Y., Xie, L. N., Deng, X. Y., Qiu, J. H. & Zhang, X. Q. Research on

PTSD and its causes of freshmen's parents after college entrace examinations. *Chin J School Doctor* **29**, 92-95 (2015).
171. Jia, S. S., Yang, J. M. & Tian, G. R. Investigation on the prevalence of neurosis among population abused in Internet chatting *Journal of Medical Forum* **9**, 59-60 (2001).
172. Jiang, C. L. *et al.* Incidence and influencing factor analysis of PTSD after major disasters. *Medical Journal of Chinese People's Health* **26**, 9-12 (2014).
173. Lai, Q. P., Jiang, H. & Xie, X. L. Research and analysis of the prevalence of prenatal depression and risk factors. *Jilin Med* **35**, 6901-6903 (2014).
174. Li, R. X. *et al.* Physical and mental health status of 40-55 year women in Shanghai *Maternal and Child Health Care of China* **25**, 3911-3913 (2010).
175. Lin, L. *Research on post-traumatic stress disorder of emergency nurses in third class of general hospital of Changsha* master thesis, Central South University (2007).
176. Luan, Z. Y. & Miao, N. A survey on post-traumatic stress disorder of ICU nurses. *Jounal of Nursing Science* **30**, 84-86 (2015).
177. Mai, J. W., Zhou, R. & Li, L. F. Investigation of acute posttraum atic stress disorder in patients with head injury after traffic acciden. *Chinese Journal of Clinical Rehabilitation* **9**, 54-55 (2005).
178. Shen, L. L. *et al.* A survey of anxiety and depression symptoms among primary-care physicians in China. *Int J Psychiatry Med* **44**, 257-270 (2012).
179. Shi, S. X. *et al.* A survey of anxiety and depression among involutional people. *Shanghai Mental Med* **11**, 208-211 (1999).
180. Song, F. Y., Li, P., Liu, Y. Y. & HU, K. Comparative study on incidence of post-traumatic stress disorder and influential factors of women of Han and Miao in Guizhou. *Jilin Med* **34**, 1285-1288 (2013).
181. Sun, X. L. *et al.* The prevalence of suicide attempters in people aged 18 and older in Hebei Province *Chinese Mental Health Journal* **24**, 362-365 (2010).
182. Sun, Y. P. *et al.* Psychiatric state of college students with a history of childhood sexual abuse. *World J Pediatr* **4**, 289-294; doi:10.1007/s12519-008-0052-4 (2008).
183. Tang, M. Q. Survey on mental status in booze-mining regions in Ningnan county *Journal of Occupational Health and Damage* **5**, 7-11 (1990).
184. Wang, L. N. *et al.* A cross-sectional study of neurological disease in the veterans of military communities in Beijing. *Chin J Intern Med* **49**, 463-468 (2010).
185. Wang, X. Y. & Zhang, G. Q. Occurrence of posttraumatic stress disorder(PTSD) in trauma-experienced inpatients and influencing factors. *China Journal of Health Psychology* **22**, 812-814 (2014).
186. Wei, Z. *et al.* Service utilization for mental problems in a metropolitan migrant population in china. *Psychiatr Serv* **64**, 645-652; doi:10.1176/appi.ps.201200304 (2013).
187. Wong, F. K., Chang, Y. L. & He, X. S. Correlates of psychological wellbeing of children of migrant workers in Shanghai, China. *Soc Psychiatry Psychiatr Epidemiol* **44**, 815-824; doi:10.1007/s00127-009-0003-y (2009).
188. Wu, P. P., Zhan, X. H., Yang, L. P., Yan, G. L. & Song, P. The clinical epidemiological Investigation into 258 cases of premenstrual dysphoric disorder *Maternal and Child Health Care of China* **28**, 2254-2257 (2013).
189. Xun, M. L. *et al.* Epidemiological survey on prevalence of neurosis in females after sterilization in Xiangtan area. *Medical Journal of Chinese People's Health* **6**, 130-133 (1994).
190. Yao, Y. S. *et al.* A prevalence survey on the mental health of left behind adolescent in Anhui Province. *Zhonghua Liu Xing Bing Xue Za Zhi* **31**, 1359-1362 (2010).
191. Chan, C. H., Tiwari, A., Fong, D. Y. & Ho, P. C. Post-traumatic stress disorder among Chinese women survivors of intimate partner violence: a review of the literature. *Int J Nurs Stud* **47**, 918-925; doi:10.1016/j.ijnurstu.2010.01.003 (2010).

192. Hong, C. & Efferth, T. Systematic Review on Post-Traumatic Stress Disorder Among Survivors of the Wenchuan Earthquake. *Trauma Violence Abuse*; doi:10.1177/1524838015585313 (2015).
193. Lang, T. J. Analysis and countermeasures of middle school students' social phobia. *The Science Education Article Collects*, 196-204 (2014).
194. Li, Y. *et al.* A meta-analysis on the prevalence of post-traumatic stress disorder in children and adolescents. *Chin J Trauma* **30**, 1075-1081 (2014).
195. Shen, S. L., Zhang, C. Y. & Wang, Y. P. The present status of studies on post-traumatic stress disorder after serious natural disasters *Health Vocational Education* **32**, 158-159 (2014).
196. Su, L., Cai, Y. Y., Shi, S. X. & Wang, L. W. A meta analysis of prevalence in anxiety disorders of elderly people in china *J clin Psychiatry* **21**, 87-90 (2011).
197. Wang, L. L. Analysis on the situation of prevalence of neurosis and psychiatric disorders in the adolescents. *Modern Journal of Integrated Traditional Chinese and Western Medicine* **14**, 2447 (2005).
198. Xue, C. *et al.* A meta-analysis of risk factors for combat-related PTSD among military personnel and veterans. *PLoS One* **10**, e0120270; doi:10.1371/journal.pone.0120270 (2015).
199. Bu, T. T. Research progress of post-traumatic stress disorder. *Medical Journal of the Chinese People's Armed Police Forces* **21**, 537-539 (2010).
200. Fan, Q. Research process on the Epidemiologicology of anxiety disorders *Chin J Psychiatry* **43**, 183-186 (2010).
201. Hu, Q. *et al.* Prevalence of anxiety disorder among mainland residents in China：a meta-analysis. *Chin J Psychiatry* **46**, 204-211 (2013).
202. Huang, Y. Q. Epidemiological study on mental disorder in China *Chin J Epidemiol* **33**, 15-16 (2012).
203. Li, Y. J., Tang, Y. L. & Cai, Z. J. Research process on social anxiety disorder. *Chinese Mental Health Journal* **15**, 184-185 (2001).
204. Liao, Z. H., Ding, L. J. & Wen, C. Study of Epidemiological Survey of Mental Disorders in China *Chinese General Practice* **15**, 1160-1175 (2012).
205. Su, L. & Wei, B. The Condition of Study of Epidemiological Survey of Mental Disorders in China *Internal Medicine of China* **15**, 416-419 (2010).
206. Wang, X., Fan, J. L. & Ling, Y. Review of social phobia *Chin J SchoolDocto* **22**, 473-475 (2008).
207. Wang, X. D. Epidemiological characters and risk factors on post-traumatic stress disorder. *Chin J Epidemiol* **23**, 334-337 (2002).
208. Wang, Z. Y. Research progress of post-traumatic stress disorder in domestic. *Shanghai Archives of psychiatry* **18**, 372-379 (2006).
209. Xiong, J. W. Research status on obsessive compulsive disorder. *Medical Journal of Chinese People's Health* **24**, 577-580 (2012).
210. Yue, L. L. & Bo, G. Z. Research Status of Depression and Anxiety Disorders *Medicine Recapitulate* **19**, 1069-1072 (2013).
211.Zhao, X. D. Research status on the prevalence of mental disorders. *Hebei Medical Journal* **30**, 84-85 (2008).
212. Zhong, B. L., Zhang, J. F., He, F. M., Huang, Y. Q. & Chen, H. H. Methodology improvement in China mental disorder epidemiological surveys from 1950 to 2008. *Chin J Psychiatry* **43**, 235-240 (2010).
213. Deng, X. F. Epidomiological survey of the mental illness in leshan city of Sichuan Province. *Medical Journal of Chinese People Health* **15**, 265-271 (2003).
214. Guo, H. L. Present and future state of mental health of Beijing in 1990s. *Journal of Clinical Psychiatry* **4**, 131-134 (1994).
215. Li, D. G. *et al.* The epidemiologic survey of the mental disorder in the Kerean adults in Helong county. *Jounal of Yanbian Medical Colleage* **15**, 134-137; doi:10.16068/j.1000-1824.1992.02.022 (1992).
216. Li, D. G. *et al.* Epidemiological Study of Mental Disorders in Koreans-Comparison of Lifetime Prevalence in Yanji City of China and Kangwha of Korea. *Jounal of Yanbian Medical Colleage* **13**, 132-140 (1990).
217. Li, D. G., Jin, Y. F. & Piao, Y. S. The Epidemical Survey Of Neurosis In

Yanbian Area in Korean Population. *Journal of Yanbian medical college* **1**, 46-49 (1990).
218. Li, D. G. *et al.* Epidemiological survey on lifetime prevalence of mental disorders in Yanji adults. *Chinese Journal of Nervous and Mental Diseases* **18**, 159-192 (1992).
219. Wang, B. Z. An epidemiological survey of neurosis among Bai minority in Dali of Yunnan Province *Journal of Clinical Psychiatry* **7**, 88-89 (1997).
220. Xiang, M. Z. *et al.* Epidemiological Survey on mental diseases in Dongcheng area and Xinjin county of Chengdu. *Sichuan Medicine* **8**, 67-69 (1987).
221. Yu, S. C., Song, Y., Liu, X. W. & Zhao, Y. Z. Survey on the prevalence of neurosis in Mudanjiang City comparing with other 12 domestic areas *Chinese Mental Health Journal* **18**, 348-384 (1992).
222. Zhao, H., Zhao, D. Q., Zhu, S. Y. & Lin, Y. Q. Epidemiological survey on neurosis in Shantou City. *Medical Journal of Chinese People's Health* **12**, 80-81 (2000).
223. Xu, G. M. *et al.* in *The 10th Chinese Medical Association National Psychiatry Medicine Academic Conference* (Chinese Conference Database, Naning, Jiangsu, 2012).
224. Zhang, H. Y. *et al.* Epidemiological survey on neuroses in Shenzhen City. *Chin J Public Health* **22**, 866-867 (2006).
225. The psychiatric disease prevention groups in Xuhui District,Shanghai. Epidemiological survey on the mental disorders prevalence in Xuhui District,Shanghai from 1979~1984. *Shanghai Archives of Psychiatry*, 14-17 (1986).
226. Mo, S. L. *et al.* Investigation on psychiatric epidemiology in Mengshan county of Guangxi *Jounal of Guilin medical college* **9** (1996).
227. Wu, C. D. *et al.* The epidemical survey on neurosis in the Hui, Li and Han nationality in Yanglan county of Sanya. *Medical Journal of Chinese People's Health* **supplement**, 19-20 (1994).
228. Zhong, W. *et al.* The epidemiologic survey of the mental disorder in Panyu district of Guangzhou. *Journal of International Psychiatry* **38**, 138-141 (2011).
229. Zhou, T. X., Zhang, S. P., Jiang, Y. Q. & Wang, J. M. Epidemiology of neuroses in community of Shanghai. *Chinese Mental Health Journal* **14**, 332-334; doi:10.3321/j.issn:1000-6729.2000.05.020 (2000).
230. Li, A. Y. *et al.* Epidemiological study of mental and behavior disorders in Jinuo nationality. *J Clin Psychosom Dis* **21**, 88-93 (2015).
231. Li, Z. *et al.* Epidemiological investigation on neurotic disorder in Zhongshan. *J Clin Psychol Med* **14**, 145-146 (2004).
232. Zhang, M. L. *et al.* Epidemiological survey on neurosis in Wuxi City. *J Clin Psychol Med* **15**, 78-79 (2005).
233. Phillips, M. R. *et al.* Prevalence, treatment, and associated disability of mental disorders in four provinces in China during 2001-05: an epidemiological survey. *Lancet* **373**, 2041-2053; doi:10.1016/S0140-6736(09)60660-7 (2009).
234. Ding, Z. J. *et al.* Epidemiological survey of mental disorders in people aged 18 and older in Tianshui City of Gansu Province. *Chinese Mental Health Journal* **24**, 183-190 (2010).
235. Feng, S. T. *et al.* Epidemiological survey of mental illnesses in persons aged 18 years and older in Qingdao City. *Chinese Mental Health Journal* **24**, 175-182 (2010).
236. Shi, Q. C. Epidemiological survey of mental illness in the people aged 15 and older in Zhejiang Province. *Chin J Prev Med* **39**, 229-236 (2005).
237. Song, Z. Q. *et al.* Epidemiological survey of mental disorders in persons aged 18 and older in Qinghai Province. *Chinese Mental Health Journa* **24**, 168-190 (2010).
238. Zhang, J. X. *et al.* Epidemiological survey of mental disorders in persons aged 18 years and older in Shandong Province. *Chinese Mental Health Journal* **24**, 161-182 (2010).
239. Gao, Z. Z. *et al.* The Third epidemiology investigation of mental illness in

Weifang City Shandong Province. *Chin J N erv Ment Dis* **32**, 152-153 (2006).
240. Liu, T. H., Sun, S. X. & Chen, Y. H. Survey on prevalence of mental disorders in Laiwu City. *Shandong Arch Psychiatry* **14**, 124-125 (2001).
241. Meng, G. Y. *et al.* Epidemiological sampling survey of mental disorders in populations aged 18 years and above in Jinan. *Journal of Clinical Psychiatry* **24**, 176-179 (2011).
242. Sun, Y. M. A survey on mental illness in Liaocheng City, Shandong Province. *Chin J Epidemiol* **30**, 978-979 (2009).
243. Yu, S. F. & Sun, Y. M. The Third epidemiology investigation of mental illness in Liaocheng City of Shandong Province. *Nursing Practice and Research* **9**, 156-157 (2012).
244. Zhang, S. D. *et al.* Epidemiological survey of mental disorders in adults aged 18 years and above in Yantai. *Jounal of Psychiatry* **24**, 350-352; doi:10.3969/j.issn.1009-7201.2011.05.011 (2011).
245. Demyttenaere, K. *et al.* Prevalence, severity, and unmet need for treatment of mental disorders in the World Health Organization World Mental Health Surveys. *JAMA* **291**, 2581-2590; doi:10.1001/jama.291.21.2581 (2004).
246. Shen, Y. C. *et al.* Twelve-month prevalence, severity, and unmet need for treatment of mental disorders in metropolitan China. *Psychol Med* **36**, 257-267; doi:10.1017/S0033291705006367 (2006).
247. Lee, S. *et al.* Lifetime prevalence and inter-cohort variation in DSM-IV disorders in metropolitan China. *Psychol Med* **37**, 61-71; doi:10.1017/S0033291706008993 (2007).
248. Huang, Y. Q. *et al.* Epidemiological survey of common mental disorders in Beijing. *Chinese Medical Journal* **124**, 169 (2011).
249. Liu, Z. R., Huang, Y. Q., Chen, X., Cheng, H. & Luo, X. M. The prevalence of mood disorder, anxiety disorder and substance use disorder in community residents in Beijing: A cross-sectional study. *Chinese Mental Health Journal* **27**, 102-110; doi:10.3969/j.issn.1000-6729.2013.02.005 (2013).
250. Li, K. Q. *et al.* Epidemiological survey of mental disorders in people aged 18 or over in Hebei Province. *Chin J Psychiatry* **40**, 36; doi:10.3760/j.issn:1006-7884.2007.01.009 (2007).
251. Cong, X. S. *et al.* An epidemiological survey on specific phobia in Baoding. *Journal of Neuroscience and Mental Health* **9**, 325-327 (2009).
252. Li, X. P. Epidemiological sarvey of panic disorder in Baoding. *Chin J Public Health* **24**, 1366-1368 (2008).
253. Li, X. P. *et al.* An epidemioIogical survey of anxiety disorder in Baoding. *J Clin Psychiatry* **18**, 252-254 (2008).
254. Lu, B. H. *et al.* Epiderrdological survey of post-traumatic stress disorder in Baoding City. *J Clin Psychiatry* **18**, 38-40 (2008).
255. Ma, Q. M. *et al.* Epidemiological survey of mental disorders in Baoding *Chinese General Practice* **11**, 522-526 (2008).
256. Su, X. R., Lu, S. L., Su, K. Q. & Li, X. P. The epidemical survey of generalized anxiety disorder in Baoding City. *Nervous Diseases and Mental Health* **8**, 390-392 (2008).
257. Wang, Z. M. Epidemiological Survey of Psychotic Disorders in Baoding City. *China Journal of Health Psychology* **16**, 542-544 (2008).
258. Gao, L. H. *et al.* Epidemiological survey on specific phobias in Hebei Province. *Chinese General Practice* **10**, 1451-1452 (2007).
259. Han, Y. C. *et al.* The cross-sectional survey on the prevalence of panic disorders in Hebei Province *Chin J Epidemiol* **28**, 837 (2007).
260. Jiang, Q. P. *et al.* The Epidemical Survey of Post-traumatic stress disorder in Hebei. *Chinese Mental Health Journal* **22**, 16 (2008).
261. Li, J. F. *et al.* Epidemiological investigation of mental disease in Chengde region of Hebei Province. *J Chin Psychol Med* **18**, 23-25 (2008).
262. Lu, B. H., Wang, C. M., Chen, H. R., Zhang, C. C. & Liu, M. H. Epidemiological study of post-trauma stress disorders comparison of prevalence in different areas. *Journal of Psychiatry* **22**, 373-374 (2009).

263. Sun, X. L. *et al.* Epidemiological survey on generalized anxiety disorder in 4 areas of Hebei Province. *Journal of Clinical Rehabilitative Tissue Engineering Research* **11**, 7842-7844 (2007).
264. Sun, X. L. *et al.* Epidemiological survey on prevalence of anxiety disorders in Hebei Province *Capital Food Medicine* **7** (2009).
265. Diao, W. L. *et al.* Analysis on prevalences of mood disorders,anxiety and alcohol abuse among different occupational populations in Liaoning Province. *Chinese J IndMed* **21**, 325-327 (2008).
266. Liu, H. *et al.* Epidemiological survey on mental disorders in Fuxin,Liaoning. *Chin J Pre Contr Chron Dis* **16**, 484-486 (2008).
267. Ma, Y. S., Liu, L., Yang, S., Zhang, S. J. & Yu, L. Z. Epidemiological survey on mental disorders in Anshan residents, Liaoning *Chin J Pre Contr Chron Dis* **17**, 621-623 (2009).
268. Pan, G. W. *et al.* Epidemiological survey of mental disorders in urban and rural areas of Liaoning province. *Chin J Public Health* **22**, 1505-1507 (2006).
269. Yu, C. Y., Li, N., Zhang, X. X., Wang, L. & Sun, Y. K. Epidemiological survey of mental disorders in rural areas of Qingyua county *Chin J Pre Contr Chron Dis* **17**, 623-625 (2009).
270. Zhang, S. J. *et al.* Prevalence of social phobia among urban and rural residents of Liaoning Province. *Chin J Public Health* **26**, 616-617 (2010).
271. Zhao, H. *et al.* Epidemiological survey on the prevalence of mental diseases among Dawa county residents in Shandong Province *Chin J Public Health* **26**, 760-761 (2010).
272. Li, N. *et al.* Epidemiological survey of anxiety disorders in urban and rural residents of Liaoning Province. *Chin J Prev Contr Chron Non-commun Dis* **16**, 179-181; doi:10.3969/j.issn.1004-6194.2008.02.023 (2008).
273. Li, Y. *et al.* Prevalence of anxiety disorders in Dalian City. *Journal of International Psychiatry* **38**, 76-80 (2011).
274. Mo, X. Y. *Cross-sectional survy on the mental disorders among people in Dalian* Master thesis, Dalian Medical University, (2008).
275. Zhang, X. N. *Study on prevalence of Anxiety disorders and correlation factors in Dalian City* Master thesis, Dalian Medical University, (2010).
276. Chen, Q. *et al.* Epidemiological survey of mental disorders in urban areas in Guangxi Zhuang autonomous region, China. *Chin J Nerv Ment Dis* **36**, 458-462 (2010).
277. Feng, Q. M. *et al.* Mental disorders among minority people in rural areas of Guangxi Zhuang Autonomous Region. *Chin J Public Health* **27**, 408-410 (2011).
278. Gan, J. X., Xiao, X., Hunag, H. B. & Li, J. Epidemiological survey of mental disorders in Yulin, Guangxi *Medical Journal of Chinese People's Health* **20**, 2005-2009 (2008).
279. Tao, L. G. *et al.* Epidemiological survey of mental disorders in urban in Guilin. *Medical Journal of Chinese People's Health* **24**, 527-530 (2012).
280. Tao, G. L. *et al.* Epidemiological survey of mental disorders in urban and rural in Guilin, China *Jounal of Guangxi Medical University* **28**, 797-801 (2011).
281. Wei, B. *et al.* Epidemiology survey on the mental disorders in rural in Guangxi Zhuang Autonomous Region. *Modern Preventive Medicine* **38**, 1801-1805 (2011).
282. Wei, B. *et al.* Epidemiological survey on mental disorders in urban and rural in Guangxi Zhuang autonomous region, China. *Journal of Guangxi medical university* **27**, 951-956; doi:10.3969/j.issn.1005-930X.2010.06.061 (2010).
283. Lu, J. *et al.* Cross-sectional study of anxiety disorders in Kunming. *Chin J Psychiatry* **42**, 34-37 (2009).
284. Ruan, Y. *et al.* Epidemiological survey of mental and behavior disorders in Kunming. *Modern Preventive Medicine* **37**, 628-632 (2010).
285. Liao, Z. H. *et al.* Cross-sectional study on mood disorders, anxiety disorders and substance use disorders among different occupational populations in Xiamen City. *Chin Occup Med* **41**, 46-51 (2014).
286. Wang, W. Q. *et al.* Epidemiological survey of mental disorders in people

aged18 years and older in Xiamen City. *Chin J Psychiatry* **46**, 43-49; doi:10.3760/cma.j.issn.1006-7884.2013.01.013 (2013).
287. Zhong, S. M. *et al.* Epidemiological survey of generalized anxiety disorder in Yunfu City. *Hainan Med J* **26**, 135-136 (2015).
288. Lu, D. Y., Zheng, H. B., Xiao, Y. N., Zhang, L. L. & Zhong, S. M. Epidemiologic survey of anxiety disorders in community population aged 15 and older in Yunfu City *Journal of Clinical Psychiatry* **25**, 107-108 (2015).
289. Hu, J. Z. *et al.* Survey on mental disorders among registered non-residents in Shenzhen. *Chin J Epidemiol* **30**, 543-548 (2009).
290. Duan, W. D. *et al.* The survey on the prevalence and social function of Phobia in Shenzhen City. *Chin J Nerv Ment Dis* **37**, 401-405; doi:10.3969/j.issn.1002-0152.2011.07.006 (2011).
291. Wang, Z., Koenig, H. G., Zhang, Y., Ma, W. & Huang, Y. Religious involvement and mental disorders in mainland china. *PLoS One* **10**, e0128800; doi:10.1371/journal.pone.0128800 (2015).
292. Li, T. *The Investigation of Mental Disorders and Their Impact Factors in Rural Areas Adults of NingXia* Master thesis, Ningxia Medical University (2013).
293. Wei, G. *The epidemiology survey and study of preventive and curative strategies on mental disorders at Tibet in China* doctor thesis, Sichuan University, (2004).
294. Liu, S. M. *et al.* Epidemiological survey on neuropsychiatric disorders in Tibet of China *J Sichuan Univ (Med Sci Edi)* **43**, 210-225 (2012).
